# Striatal BOLD and midfrontal theta power express motivation for action

**DOI:** 10.1093/cercor/bhab391

**Published:** 2021-11-24

**Authors:** Johannes Algermissen, Jennifer C Swart, René Scheeringa, Roshan Cools, Hanneke E M den Ouden

**Affiliations:** Donders Institute for Brain, Cognition and Behaviour, Radboud University, Thomas van Aquinostraat 4, 6525 GD Nijmegen, The Netherlands; Donders Institute for Brain, Cognition and Behaviour, Radboud University, Thomas van Aquinostraat 4, 6525 GD Nijmegen, The Netherlands; Donders Institute for Brain, Cognition and Behaviour, Radboud University, Thomas van Aquinostraat 4, 6525 GD Nijmegen, The Netherlands; Erwin L. Hahn Institute for Magnetic Resonance Imaging, University of Duisburg-Essen, Kokereiallee 7, 45141 Essen, Germany; Donders Institute for Brain, Cognition and Behaviour, Radboud University, Thomas van Aquinostraat 4, 6525 GD Nijmegen, The Netherlands; Department of Psychiatry, Radboud University Medical Centre, Reinier Postlaan 10, 6525 GC Nijmegen, The Netherlands; Donders Institute for Brain, Cognition and Behaviour, Radboud University, Thomas van Aquinostraat 4, 6525 GD Nijmegen, The Netherlands

**Keywords:** motivational biases, simultaneous EEG-fMRI, striatum, theta

## Abstract

Action selection is biased by the valence of anticipated outcomes. To assess mechanisms by which these motivational biases are expressed and controlled, we measured simultaneous EEG-fMRI during a motivational Go/NoGo learning task (*N* = 36), leveraging the temporal resolution of EEG and subcortical access of fMRI. VmPFC BOLD encoded cue valence, importantly predicting trial-by-trial valence-driven response speed differences and EEG theta power around cue onset. In contrast, striatal BOLD encoded selection of active Go responses and correlated with theta power around response time. Within trials, theta power ramped in the fashion of an evidence accumulation signal for the value of making a “Go” response, capturing the faster responding to reward cues. Our findings reveal a dual nature of midfrontal theta power, with early components reflecting the vmPFC contribution to motivational biases, and late components reflecting their striatal translation into behavior, in line with influential recent “value of work” theories of striatal processing.

## Introduction

Learning from rewards and punishments allows us to adapt action selection to our environment. At the same time, our responses are also shaped by seemingly automatic action tendencies that appear to be innate or acquired very early in development. A prime example is motivational action biases (also called “Pavlovian” biases), referring to the tendency to invigorate actions when there is a prospect of reward, but to hold back when there is a threat of punishments (Dayan et al. 2006; Guitart-Masip, Duzel, et al. 2014). Such an action “prior” allows for fast responding, which is often adaptive given that reward pursuit typically requires active responses and threat avoidance typically requires action suppression. However, in environments in which these relationships do not hold, the action triggered by the motivational bias can interfere with the normative optimal action (i.e., the action that maximizes rewards and minimizes punishments) and needs to be suppressed. Keeping a balance between hardwired action tendencies and action values flexibly learned from experience can be challenging, and deficits have been linked to psychiatric disorders such as addiction, depression, trauma symptoms, and social anxiety (Garbusow et al. 2016, 2019; Huys et al. 2016; Mkrtchian et al. 2017; Ousdal et al. 2018). Thus, it is important to understand the mechanism by which humans selectively rely on these biases when they are helpful and suppress them when they are not. In this study, we investigate the neural interactions that accompany (un)successful suppression of motivational biases when needed.

Motivational biases have been hypothesized to arise from dopaminergic effects in the basal ganglia (Frank 2005; Collins and Frank 2014). In line with the putative role of the striatum in driving motivational biases, striatal fMRI BOLD signal has been found modulated by cues signaling the prospect of reward (O’Doherty et al. 2003; Tobler et al. 2007; Niv et al. 2012) and dopaminergic medication has been found to modulate these motivational biases (Guitart-Masip, Chowdhury, et al. 2012; Guitart-Masip, Economides, et al. 2014; Swart et al. 2017; van Nuland et al. 2020).

When actions triggered by motivational biases conflict with the action required to obtain a desired outcome, individuals need to detect this motivational conflict and mobilize control mechanisms to suppress biases. This role has traditionally been attributed to interactions between the medial and lateral prefrontal cortex, particularly the anterior cingulate cortex (ACC). More recent approaches regard the medial frontal cortex, in particular the ACC, as a central decision hub evaluating whether to recruit cognitive control or not (Shenhav et al. 2013, 2016). Recruiting cognitive control is perceived as costly, but potentially worth the effort to overcome biases and select the optimal action.

Bursts of oscillatory synchronization in the theta range (4–8 Hz) over midfrontal cortex have been proposed as an electrophysiological signature of conflict processing. Recently, we and others have observed this signal also when participants successfully overcame motivational conflict between task-appropriate and task-inappropriate, bias-triggered actions (Cavanagh et al. 2013; Swart et al. 2018; Csifcsák et al. 2019). However, the downstream mechanisms by which midfrontal signals prevent the behavioral expression of motivational biases, putatively driven by the striatum, remain elusive. Previous research has focused on tasks with two active responses (e.g., the Stroop task), where midfrontal theta has been suggested to activate the subthalamic nucleus, which raises the threshold of striatal input needed to elicit an action and thus prevents impulsive actions (Zavala et al. 2014; Frank et al. 2015; Aron et al. 2016; Herz et al. 2016). In contrast, in our task, not only reward-triggered action but also punishment-triggered inhibition needs to be overcome, which might be achieved by a direct attenuation of the cortical inputs into the striatum implemented via recurrent fronto-striatal loops (Alexander et al. 1986; Mink 1996; Gurney et al. 2001). In the current study, we tested the hypothesis that midfrontal theta power is associated with an attenuation of subcortical signals that encode motivational biases when those need to be suppressed.

To test this hypothesis, participants performed a Motivational Go/NoGo learning task known to elicit motivational biases in humans (Swart et al. 2017, 2018) while simultaneously recording fMRI and scalp EEG. The temporal precision of EEG allowed us to separate cue-induced conflict signals from later, response-locked signals. We tested the specific hypotheses that 1) striatal BOLD encodes cue valence, 2) this valence signaling is attenuated when participants successfully overcome motivational biases, and 3) valence signal attenuation correlates with midfrontal theta power. Specifically, using BOLD signal to predict EEG power at different time points allowed us to separate regions involved in (early) valence cue processing versus (later) response biases.

## Materials and Methods

### Participants

Thirty-six participants (*M*_age_ = 23.6, age range 19–32; 25 women, all right-handed) performed the motivational Go/NoGo learning task while simultaneous EEG and fMRI were recorded. Sample size was based on previous EEG studies (Cavanagh et al. 2013; Swart et al. 2018) accounting for potential dropout. The study was approved by the local ethics committee (CMO2014/288; Commissie Mensgebonden Onderzoek Arnhem-Nijmegen). All participants provided written informed consent. Exclusion criteria comprised claustrophobia, allergy to gels used for EEG electrode application, hearing aids, impaired vision, colorblindness, history of neurological or psychiatric diseases (including heavy concussions and brain surgery), epilepsy, metal parts in the body, or heart problems.

Participants attended a single 3-h recording session and were compensated for participation (€30). Additionally, they received a performance-dependent bonus (range €0–5, *M*_bonus_ = €1.28, *SD*_bonus_ = 1.54). The reported behavioral and EEG results are based on all 36 participants. For two participants, fMRI co-registration failed due to excessive orbitofrontal distortion in the T1 image; thus, fMRI results are based on 34 participants (*M*_age_ = 23.5, age range 19–32; 25 women). fMRI-informed EEG results are based on 29 participants (*M*_age_ = 23.4, 21 women): Apart from the two participants for whom co-registration failed, we excluded four further participants who exhibited strong head motion (i.e., at least five volumes with relative displacement larger than the voxel size of 2 mm). These four participants also exhibited stronger overall head motion (i.e., mean relative displacement across all volumes), *M* = 0.213, *SD* = 0.084, compared with the other participants, *M* = 0.088, *SD* = 0.040. Head motion is a particular problem in the EEG-fMRI combined analysis, as it will lead to high and spatially uniform correlations between fMRI and EEG data. Indeed, for these participants, regression weights for all regressors were an order of magnitude larger than for the other participants and largely uniform across time and frequency. We repeated behavioral, EEG, and fMRI results for the subgroup of these 29 participants in Supplementary Material 1; conclusions were identical unless mentioned otherwise in the main text.

### Motivational Go/NoGo Learning Task

**Figure 1:**
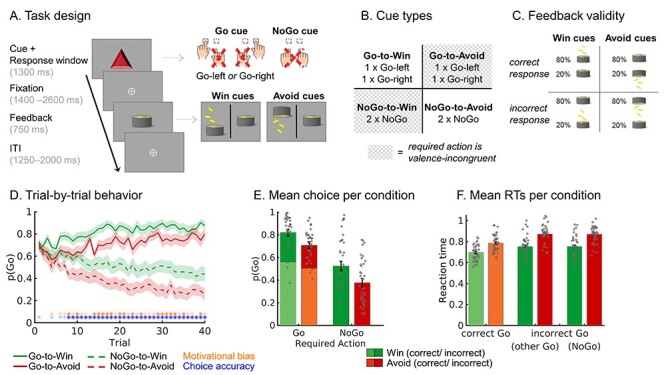
Motivational Go/NoGo learning task and performance. Motivational Go/NoGo learning task design and performance. (*A*) On each trial, a Win or Avoid cue appears; valence of the cue is not signaled but should be learned. Participants should respond during cue presentation. Response-dependent feedback follows after a jittered interval. Compared with a previous study with the same task (Swart et al. 2018), fixations between cue and feedback were jittered and 700–1900 ms longer and ITIs were 250 ms longer to allow us to disentangle cue- and outcome-related activity in the fMRI signal. Each cue has only one correct action (Go-left, Go-right, or NoGo), which is followed by the desired outcome 80% of the time. For Win cues, actions can lead to rewards or neutral outcomes; for Avoid cues, actions can lead to neutral outcomes or punishments. (*B*) There are eight different cues, orthogonalizing cue valence (Win vs. Avoid) and required action (Go vs. NoGo). The motivationally incongruent cues, for which the motivational action tendencies are incongruent with the instrumental requirements, are highlighted in gray. (*C*) Feedback is probabilistic: Correct actions to Win cues lead to rewards in 80% of cases, but neutral outcomes in 20% of cases. For Avoid cues, correct actions lead to neutral outcomes in 80% of cases, but punishments in 20% of cases. For incorrect actions, these probabilities are reversed. Rewards and punishments are depicted by money falling into/out of a can. (*D*) Trial-by-trial proportion of Go actions (±SEM) for Go cues (solid lines) and NoGo cues (dashed lines). Shadows indicate standard errors for per-condition-per-participant means across participants using the Cousineau–Morey method (Morey 2008). The motivational bias is defined as the tendency to make more Go actions to Win than Avoid cues (i.e., green lines are above red lines). Additionally, participants clearly learn whether to make Go actions or not (solid lines go up; dashed lines go down). Orange asterisks below indicate trial-by-trial significance of motivational bias; blue asterisks indicate performance accuracy above chance (i.e., correct GoLeft, GoRight, or NoGo response) (light color: *P* < 0.05 uncorrected; dark color: *P* < 0.0013; Bonferroni corrected for number of trials). (*E*) Mean (±SEM) proportion Go actions per cue condition (points are individual participants’ means). Proportion Go actions is higher for Go than NoGo cues, indicative of task learning, and higher for Win than Avoid cues, reflecting the influence of motivational biases on behavior. (*F*) Mean (±SEM) reaction times for correct and incorrect Go actions, the latter split up in whether the other Go response or the NoGo response would have been correct (points are individual participants’ means). Participants respond faster on correct than on incorrect Go actions and faster to Win than Avoid cues, reflecting the influence of motivational biases on behavior.

Participants performed the motivational Go/NoGo learning task as detailed in (Swart et al. 2018) with trial timings adjusted to the fMRI acquisition (Fig. 1). The task was programmed in MATLAB 2014b (The MathWorks)/Psychtoolbox-3.0.13. Each trial started with the presentation of one of eight cues (a colored geometric shape) in the center of the display (Fig. 1*A*). This cue determined the outcome valence (Win reward/Avoid punishment) and which action (Left Go/Right Go/NoGo) was required for the desired outcome (Win reward/Avoid punishment). Participants had to learn both the valence of the cue and the required action from trial-and-error (Fig. 1*B*). For Win cues, participants should aim to win a reward and avoid neutral outcomes, while for Avoid cues, they should aim to achieve neutral outcomes and avoid punishments. Participants could respond by pressing a left button (Left Go), right button (Right Go), or choose not to press (NoGo) while the cue was on screen for 1300 ms. After a pseudorandomly jittered fixation period of 1400–2600 ms, the outcome was presented for 750 ms in the form of money falling into a can (reward), money falling out of a can (loss/punishment), or just the can (neutral outcome). Outcomes were probabilistic so that the optimal action led to the desired outcome in only 80% of trials, while suboptimal actions led to desired outcomes in 20% of trials (Fig. 1*C*). Each trial ended with a pseudorandomly jittered intertrial interval of 1250–2000 ms, resulting in an overall trial length of 4700–6650 ms. Analyses of learning- and outcome-based activity in EEG and fMRI will be reported in a separate publication.

Participants received two button boxes in the scanner, one for each hand, and were instructed to use only one of the four keys on each button box. When participants accidentally pressed one of the three other buttons, the text “invalid response” appeared instead of an outcome. For analysis purposes, such invalid button presses were recoded into the valid button press of the respective hand, assuming that participants aimed for the correct key of the respective button box.

Before the actual task, participants underwent a practice session in which they were familiarized first with each condition separately (using practice stimuli) and then practiced all conditions together, using different cues from the actual experiment. They were informed about the probabilistic nature of feedback and that each cue features one optimal action. The actual task comprised 320 trials, split into three blocks of ~10 min with short breaks between blocks. Participants performed the task twice, with a different set of cues, yielding 640 trials in total. Introducing a new set of cues allowed us to prevent ceiling effects in performance and investigate continuous learning throughout the task.

### Behavioral Data Analysis

For behavioral analyses, all trials with RTs before 1.3 s (i.e., cue offset) were treated as Go responses (in line with fMRI and EEG analyses). Button presses were treated as Go responses irrespective of whether the correct button or an incorrect button was pressed. We recoded 43 trials with invalid button presses (i.e., pressing a button that was not instructed as response button) into the correct response button of the respective hand (0.19% of trials, max. 14/640 per participant). For analyses of RTs, 12 trials with RTs smaller than 200 ms (0.08% of trials, max. 5/640 per participant) were excluded as such responses were unlikely to follow from cue-based action selection. Furthermore, 980 trials with RTs larger than 1300 ms (i.e., after cue offset, which was the instructed response time limit; 6.5% of trials, max. 139/640 per participant) were excluded from RT analyses as it was unclear whether such button presses were still intended as responses to the cue or were mere “action slips.” Results did not qualitatively change when including these trials.

We used mixed effects logistic regression (lme4 package in R) to analyze participants’ behavioral responses (Go vs. NoGo). We assessed a main effect of required action (Go/NoGo), reflecting whether participants learned the task (i.e., showed more Go response to Go than NoGo cues), a main effect of cue valence (Win/Avoid), reflecting the motivational bias (i.e., more Go response to Win than Avoid cues), and the interaction between valence and required action. The model written in Wilkinson notation was

\begin{align*} & response \sim cueValence^{\ast} \ requiredAction \nonumber\\ & \quad + (cueValence^{\ast} \ requiredAction|participant), \end{align*}

All variables were treated as factors, with sum-to-zero coding. To achieve a maximal random effects structure (Barr et al. 2013), we added random intercepts and random slopes of all three predictors for each participant and further allowed for random correlations between all predictors. The same predictors and random effects structure were used to analyze RTs, for which linear regression was used. *P*-values were computed using likelihood ratio tests (package afex in R). *P*-values smaller than *α* = 0.05 were considered statistically significant. In all plots, whiskers indicate standard errors, which were computed across participants based on the per-condition-per-participant means using the Cousineau-Morey method (Morey 2008).

### fMRI Data Acquisition

MRI data were acquired on a 3 T Siemens Magnetom Prisma fit MRI scanner. In the scanner, participants’ heads were stabilized with foam pillows, and a strip of adhesive tape was applied to participants’ forehead to provide motion feedback and minimize head movement (Krause et al. 2019). After two localizer scans to position slices, functional images were collected using a whole-brain *T*_2_^*^-weighted sequence (68 axial-oblique slices, TR = 1400 ms, TE = 32 ms, voxel size 2.0 mm isotropic, interslice gap 0 mm, interleaved multiband slice acquisition with acceleration factor 4, FOV 210 mm, flip angle 75°, A/P phase encoding direction) and a 64-channel head coil. This sequence yielded a short TR at high spatial resolution, which allowed us to disentangle BOLD signal related to cue and outcome presentation. The sequence parameters were piloted to find a sequence with minimal signal loss in the striatum. The first seven volumes of each run were automatically discarded.

After task completion, when the EEG cap was removed, an anatomical image was collected using a *T*_1_-weighted MP-RAGE sequence (192 sagittal slices per slab, GRAPPA acceleration factor = 2, TI = 1100 ms, TR = 2300 ms, TE = 3.03 ms, FOV 256 mm, voxel size 1.0 mm isotropic, flip angle 8°) for registration and a gradient fieldmap (GRE; TR = 614 ms, TE1 = 4.92 ms, voxel size 2.4 mm isotropic, flip angle 60°) for distortion correction. For one participant, no fieldmap was collected due to time constraints. At the end of each session, an additional diffusion tensor imaging (DTI) data collection took place; results will be reported elsewhere.

### fMRI Preprocessing

fMRI data for each of the six blocks per participants were preprocessed using FSL 6.0.0 (Smith et al. 2004). Functional images were cleaned for nonbrain tissue (BET; Smith 2002), segmented, motion-corrected (MC-FLIRT; Jenkinson et al. 2002), and smoothed (FWHM 3 mm). Fieldmaps were used for B0 unwarping and distortion correction in orbitofrontal areas. We used ICA-AROMA (Pruim et al. 2015) to automatically detect and reject independent components in the data that were associated with head motion (nonaggressive denoising option).

To prevent empty regressors on a block level, we concatenated blocks and performed a single first-level GLM per participant. For this purpose, we registered the volumes of all blocks to the middle image (the default registration option in FSL) of the first block of each participant (using MCFLIRT) and then merged files. The first and last 20 s of each block did not contain any trial events, such that, when modeling trial events, no carryover effects from one block to another could occur.

After concatenation and co-registration of the EPI, we performed high-pass filtering with a cutoff of 100 s, and prewhitening. We then computed the co-registration matrices of EPI images to high-resolution anatomical images (linearly with FLIRT using Boundary-Based Registration) and to MNI152 2 mm isotropic standard space (nonlinearly with FNIRT using 12 DOF and 10 mm warp resolution; Andersson et al. 2007).

### ROI Selection

We used masks of selected ROIs for three purposes: fMRI GLMs with small volume correction, BOLD-RT correlations, and fMRI-informed EEG analyses. For GLMs with small-volume correction, we used anatomical masks of striatum and ACC/pre-SMA based on our a priori hypothesis and previous literature. Anatomical masks were based on the Harvard-Oxford atlas, thresholded at 10%. For BOLD-RT correlations and fMRI-informed EEG analyses, we used conjunctions of anatomical masks and relevant functional contrasts. These are follow-up analyses to results observed in the fMRI GLMs and test independent hypotheses. The well-established rationale for using these conjunctive constraints (O’Reilly et al. 2012) is that the anatomical constraints ensure that all voxels are from the same anatomical region, while the functional constraints ensure that voxels reflect the signal of interest. These masks were obtained by thresholding the z-map of the relevant functional contrast at z > 3.1 (cluster-forming threshold) and then combining it with the anatomical mask using the logical AND operation.

We obtained the following anatomical masks by combining submasks of the Harvard-Oxford atlas: striatum (bilateral caudate, putamen, and nucleus accumbens), midfrontal cortex (anterior division of the cingulate gyrus and juxtapositional lobule cortex—formerly known as supplementary motor cortex), ACC (anterior division of the cingulate gyrus), left and right motor cortex (precentral and postcentral gyrus), and vmPFC (frontal pole, frontal medial cortex, and paracingulate gyrus).

Masks were either used in the group-level GLM for small-volume correction or back-transformed to participants’ native space to extract either parameter estimates from the GLM or the raw BOLD signal time series. All masks are displayed in Supplementary Material 2.

### fMRI Analysis

We fitted a first-level GLM to the data of each participant using the fixed-effects model in FSL FEAT. The four task regressors of interest were the four conditions resulting from crossing cue valence (Win/Avoid) and performed action (Go/NoGo irrespective of Left vs. Right Go), all modeled at cue onset. We also included five regressors of no interest: two at cue onset, namely response side (Go left = +1, Go right = −1, NoGo = 0) and errors (i.e., participants chose the incorrect action), and three at outcome onset to control for outcome-related activity, namely outcome onset (intercept of 1 for every outcome), outcome valence (reward = +1, punishment = −1, neutral = 0), and invalid trials (invalid buttons pressed and thus not feedback given). We further added the following nuisance regressors (separate regressors for each block): intercept, six realignment parameters from motion correction, mean cerebrospinal fluid (CSF) signal, mean out-of-brain (OOB) signal, and separate spike regressors to model out each volume on which relative scan-to-scan displacement was more than 2 mm (occurred in 10 participants; in those participants: *M* = 7.40, range 1–29). Task regressors were convolved with a double-gamma hemodynamic response function (HRF) and high-pass filtered at 100 s. The full model is also displayed in Supplementary Material 3.

We hypothesized that a main effect of valence in the striatum would be attenuated under motivational conflict. Thus, the positive BOLD response to a Win cue would be reduced when having to make a NoGo response for Win, and the negative BOLD response to an Avoid cue would be less negative for a Go response for Avoid (Fig. 3*A*). This hypothesized conflict-mediated attenuation would thus manifest itself as a (weaker) main effect of action in the presence of a (stronger) main effect of valence. Taken together, we predicted that striatal BOLD signal would exhibit both a main effect of valence and a main effect of action. Indeed, previous literature using a simpler version of this task (Guitart-Masip et al. 2011; Guitart-Masip, Chowdhury, et al. 2012; Guitart-Masip, Huys, et al. 2012) has reported a main effect of action in the striatum. In addition to testing the main effects of valence and action, we also computed a congruency contrast, reflecting motivational conflict. Finally, left versus right Go responses were contrasted to identify lateralized motor cortex activation as a quality control check. Regressors were specified across both correct and incorrect trials; we report results for regressors on the correct trials only (in line with our EEG analysis approach) in Supplementary Material 3.

First-level participant-specific contrasts were fitted in native space. Co-registration and re-slicing were then applied on each participant’s contrast maps, which were combined at the group level (using FSL FLAME for mixed effects models; Woolrich et al. 2004, 2009) with a cluster-forming threshold of *z* > 3.1 and cluster-level error control at *α* < 0.05 (i.e., two one-sided tests with *α* < 0.025).

Given our specific hypotheses about the striatum and midfrontal cortex, we additionally tested contrasts using a small-volume correction: For the Valence and Action contrasts, we used an anatomical mask of the striatum, and for the Congruency contrast (i.e., NoGo2Win and Go2Avoid minus Go2Win and NoGo2Avoid), we used a mask of the entire midfrontal cortex (ACC and pre-SMA). While theoretical models predict differences in BOLD signal in ACC, empirical fMRI studies have actually found correlates in more dorsal regions, specifically pre-SMA (Botvinick et al. 2004). Indeed, source reconstruction of midfrontal theta suggested a rather superficial source in pre-SMA (Cohen and Ridderinkhof 2013) and a simultaneous EEG-fMRI study found choice conflict related to pre-SMA BOLD (Frank et al. 2015). Thus, the anatomical mask comprised the entire midfrontal cortex.

### BOLD–Behavior Correlations

To assess whether regions that encoded cue valence, that is, information driving motivational biases, predicted reaction times, we performed regression analyses of the trial-by-trial BOLD signal in relevant regions on RTs (for trials where participants made a Go response). The main regions we found to encode cue valence were vmPFC (positively) and ACC (negatively). We computed conjunctions of anatomical vmPFC and ACC masks with the cue valence contrast and back-transformed the resultant masks to each participant’s native space. We then extracted the first eigenvariate of the signal in both ROIs, returning one summary measure of BOLD signal in that ROI per volume. The volume-by-volume signal in each ROI was then high-pass filtered at 128 s, and nuisance regressors (6 realignment parameters, CSF, OOB, single volumes with strong motion, same as in the fMRI GLM) were regressed out. Afterwards, the signal was upsampled by factor 10, epoched into trials of 8-s duration (Hauser et al. 2015), and a separate HRF was fitted for each trial (i.e., 57 upsampled datapoints).

We then tested whether BOLD signal in those ROIs correlated with reaction times on a trial-by-trial basis. In order to account for overall differences between trials with Win cues and trials with Avoid cues, we standardized reaction times and BOLD signal separately for Win trials and Avoid trials within each participant such that differences between cue valence conditions were removed. We computed correlations between trial-by-trial reaction times and BOLD signal HRF amplitude for each participant, applied Fisher *z-*transformations to correlations to make them normally distributed, and then tested with a one-sample *t*-test whether correlations were significantly different from zero at a group level.

### E‌EG Data Acquisition and Preprocessing

EEG data were acquired using 64 channels (BrainCap-MR-3-0 64Ch-Standard; Easycap GmbH; international 10–20 layout, reference electrode at FCz) at a sampling rate of a 1000 Hz using MRI-compatible EEG amplifiers (BrainAmp MR plus; Brain Products GmbH). Additional channels for electrocardiogram, heart rate, and respiration were used to record for MR artifact correction. Recordings were performed with Brain Vision Recorder Software (Brain Products). EEG amplifiers were placed behind the scanner, and cables were attached to the cap once participants were positioned in the scanner. Cables were fixated with sand-filled pillows to reduce artifacts induced through cable movement in the magnetic field. During functional scans, the MR scanner helium pump was switched off to reduce EEG artifacts. A Polhemus FASTRAK device was used to record the exact location of each EEG electrode on the participant’s head relative to three fiducial points. For four participants, no Polhemus data were recorded due to time constraints and technical errors; for these participants, the average channel positions of the remaining 32 participants were used.

EEG data were cleaned from MR scanner and cardioballistic artifacts using BrainVisionAnalyzer (Allen et al. 2000). Preprocessing was performed in Fieldtrip (Oostenveld et al. 2011) in MATLAB 2017b by rejecting channels with high residual MR noise (mean 4.8 channels per participant, range 0–13), epoching trials (−1750–2800 ms relative to cue onset, total duration of 4550 ms), re-referencing the channels to their grand average and recovering the reference as channel FCz, band-pass filtering the data in the 0.5–15 Hz range using a two-pass fourth-order Butterworth IIR filter (Fieldtrip default), and finally linear baseline correction based on the 200 ms prior to cue onset. The low-pass filter cutoff of 15 Hz allowed us to dissociate theta from its adjacent bands while filtering out residual high-frequency MR noise. We used ICAs to visually identify and reject independent components related to eye blinks, saccades, head motion, and residual MR artifacts (mean 12.94 components per participant, range 8–19), and afterwards manually rejected trials that were still contaminated by noise (mean 29.6 trials per participant, range 2–112). Finally, we computed a Laplacian filter with the spherical spline method to remove global noise (using the exact electrode positions obtained with the Polhemus FASTRAK), which we also used to interpolate previously rejected channels. This filter attenuates more global signals (deep sources) and noise (heart-beat and muscle artifacts) while accentuating more local effects (superficial sources).

For response-locked analyses, we re-epoched Go actions trials, time-locked to the time of response (RT). For NoGo response trials, we re-epoched the data time-locked to the average RTs (for each participant) of Go actions for that cue valence, as a proxy for “latent RTs” on these trials.

### E‌EG TF Decomposition

Time–frequency decomposition was performed using Hanning tapers between 1 and 15 Hz in steps of 1 Hz, every 25 ms with 400-ms time windows. We first zero-padded trials to a length of 8 s. and then performed time–frequency decomposition in steps of 1 Hz by multiplying the Fourier transform of the trial with the Fourier transform of a Hanning taper of 400-ms width, centered around the time point of interest. This procedure results in an effective resolution of 2.5 Hz (Rayleigh frequency), interpolated in 1-Hz steps, which is more robust to the exact choice of frequency bins. Given that all preprocessing was performed on data epoched into trials, we aimed to exclude the possibility of slow drifts in power over the time course of the experiment. We thus performed baseline correction by fitting a linear model across trials for each channel/frequency combination. This model included trial number as a regressor and the average power in the last 50 ms before cue onset as outcome. The power predicted by this model was then removed from single-trial data. Note that in absence of any drift, this approach amounts to correcting all trials by the grand-mean across trials per frequency in the selected baseline window per participant. Next, we averaged over trials within each condition spanned by valence (Win/Avoid) and action (Go/NoGo; correct trials only). Finally, power was converted to decibel for all analyses to ensure that data across frequencies, time points, electrodes, and participants were on the same scale.

In line with (Swart et al. 2018), we restricted our analyses to correct trials, that is, trials where required and performed action matched. We assumed that on correct incongruent (Go2Avoid and NoGo2Win) trials, participants successfully detected and resolved conflict, while no such processes were required on congruent (Go2Win and NoGo2Avoid) trials. Although the same processes might be initiated on incorrect trials, though unsuccessfully, these trials are potentially confounded by error-related synchronization in the theta range (Cavanagh, Zambrano-Vazquez, et al. 2012), which makes the interpretation of any effects in the theta range less straightforward. To be consistent with fMRI results, we report results across both correct and incorrect trials in Supplementary Materials 6 and 10.

### E‌EG Data Analysis

All analyses were performed on the average signal of the a priori selected channels Fz, FCz, and Cz based on previous findings (Swart et al. 2018). We performed nonparametric cluster-based permutation tests (Maris and Oostenveld 2007) as implemented in Fieldtrip for the selected electrodes in the theta range (4–8 Hz) during cue presentation (0–1300 ms). This procedure is suited to reject the null hypothesis of exchangeability of two experimental conditions, but not suited to exactly determine when or where differences occur (Sassenhagen and Draschkow 2019). Our interpretations of when and where conditions differed in power are thus based on visual inspection of the signal time courses.

Given our a priori hypothesis of midfrontal theta power reflecting conflict, we performed the test contrasting bias-incongruent than bias-congruent actions to the theta range (4–8 Hz). Furthermore, since visual inspection of the condition-specific time courses of theta power suggested major differences in theta power between Go and NoGo responses, we additionally performed an exploratory test contrasting Go and NoGo responses. Given its exploratory nature, this test was performed on broadband power (1–15 Hz).

### fMRI-Informed EEG Analysis

The sluggish nature of the BOLD signal makes it difficult to determine when exactly different brain regions become active. In contrast, EEG provides much higher temporal resolution. Identifying distinct EEG correlates of the BOLD signal in different regions could thus reveal when these regions become active (Hauser et al. 2015). Furthermore, using the BOLD signal from different regions in a multiple linear regression allows to control for variance that is shared among regions (e.g., changes in global signal; variance due to task regressors) and test which region is the best unique predictor of a certain EEG signal. In such an analysis, any correlation between EEG and BOLD signal from a certain region reflects an association above and beyond those induced by task conditions.

To link BOLD signal from distinct regions to time–frequency power, we applied the same approach as for BOLD-RT correlation analyses and fitted a trial-by-trial HRF to the BOLD signal in selected ROIs. We then used the trial-by-trial HRF amplitudes to predict TF power at each time–frequency–channel bin (Hauser et al. 2015) (building on existing code from https://github.com/tuhauser/TAfT). For BOLD signal extraction, we specified six ROIs using a combination of functional and anatomical constraints based on our fMRI GLM results: vmPFC (valence contrast), ACC (valence and action contrast), left and right motor cortex (response side contrast, which captured lateralized motor activity better than the action contrast), and striatum (action contrast). For ACC, we obtained two separate masks (valence and action contrast), which strongly overlapped. Analyses included only one of those masks at a time; conclusions were identical with either mask. The resultant masks were back-transformed to each participant’s native space and the first eigenvariate of the signal in each ROI was extracted, returning one summary measure of BOLD signal in that ROI per volume. We applied the same high-pass filter, nuisance regression, upsampling procedure, and trial-by-trial HRF estimation as in BOLD-RT correlation analyses. The trial-wise HRF amplitude estimates for each ROI were used as regressors in a multiple linear regression to predict the TF power for each 3D time–frequency–channel bin across trials, resulting in a 3D map of regression weights (*b*-map) for each ROI. In these regressions, we also added behavioral regressors for the main effects of required action, valence, and their interaction as covariates of no interest to account for task-related variance in EEG power. In all analyses, predictors and outcomes were demeaned so that any intercept was zero. Finally, participants’ *b*-maps were Fisher *z-*transformed (which makes the sampling distribution of correlation coefficients approximately normal and allows to combine them across participants).

Finally, to test whether BOLD signal in certain ROIs uniquely predicted variance in TF power, we performed cluster-based one-sample permutation *t*-tests across participants (Hunt et al. 2013). We performed these tests on the mean regression weights of the channels that exhibited condition differences in the EEG-only analyses (FCz and Cz; Fz was dropped because it did not show significant power differences between conditions) in the range of 0–1300 ms (i.e., duration of cue presentation), 1–15 Hz. We first obtained a null distribution of maximal cluster mass statistics from 10 000 permutations. In each permutation, the sign of the *b*-map of a random subset of participants was flipped. Then, a separate *t*-test for each time–frequency bin (bins of 25 ms, 1 Hz) across participants was computed. The resulting *t*-map was thresholded at |*t*| > 2, from which we computed the maximal cluster mass statistic (i.e., sum of all *t*-values) of any cluster (i.e., adjacent voxels above threshold). We next computed a *t*-map for the real data, from which we identified the cluster with largest cluster mass statistic. The corresponding *P-*value was computed as the number of permutations with larger maximal cluster mass than the maximal cluster mass of the real data and considered significant for a *P*-value of <0.05.

## Results

Thirty-six healthy participants performed a motivational Go/NoGo learning task. In this task, they needed to learn by trial and error which response (left Go/right Go/NoGo) to make in order to gain rewards (“Win” cues) or avoid losses (“Avoid” cues); see Figure 1. We simultaneously measured EEG and fMRI while participants performed this task.

### Task Performance

Participants successfully learned the task, as they performed significantly more Go actions to Go cues than NoGo cues (Required action: *χ*^2^(1) = 32.01, *P* < 0.001; Fig. 1*D*,*E*). Furthermore, participants showed a motivational bias, as they performed more Go actions for Win cues than Avoid cues (Valence: *χ*^2^(1) = 23.70, *P* < 0.001). The interaction of Required action × Valence was not significant (*χ*^2^(1) = 0.20, *P* = 0.658), suggesting that motivational biases occurred similarly for Go and NoGo cues.

When making a (Go) action, participants responded faster to Go cues (averaged over correct and incorrect responses) than to NoGo cues (where responses were by definition incorrect) (Required action: *χ*^2^(1) = 25.64, *P* < 0.001). Furthermore, a motivational bias was present also in RTs, with significantly faster actions to Win than Avoid cues (Valence (*χ*^2^(1) = 44.58, *P* < 0.001). Again, the interaction was not significant (*χ*^2^(1) = 1.51, *P* = 0.219); see Fig. 1*F*).

### fMRI

#### Valence

**Figure 2:**
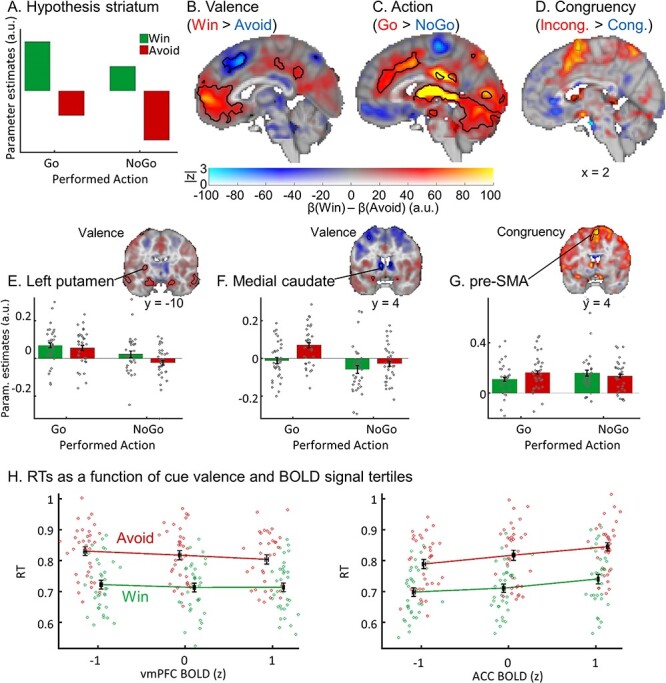
fMRI results. BOLD signal as a function of cue valence, performed action, and congruency. (*A*) We hypothesized striatal BOLD to encode cue valence (main effect of valence), with an attenuation of this valence signal when actions incongruent to the bias-triggered actions were performed (main effect of action). (*B*) BOLD signal was significantly higher for Win compared with Avoid cues in ventromedial prefrontal cortex (vmPFC; whole-brain corrected) and left putamen (small-volume corrected), but higher for Avoid compared with Win cues in ACC and medial caudate (small-volume corrected). (*C*) BOLD signal was significantly higher for Go compared with NoGo responses in the entire striatum as well as ACC, thalamus, and cerebellum (all whole-brain corrected). (*D*) BOLD signal was significantly higher for bias-incongruent actions than bias-congruent actions in pre-SMA (small-volume corrected). (*B*–*D*) BOLD effects displayed using a dual-coding visualization with color indicating the parameter estimates and opacity the associated z-statistics. Contours indicate statistically significant clusters (*P* < 0.05), either small-volume corrected (striatal and SMA contours explicitly linked to a bar plot) or whole-brain corrected (all other contours). (*E*–*G*) Mean beta weights per task condition (*x*-axis) per participant (individual gray dots) in significant clusters in left putamen, medial caudate, and pre-SMA (significant in small-volume correction). (*E*) Left posterior putamen encoded valence positively (higher BOLD for Win than Avoid cues) but was dominated by an encoding of the performed action (higher BOLD for Go than NoGo responses). (*F*) Medial caudate encoded valence negatively (higher BOLD for Avoid than Win cues), again predominantly showing a main effect of action. (*G*) BOLD signal in pre-SMA was higher for bias-incongruent than bias-congruent actions (small-volume corrected). (*H*) Reaction times (RTs) as a function of cue valence and BOLD signal tertiles (z-standardized) per participant (individual dots; *x*-location relative to all other participants). RTs were significantly predicted by BOLD signal in vmPFC (positively) as well as by BOLD signal in ACC and striatum (negatively). BOLD-RT correlations were independent of cue valence. Lines connect the means of RT tertiles. Error bars (±SEM) for both BOLD (vertically) and RTs (horizontally) are very narrow.

We hypothesized higher BOLD signal in the striatum for Win compared with Avoid cues (Fig. 2*A*). There were no significant clusters in the striatum in a whole-brain corrected analysis. When restricting our analyses to an anatomical mask of the striatum, there were two significant clusters (for a complete list of all significant clusters and *P-*values, see Supplementary Material 4): BOLD signal in left posterior putamen was significantly higher for Win than Avoid cues (Fig. 2*E*; no longer significant when excluding the five participants that were excluded from the final EEG-fMRI analysis; Supplementary Material 1). In contrast, BOLD signal in the bilateral medial caudate nucleus was, surprisingly, higher for Avoid than Win cues (Fig. 2*F*). While the effect in left putamen appeared robust over the time course of the task, the effect in medial caudate was strongest at the beginning of the task but then disappeared toward the end of the task (see Supplementary Material 5). At a whole-brain level cluster correction, the largest cluster of higher BOLD signal for Win than Avoid cues was observed in the ventromedial prefrontal cortex (vmPFC). Positive valence coding was also observed in bilateral dorsolateral prefrontal cortex (dlPFC), bilateral ventrolateral prefrontal cortex (vlPFC), posterior cingulate cortex (PCC), bilateral amygdala, and bilateral hippocampus (Fig. 2*A*). Conversely, BOLD was higher for Avoid cues in dorsal ACC and bilateral insula. Repeating these analyses on only the correct trials yielded results identical to the whole-brain analyses, while effects on posterior putamen and medial caudate with small-volume correction were not significant anymore (see Supplementary Material 6).

#### RTs

We reasoned that any region translating cue valence into motivational biases in behavior should also predict the speed-up of RTs for Win compared with Avoid cues. Our finding that vmPFC and ACC BOLD reflected cue valence is in line with a wealth of previous literature (Haber and Knutson 2010; Bartra et al. 2013), raising the question whether signals from these two regions impact action selection in a way that gives rise to motivational biases. We reasoned that if such signals have a causal effect on behavior, they should predict RT differences, that is, both overall RT differences between Win and Avoid cues, but also RT differences within each valence condition. To assess whether BOLD signal in vmPFC and ACC indeed related to the speed of selected actions, we computed correlations of reaction times with the trial-by-trial deconvolved BOLD signal in the identified vmPFC and ACC clusters. To account for overall differences between Win and Avoid cues, we standardized RTs and BOLD signal separately for Win and Avoid cues, removing the overall difference between both conditions. There was a strong negative association for vmPFC, *t*(33) = −4.11, *P* < 0.001, *d* = −0.71, with higher vmPFC BOLD predicting faster reaction times, and a strong positive association for ACC, *t*(33) = 7.83, *P* < 0.001, *d* = 1.34, with higher ACC BOLD predicting slower reaction times (Fig. 2*H*). These results are consistent with vmPFC and ACC, both signaling cue valence, influencing the speed of downstream actions and thus contributing to motivational biases in reaction times. However, we cannot infer a causal role of these regions from the observed correlation.

#### Action

We further hypothesized that the valence signal in the striatum would be modulated by the congruency between valence and action, such that increased striatal BOLD signal for Win cues would be dampened when (bias-incongruent) NoGo responses were required, while decreased striatal activity for Avoid cues should be elevated when (bias-incongruent) Go actions were required (Fig. 2*A*). This interaction effect between valence and congruency is equivalent to a main effect of action.

Striatal BOLD (bilateral caudate nucleus, putamen, and nucleus accumbens) was indeed significantly higher for Go than NoGo responses (Fig. 2*C*). This effect was absent on the first block but strongly emerged over time (see Supplementary Material 5). However, in absence of a clear effect of valence on striatal BOLD, this main effect of action might not reflect an attenuation of valence signaling. Rather, the striatum appears to predominantly encode action itself—even in the left posterior putamen, which significantly encoded valence (Fig. 2*E*). This finding replicates previous studies using a different version of the Motivational Go/NoGo task (Guitart-Masip et al. 2011; Guitart-Masip, Chowdhury, et al. 2012; Guitart-Masip, Huys, et al. 2012). Other regions that responded more strongly to Go versus NoGo responses included the ACC, thalamus, and bilateral cerebellum. For a complete list of significant clusters, see Supplementary Material 4. Repeating analyses on correct trials only yielded identical results (see Supplementary Material 6).

#### Valence x Action Interaction (Congruency)

Based on prior work, we expected increased BOLD for bias-incongruent compared with bias-congruent actions in midfrontal cortex, the putative cortical source of midfrontal theta oscillations. At a whole-brain cluster level significance correction, there were no clusters in which BOLD signal differed between bias-congruent and -incongruent actions. When restricting the analysis to a mask comprising ACC and pre-SMA, there was a cluster in pre-SMA (Fig. 2*D*,*G*; not significant when excluding the five more participants excluded in the final EEG-fMRI analysis; see Supplementary Material 1; not significant in the GLM on correct trials only, see Supplementary Material 6). This finding is in line with source reconstruction studies of midfrontal theta (Cohen and Ridderinkhof 2013) and EEG-fMRI findings observing choice conflict-related activity in pre-SMA (Frank et al. 2015). The effect of conflict in pre-SMA was robust over time (see Supplementary Material 5).

### E‌EG

#### Action x Valence Interaction (Congruency)

**Figure 3:**
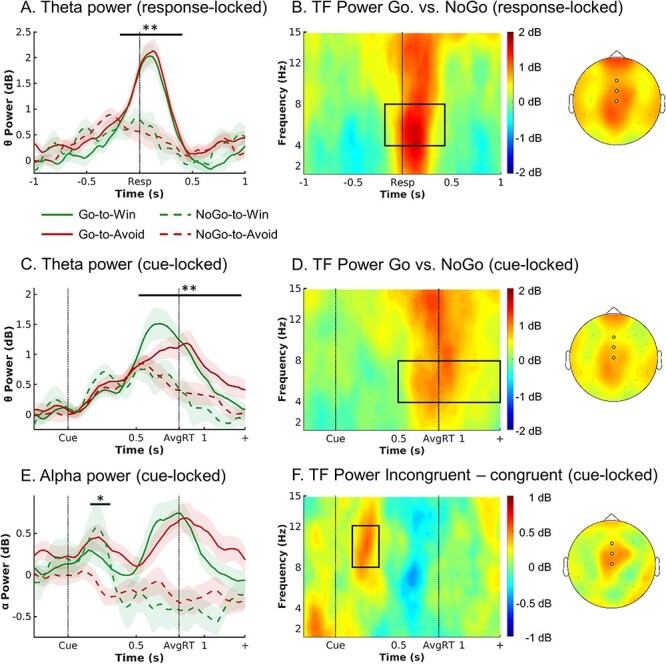
EEG results. EEG time–frequency power as a function of cue valence and action. (*A*) Response-locked within trial time course of average theta power (4–8 Hz) over midfrontal electrodes (Fz/FCz/Cz) per cue condition (correct trials only). Theta increased in all conditions relative to precue levels, but to a higher level for Go than NoGo trials. There were no differences in theta peak height or latency between Go2Win and Go2Avoid trials. (*B*) Left: Response-locked time–frequency power over midfrontal electrodes for Go minus NoGo trials. Go trials featured higher broadband TF power than NoGo trials. The broadband power increase for Go compared with NoGo trials is strongest in the theta range. Right: Topoplot for Go minus NoGo trials. The difference is strongest at Cz and FCz electrodes. (*C* and *D*) Cue-locked within trial time course and time–frequency power. Theta increased in all conditions relative to precue levels, but to a higher level for Go than NoGo trials, with earlier peaks for Go2Win than Go2Avoid trials. (*E*) Trial time course of average alpha power (8–13 Hz) over midfrontal electrodes per cue condition (correct trials only; cue-locked). Alpha power transiently increases for both incongruent conditions in an early time window (around 175–325 ms). (*F*) Left: Time–frequency plot displaying that the transient power increase was focused on the alpha band (8–13 Hz), leaking into the upper theta band. Right: Topoplot of alpha power displaying that this incongruency effect was restricted to midfrontal electrodes (highlighted by white disks). ^*^P < 0.05. ^**^P < 0.01. Shaded error bars indicate (±SEM). Box in TF plots indicates the time–frequency window where *t*-values > 2.

We used a nonparametric, cluster-based permutation test to test whether time–frequency (TF) power in the theta band (4–8 Hz) was significantly higher on motivational incongruent (Go2Avoid, NoGo2Win) than congruent (Go2Win, NoGo2Avoid) trials over midfrontal channels (Fz, FCz, Cz). We indeed found that theta power was higher on incongruent than congruent trials (*P* = 0.023), most strongly around 175–325 ms after cue onset. However, this difference occurred markedly earlier than in our previous study (450–650 ms) (Swart et al. 2018) and visual inspection of the time–frequency plot showed that the peak of this cluster was located rather in the alpha band (8–12 Hz), leaking into the upper theta range (Fig. 3*E*,*F*), and restricted to an early, transient increase in alpha power over midfrontal channels. This congruency effect was not present in evoked activity (ERPs; see Supplementary Material 7) and indeed remained unaltered after the subtraction of evoked activity (see Supplementary Material 8). Furthermore, this change in alpha power occurred selectively on incongruent trials on which participants made a correct response, rather than nonspecifically on all incongruent or on all correct trials (see Supplementary Material 9). In other words, power increased selectively when biases were successfully suppressed (Swart et al. 2018). In sum, we found an electrophysiological correlate of conflict, which was however different in time and frequency range from our previous finding (Swart et al. 2018).

#### Action and Valence Main Effects

We observed a modulation of time–frequency power by valence–action congruency in the alpha range shortly after cue onset (around 175–325 ms). We next performed permutation tests to explore whether time–frequency power was further modulated at any later time point by the individual task factors rather than their interaction (i.e., action or valence). Broadband power (1–15 Hz) was significantly higher on trials with Go actions than NoGo responses (cue-locked: *P* = 0.002; response-locked: *P* = 0.002): This difference between Go and NoGo responses occurred as a broadband signal from 1–15 Hz, but peaked in the beta band (cue-locked) and theta band (response-locked; Fig. 3*B*,*D*). The topographies exhibited a bimodal distribution with peaks both at frontopolar (FPz) and central (FCz, Cz, CPz) electrodes (Fig. 3*B*,*D*). As visual inspection of Fig. 3*C* shows, theta power increased in all conditions until 500 ms post cue onset and then bifurcated depending on the action: For NoGo responses, power decreased, while for Go actions, power kept rising and peaked at the time of the response. This resulted in higher broadband power for Go versus NoGo responses for about 550–1300 ms after cue onset (see Fig. 3*C*,*D*; around −200–400 ms when response-locked, see Fig. 3*A*,*B*; same held in analyses across both correct and incorrect trials, see Supplementary Material 10). When looking at the cue-locked signal, the signal peaked earlier and higher for Go actions to Win than to Avoid cues, in line with faster reaction times on Go2Win than Go2Avoid trials. When testing for differences in broadband power between Win cues and Avoid cues, broadband power was indeed higher for Avoid than Win cues around 825–1300 ms cue-locked (*P* = 0.002). When comparing power time courses for Go responses to Win and to Avoid cues selectively in the theta range, theta power was higher for Win than Avoid cues around 550–700 ms, *P* = 0.028, but then higher for Avoid than Win cues around 925–1300 ms, *P* = 0.002. This difference in latency and peak height of the ramping signal was not present in the response-locked signal, and the respective test of Win versus Avoid cues not significant (*P* = 0.110; Fig. 3*A* and Supplementary Material 11).

The occurrence of this signal close to response execution across a broadband frequency range raised the question whether it reflects 1) a decision process (incorporating decision parameters like cue valence and action values) or rather 2) a generic motor signal occurring for any manual response, or even 3) a signal artifact of head motion in the scanner. Regarding the latter, a number of control analyses indicated that the theta increase was likely not reducible to a motor artifact: First, results remained unchanged when accounting for fMRI-realignment parameters (reflecting head motion) using a linear regression approach (following Fellner et al. 2016; see Supplementary Material 12), and second, relative to the pretrial baseline, increases were clearly focused on the theta band (see Supplementary Material 13), started already 300 ms after cue onset, and even occurred on NoGo trials (i.e., where no overt response was executed). Furthermore, the theta signal was unlikely to reflect a generic motor response, as it was modulated by task demands: Theta (but not broadband) power was higher for left, nondominant hand compared with the right, dominant hand (in line with reaction time findings; see Supplementary Material 14), and higher for correct than incorrect responses (see Supplementary Material 11). Taken together, we cannot exclude the possibility that motor execution processes or signal artifacts contributed to the observed differences in theta power; however, differences in theta are likely not reducible to such processes and do at least in part reflect preresponse, decision-related processes.

Next, if midfrontal theta power reflects a decision process, we asked whether this signal bore resemblance to evidence accumulation processes described in perceptual decision-making before (Gold and Shadlen 2007; O’Connell et al. 2012). In such processes, a response is elicited once an accumulation signal reaches a certain threshold. This observation was particularly the case for the theta band (Fig. 3*C*) rather than any other band (see alpha band in Fig. 3*E*). Three further tests corroborated this interpretation: First, the peak of the ramping theta signal in the cue-locked data predicted reaction times within participants (see Supplementary Material 11), while differences in peak height and latency were absent in the response-locked data. Thus, faster accumulation of evidence in favor of a “Go” response for “Win” cues could be the driving mechanism for a motivational bias. This change in accumulation rate would then be putatively driven by a neural region encoding cue valence, such as vmPFC or ACC. Second, the “threshold” that theta needed to reach to elicit a response was higher for responses of the left (nondominant) than the right (dominant) hand (in line with reaction times findings; Supplementary Material 14), putatively reflecting response competition in which the dominant hand needs to be overruled by raising response thresholds. Third, peak theta was lower for incorrect than correct responses (Supplementary Material 11), consistent with the idea that incorrect responses might (sometimes) reflect premature responding due to random fluctuations in response thresholds (O’Connell et al. 2012). These three observations are consistent with an interpretation of the ramping theta signal as reflecting an evidence accumulation process for Go-related evidence.

### fMRI-Informed EEG Analysis

Given that we observed action encoding in both BOLD (ACC, striatum) and midfrontal EEG power (theta), we next tested when differences in activity in these regions occurred by correlating the BOLD signal from those regions with midfrontal EEG time–frequency power. We extracted trial-by-trial BOLD signal from clusters encoding valence (vmPFC, ACC), action (striatum, ACC), and response hand (left and right motor cortex) and used these signals in a multiple linear regression to predict midfrontal time–frequency power.

**Figure 4:**
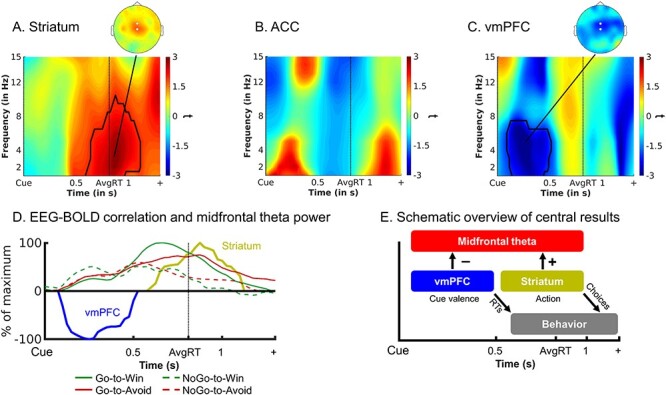
Uniquely explained variance in EEG time-frequency power over midfrontal electrodes (FCz/Cz) by BOLD signal from (*A*) whole striatum, (*B*) ACC, and (*C*) vmPFC. The group-level *t*-maps display the modulation of the EEG time-frequency power by trial-by-trial BOLD signal in the selected ROIs. Striatal (but not ACC) BOLD correlates most strongly with theta/delta power around the time of response. vmPFC BOLD correlates with broadband (peak: theta) power soon after cue onset. Areas surrounded by a black edge indicate clusters of |t| > 2 with *P* < 0.05 (cluster-corrected). Topoplots indicate the topography of the respective cluster. Note that there are no significant clusters in ACC, but given our a priori hypothesis regarding the relation between ACC BOLD and midfrontal theta, for completeness, we include this visualization. (*D*) Time course of vmPFC and striatal BOLD correlations with theta power (*t*-values from clusters above threshold extracted and summed over frequencies), normalized to the peak of the time course of each region, overlaid with theta power for each valence × action condition. vmPFC–theta correlations emerge when theta is still similar for each condition, while striatum–theta correlations emerge when theta rises more strongly for Go than NoGo responses. (*E*) Schematic overview of our main EEG-fMRI results: Both vmPFC and striatum modulate midfrontal theta power (striatum likely indirectly via motor areas, see Supplementary Material 17). BOLD signal in both regions predicts the amplitude of theta power—the vmPFC early and negatively, the striatum late and positively. We speculate that the vmPFC encodes cue valence and sends this information to the striatum, where valence information biases the motivation for active responses in recurrent fronto-striatal loops and thus gives rise for motivational biases in behavioral responses and reaction times.

First, trial-by-trial analyses revealed that striatal BOLD correlated positively with theta power around the time of response, over and above task and condition effects (*P* = 0.037 cluster-corrected; Fig. 4*A*). Conversely, and perhaps surprisingly, there was no such correlation between midfrontal theta power and ACC BOLD (mask based on action contrast: *P* = 0.268 cluster-corrected; mask based on valence contrast: *P* = 0.592). Supplementary analyses yielded correlations between motor cortex BOLD and midfrontal beta power (Jurkiewicz et al. 2006; Ritter et al. 2009), corroborating the overall ability of our approach in detecting well-established BOLD-EEG associations (Supplementary Material 15). Note that because the design matrix included all ROI time series as well as task regressors, these correlations only reflect variance uniquely explained by a specific ROI, over and above task effects in these regressors.

Next, to investigate whether vmPFC BOLD, which encoded cue valence rather than action, contributes to action selection in fronto-striatal circuits, we also assessed an association between midfrontal EEG power and BOLD in the vmPFC. Trial-by-trial deconvolved BOLD signal from the valence cluster in vmPFC correlated negatively with broadband power (*P* = 0.043, cluster-corrected) in an early cluster (around 2–15 Hz, peak in the upper theta range around 6–7 Hz, 0–0.4 s, Fig. 4*C*). Regression of the time-domain EEG voltage on vmPFC BOLD yielded a negative association of vmPFC BOLD with a left frontal P2 component, which however showed a different topography and was unlikely to explain the negative vmPFC–theta correlations over midfrontal electrodes (see Supplementary Material 16).

Finally, we performed complementary EEG-informed fMRI analyses using the trial-by-trial midfrontal alpha and theta signals identified in EEG-only analyses as regressors on top of the task regressors. While fMRI-informed EEG analyses allow to test which region is the best predictor of a certain EEG signal (competing with BOLD signal from other regions), this EEG-informed fMRI analysis allows to assess whether networks of several regions might reflect trial-by-trial changes in power (although none of them might do so uniquely). Alpha power correlated negatively with dlPFC and SMG BOLD, while theta power correlated positively with BOLD signal in bilateral pre-SMA, ACC, motor cortices, operculum, putamen, and cerebellum, corroborating the association between theta and motor regions, including the striatum (see Supplementary Material 17). See Supplementary Material 17 for a methodological discussion on these seemingly contrasting findings.

In sum, the fMRI-informed EEG analyses show that the amplitude of the ramping theta signal at the time of response correlates positively with striatal BOLD signal. In contrast, theta power early after cue onset correlates negatively with vmPFC activity. Finally, ACC activity in the cluster encoding Go versus NoGo responses was not significantly linked to theta power, while other parts of ACC and pre-SMA (and further motor regions) were in fact related to trial-by-trial theta power. Taken together, we observed an early negative correlation of theta with vmPFC BOLD, which encoded cue valence, and a late positive correlation of theta (ramping up to the response) with striatal BOLD, which encoded the selected action.

### Summary of the Main Results

In sum, we observed motivational biases in both choice and reaction time data. Cue valence, which drives these biases, led to differences in BOLD signal in vmPFC (higher for Win cues) and ACC (higher for Avoid cues). These vmPFC and ACC BOLD responses to cue valence also predicted reaction times on a trial-by-trial basis. Striatal BOLD did not respond to cue valence but predominantly reflect the action participants selected (higher for Go than NoGo). Motivational conflict was associated with higher BOLD in midfrontal cortex, though only weakly, and with early transient midfrontal alpha power. In contrast, midfrontal theta power reflected the selected action (higher for Go than NoGo) around the time of responses. Finally, trial-by-trial vmPFC BOLD correlated negatively with theta power early after cue onset, while striatal BOLD correlated positively with theta power around the time of responses.

## Discussion

The main aim of this study was to investigate the interaction of striatal and midfrontal processes during the suppression of motivational biases, using combined EEG-fMRI. We and others have previously found elevated theta power when such biases were successfully suppressed (Cavanagh et al. 2013; Swart et al. 2018). Furthermore, computational models of basal ganglia loops posited the origin of this bias in the striatum (Frank 2005; Collins and Frank 2014). We thus hypothesized that theta power would correlate with attenuated striatal valence coding during bias-incongruent actions. However, we found that both striatal and theta signals were strongly dominated by action per se, rather than by a combination of valence and action, as was predicted for the striatum, or by valence–action congruency, as was predicted for the midfrontal cortex. Most importantly, we found that the time course of theta power exhibited several features of a process accumulating evidence whether to select an active Go response or not. Interestingly, valence-driven vmPFC BOLD signal uniquely predicted variability in midfrontal theta immediately following cue presentation, while action-driven striatal BOLD responses uniquely predicted variability in midfrontal theta around the time of the response. Taken together, these results suggest that striatum and vmPFC may act in concert to evaluate the value of performing an active Go response, that is, the “value of work.”

### Striatal BOLD Reflects Motivation for Action

We found that striatal BOLD signal was strongly dominated by the action participants performed (Go vs. NoGo) rather than cue valence. This finding replicates previous studies using a very similar task (Guitart-Masip et al. 2011; Guitart-Masip, Chowdhury, et al. 2012; Guitart-Masip, Huys, et al. 2012) and highlights the role of the striatum in value-based behavioral activation and invigoration (Taylor and Robbins 1984, 1986; Robbins and Everitt, 1992, 2007; Niv et al. 2007; Salamone and Correa 2012; Howe and Dombeck 2016; Syed et al. 2016; Coddington and Dudman, 2018, 2019; da Silva et al. 2018) yet appears to be at odds with the role of the striatum in reward expectation (Doya 2000; Dayan and Daw 2008; Collins and Frank 2014). A recent theory aimed to reconcile these roles by proposing that striatal (dopamine) signals do not reflect the value of anticipated outcomes per se, but rather the value of performing an action to obtain this outcome, that is, the “value of work” (Berke 2018). Recent empirical work in rodents has indeed shown selective ramping of striatal dopamine signals for active responses approaching a goal state (Hamid et al. 2016, 2021; Syed et al. 2016; Mohebi et al. 2019). In light of these findings, it seems plausible that the striatum evaluates whether there are sufficient incentives to overcome a NoGo default and to instead take action to achieve a valuable goal. Any signal that reflects such an evolving value of Go should ramp over the trial time course and peak when a response is elicited, like an evidence accumulation process. While the BOLD response is too sluggish for capturing such fast within-trial signals, EEG can provide insight.

### Late Midfrontal Theta Power Reflects Striatal Activity

In this study, midfrontal theta power, like striatal BOLD, was modulated by whether participants made an active Go response or not. Theta power bifurcated for Go and NoGo responses, peaking around the time of the response. Striatal BOLD and midfrontal theta signals were strongly linked, such that trial-by-trial fluctuations in striatal (rather than ACC or motor cortex) BOLD was the best predictor of trial-by-trial fluctuations in theta power around the time of response.

The observed link between striatal BOLD and midfrontal theta may seem surprising given that previous EEG source localization studies of conflict-related midfrontal theta power modeled a source in ACC or pre-SMA (Hanslmayr et al. 2008; Cohen and Ridderinkhof 2013), and previous resting-state EEG-fMRI studies reported negative correlations of frontal theta with regions of the default-mode network (Scheeringa et al. 2008, 2009). Our study might fill a blind spot in these literatures: By recording EEG-fMRI during a decision task, we show that theta power increases commonly observed in such tasks might reflect subcortical action selection processes. Arguably, the striatum is far away from the scalp and thus unlikely to be the direct neural source of midfrontal theta oscillations. It is possible that striatal action selection processes modulate activity in parts of midfrontal (or motor) cortex, reflected in the amplitude of theta power over the scalp (see also our EEG-informed fMRI analyses in Supplementary Material 17). This finding suggests that scalp EEG can give insights into evolving action selection in the striatum, which is not visible in resting-state recordings but can only be studied using appropriate task designs.

In contrast to our study, previous findings have reported elevated theta power mostly in situations of cognitive conflict (Cavanagh and Frank 2014; Cohen 2014), including our own EEG study using the same task (Swart et al. 2018). Importantly, however, these studies usually observe strong theta rises for *any* action, with conflict-induced theta constituting a minor increase on top of this much larger rise (Cohen and Cavanagh 2011; Swart et al. 2018). While conflict-induced theta was absent in our data, action-induced theta was strongly present—which might be especially visible in Go/NoGo tasks such as in this study, but concealed in other paradigms that only feature active responses. We speculate that both phenomena are related and reflect the evolving value of making a Go action: Theta power rises prior to Go actions, but even further in situations of cognitive conflict. If response thresholds in striatal pathways are elevated during conflict, theta may not reflect a cortical top-down “trigger” that drives threshold elevation, but rather the extra bits of accumulated evidence in the striatum that follow from such elevated response thresholds. This alternative account of midfrontal theta power modulation provides a putative unifying explanation for both action- and conflict-induced theta increases.

### Early vmPFC Valence Signals Shape Action Selection

So far, we have suggested that striatal BOLD and theta power signals reflect how evidence for action is accumulated, putatively reflecting the value of work (i.e., the physical effort of taking action). This interpretation leaves open what drives this evidence accumulation—and does so differently for reward and punishment prospects, leading to the observed expression of motivational biases in behavior. Any neural “source” of these biases should show differential activity in response to Win and Avoid cues. While valence coding was weak and spatially heterogeneous in the striatum, it clearly emerged in vmPFC (positively) and ACC (negatively). Particularly, the vmPFC appears to be a likely candidate source of motivational biases given that a wealth of previous studies (Haber and Knutson 2010; Bartra et al. 2013) has shown vmPFC BOLD to encode the expected outcomes. In behavior, cue valence affected both the probability and the speed of making a Go response, as people responded more often and faster to Win cues than to Avoid cues. A follow-up trial-by-trial analysis showed that the vmPFC is likely involved in eliciting this motivational bias, as fluctuations in vmPFC signal predicted response times also within each valence condition. This finding is consistent with the idea that valence information in this region feeds into fronto-striatal loops and gives rise to motivational biases in behavior. Of note, the region of ACC encoding cue valence did not significantly correlate with midfrontal theta power, even though trial-by-trial ACC BOLD did correlate with RTs. Taken together, in line with past theories of recurrent fronto-striatal loops (Alexander et al. 1986; Mink 1996; Middleton and Strick 2000; Gurney et al. 2001), our result suggest that vmPFC encodes cue valence at an early time point and then biases the motivation for active responses, that is, the value of work (Hamid et al. 2016; Berke 2018), in the striatum.

vmPFC BOLD correlated negatively with midfrontal theta power very early after cue onset (Fig. 4*C*), consistent with previous EEG-fMRI findings (Scheeringa et al. 2008, 2009; Hauser et al. 2015) as well as electrophysiological data in humans (Harris et al. 2011; Hunt et al. 2012) and animals (Van Wingerden et al. 2010; Vinck et al. 2010; Seo et al. 2012; Knudsen and Wallis 2020). The vmPFC shows a more positive BOLD response to Win (compared with Avoid) cues. The observed negative vmPFC–theta correlations are in line with previous findings showing that both vmPFC and midfrontal theta power encode valence, though with opposite signs: vmPFC BOLD is typically higher for positive than negative events, while the opposite holds for midfrontal theta (and midfrontal BOLD signal) (Shackman et al. 2011; Cavanagh, Figueroa, et al. 2012; Cavanagh, Zambrano-Vazquez, et al. 2012; Braem et al. 2017). Our results indicate that vmPFC encodes cue valence very soon after this information becomes available, indexed in midfrontal theta power.

In contrast to the negative vmPFC–theta correlation immediately following cue onset, action-related theta and positive striatum–theta correlations occurred later, around the time of response (Cohen 2014). Although both vmPFC and striatal BOLD correlated with power in the same frequency band, correlations may well reflect different neural processes. Compared with vmPFC–theta correlations, striatum–theta correlations and in particular action-related theta exhibited a more centroparietal (rather than midfrontal) topography of rather short duration (for a discussion of the burst-like modulations of ongoing theta oscillations, see Cohen 2014). Of note, timing and topography of action-related theta in the EEG-only analysis were similar to the “centroparietal positivity”/P300, which has been suggested to reflect perceptual evidence accumulation (O’Connell et al. 2012; Kelly and O’Connell 2013; Philiastides et al. 2014; Twomey et al. 2015). However, this action-related theta signal was not visible in cue-locked ERP analyses and thus apparently not phase-locked (see Supplementary Material 8). Taken together, early vmPFC–theta correlations likely reflect cue valence processing, while late striatum–theta correlations likely reflect the motivation for a final action.

### Caveats and Open Questions

A priori, we expected elevated midfrontal theta power in situations of motivational incongruency between biases and required actions. Instead of theta power, we observed a transient increase in midfrontal alpha power, which specifically occurred on incongruent trials on which participants successfully overcame biases. Trial-by-trial midfrontal alpha power was negatively correlated with BOLD signal in dorsolateral prefrontal cortex and supramarginal gyrus. While we are not aware of previous literature reporting elevated frontal alpha in conflict situations, the observed associations with BOLD in regions of the fronto-parietal attention network might point at midfrontal alpha reflecting an unspecific mechanism of focused attention and increased task engagement (Brier et al. 2010; Harris et al. 2013; Helfrich and Knight 2016), which might help to retrieve and focus on learned stimulus–response associations (Buschman et al. 2012). Of note, while heightened attention is typically associated with decreased (posterior) alpha, there have been findings of increased alpha, as well (Van Der Meij et al. 2016). Nonetheless, future research is needed to understand the role midfrontal alpha might play in overcoming motivational conflict.

The absence of elevated theta power in situations of cognitive conflict could perhaps be due to relatively low performance in the current study compared with previous studies using the same task (Swart et al. 2017, 2018). This reduced performance is likely due to the fMRI environment and associated necessary task changes (longer and jittered response–outcome intervals) and may explain the inconsistency with previous findings (Swart et al. 2018). If participants learned the required action of a cue less well, they would be less aware of conflict between motivational bias and action requirements. Furthermore, reduced performance resulted in relatively fewer trials on which participants successfully overcame motivational biases, leaving less statistical power to test neural hypotheses on bias suppression. This might explain why we did not observe conflict-related midfrontal theta increases and why evidence for increased BOLD signal in pre-SMA during conflict was rather weak. Thus, our findings do not undermine the role of theta in motivational conflict but rather highlight a putatively complementary role of theta in the motivation of actions.

Finally, the response-locked nature of the observed theta power difference raises the question whether this finding might simply reflect motor execution. Control analyses indicated that the signal was modulated by response hand and accuracy, which can be expected from a signal that reflects decision variables such as response conflict or threshold variability, but not from a signal that reflects simple motor execution or even a signal artifact. Moreover, the observation that striatal BOLD was the best predictor of trial-by-trial midfrontal theta power (rather than BOLD in motor cortices) again speaks for theta reflecting a preresponse, decision-related processes.

## Conclusion

In sum, participants in this simultaneous EEG-fMRI study exhibited strong motivational biases and relatively poor instrumental learning in a motivational Go–NoGo learning task. This feature likely has rendered our setup suboptimal for isolating the predicted fronto-striatal mechanisms involved in suppressing motivational biases. However, the presence of these strong (relatively uncontrolled) motivational biases enabled us to further dissect the mechanisms of bias expression. Specifically, the finding that, despite strong valence effects on behavior, striatal BOLD indexed the selected action (rather than cue valence) indicates that the striatum is unlikely to play a role in generating the motivational bias. Rather, striatal BOLD might selectively reflect the motivation to show an active Go response. The finding of strong cue valence signaling in the vmPFC, which also predicted reaction times on a trial-by-trial basis, suggests that the motivational bias might instead arise from the vmPFC. The negative association of this vmPFC signal with early midfrontal theta suggests that the vmPFC processes valence information very early after cue onset and may subsequently shape action selection. One putative mechanism through which the vmPFC could shape action selection is the modulation of the rate of evidence accumulation toward a Go response. Besides the negative correlation of vmPFC BOLD with midfrontal theta power early after cue onset, the positive correlation of striatal BOLD with late midfrontal theta power concurs with a complementary role for the striatum in the eventual decision to execute an active response. Together, these findings suggest a dual nature of midfrontal theta power, with early components reflecting valence processing in the vmPFC and late components reflecting motivation for action in the striatum. Taken together, our results are in line with “value of work” theories of the role of fronto-striatal loops in the evaluation of whether to perform an active response.

## Supplementary Material

Algermissen2020_CerCor_SupplementaryMaterial_FinalProofs_bhab391

## Data Availability

All raw data are available under: https://doi.org/10.34973/pezs-pw62. All code required to achieve the reported results as well as preprocessed data and fMRI results are available under: https://doi.org/10.34973/2t72-bj41. In line with requirements of the Ethics Committee and the Radboud University security officer, potentially identifying data (such as imaging data) can only be shared to identifiable researchers. Hence, researchers requesting access to the data have to register and accept a data user agreement; access will then automatically be granted via a “click-through” procedure (without involvement of authors or data stewards). Code will be maintained under https://github.com/johalgermissen/Algermissen2021CerCor, with a permanent copy at the time of publication under https://github.com/denoudenlab/Algermissen2021CerCor. Group-level unthresholded fMRI z-maps are available on Neurovault (https://identifiers.org/neurovault.collection:11178).

## Notes

We thank Emma van Dijk for assistance with data collection, Michael J. Frank for helpful discussions, Tobias U. Hauser and Laurence Hunt for sharing code for fMRI-informed EEG analyses (https://github.com/tuhauser/TAfT), and the weekly Donders M/EEG meeting for discussions of these results and many helpful suggestions. *Conflict of Interest*: All authors declare that no competing interests exist.
