# Supplementary material for: Striatal BOLD and midfrontal theta power express motivation for action
Source: Cereb Cortex. 2021 Nov 24;32(14):2924–42. doi: 10.1093/cercor/bhab391 (PMC9290551; doi:10.1093/cercor/bhab391)
Supplement: Algermissen2020_CerCor_SupplementaryMaterial_FinalProofs_bhab391 [file algermissen2020_cercor_supplementarymaterial_finalproofs_bhab391.zip › Algermissen2020_CerCor_SupplementaryMaterial_FinalProofs_bhab391.docx]

Striatal BOLD and midfrontal theta power express motivation for action:

Supplementary Materials

Johannes Algermissen^1*^, Jennifer C. Swart^1^, René Scheeringa^1,2^, Roshan Cools^1,3^, Hanneke E.M. den Ouden^1*^

^1^ Radboud University, Donders Institute for Brain, Cognition and Behaviour, Nijmegen, The Netherlands

^2^ Erwin L. Hahn Institute for Magnetic Resonance Imaging, University of Duisburg‐Essen, Essen, Germany

^3^ Department of Psychiatry, Radboud University Medical Centre, Nijmegen, The Netherlands

* Contract Address: Montessorilaan 3B, 6525 HR Nijmegen, Telephone: + 31 (0)24 36 12618, [j.algermissen@donders.ru.nl](mailto:j.algermissen@donders.ru.nl) (J.A.); [h.denouden@donders.ru.nl](mailto:h.denouden@donders.ru.nl) (H.E.M.d.O.)

Running title: STRIATUM AND THETA EXPRESS MOTIVATION FOR ACTION

Supplementary Materials

Contents

[S01: Behavioral, fMRI, and EEG analyses with only the 30 participants included in EEG-fMRI analyses 3](#_Toc80965619)

[S02: Anatomical masks (for small-volume corrected analyses) and conjunctions of anatomical and functional masks (for fMRI-informed EEG analyses) 8](#_Toc80965620)

[S03: Regressors and contrasts in fMRI analyses 11](#_Toc80965621)

[S04: Significant BOLD clusters in the valence, action, and congruency contrasts 12](#_Toc80965622)

[S05: Changes in effects on fMRI BOLD signal over blocks 14](#_Toc80965623)

[S06: fMRI results for correct trials only 16](#_Toc80965624)

[S07: ERPs as function of action and valence 18](#_Toc80965625)

[S08: Conflict-related alpha power after ERPs are subtracted 20](#_Toc80965626)

[S09: Alpha signal as a function of cue valence, required action, and correctness 21](#_Toc80965627)

[S10: EEG TF power as a function of action and valence across correct and incorrect trials 23](#_Toc80965628)

[S11: Plots and tests of the evidence accumulation hypothesis 25](#_Toc80965629)

[S12: Correlation of EEG power with head motion 28](#_Toc80965630)

[S13: Increase in time-frequency power relative to baseline for each cue valence x performed action pairing 31](#_Toc80965631)

[S14: Theta and beta power for left vs. right hand responses 32](#_Toc80965632)

[S15: Supplementary fMRI-inspired EEG results in time-frequency space 34](#_Toc80965633)

[S16: Supplementary fMRI-inspired EEG results in time space (ERPs) 36](#_Toc80965634)

[S17: EEG-informed fMRI analyses 38](#_Toc80965635)

[References 40](#_Toc80965636)

# S01: Behavioral, fMRI, and EEG analyses with only the 30 participants included in EEG-fMRI analyses

We repeated the behavioral, fMRI, and EEG analyses reported in the main text while excluding the six participants that were also not included in the fMRI-inspired EEG analyses reported in the main text: two participants due to fMRI co-registration failure (which were also not included in the fMRI-only analyses), and five further participants due to large outliers on the *b*-maps in the fMRI-inspired EEG analyses.

In this subgroup, similar to the entire sample, participants performed significantly more Go responses to Go cues than NoGo cues (Required action: χ^2^(1) = 25.77, *p* < .001; see Fig. S01A panels A-B). Furthermore, participants showed a motivational bias, as they performed more Go actions for Win cues than Avoid cues (Valence: χ^2^(1) = 18.87, *p* < .001). The interaction of Valence x Required Action was not significant (χ^2^(1) = 0.13, *p* = .910). Similarly, when making a (Go) response, participants responded faster when this was correct (Go cues; irrespective of whether a left or right hand response required) than when this was incorrect (NoGo cues) (Required action: χ^2^(1) = 21.02, *p* < .001). Furthermore, a motivational bias was present also in RTs, with significantly faster responses to Win than Avoid cues (Valence: χ^2^(1) = 37.31, *p* < .001). Again, the interaction was not significant (χ^2^(1) = 2.25, *p* = .134; see Fig. S01A panel C). In sum, all behavioral results also held in this subsample.

| 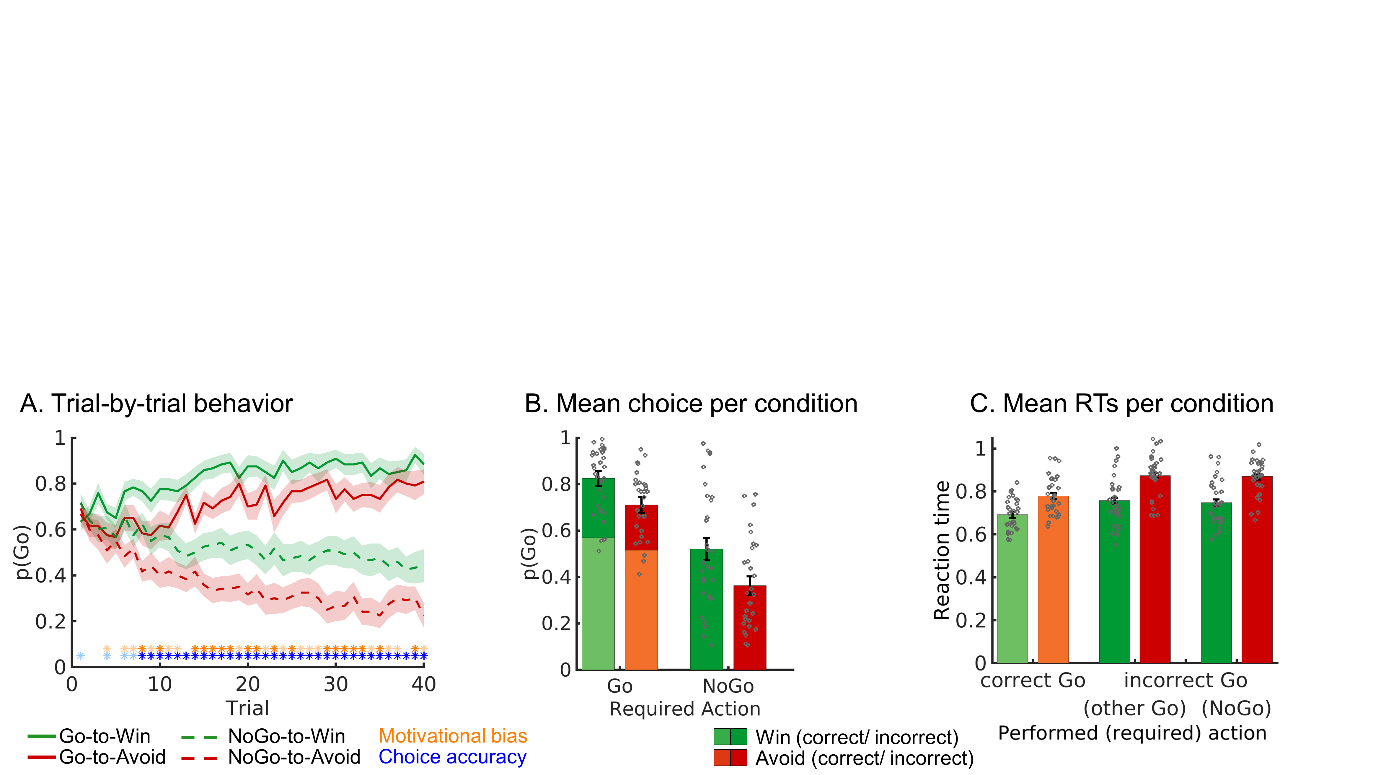 |
| --- |
| *Figure S01A.* Motivational Go/NoGo learning task performance in the subgroup of 29 participants included in the fMRI-inspired EEG analyses. (A) Trial-by-trial proportion of Go responses (±SEM) for Go cues (solid lines) and NoGo cues (dashed lines). Shadows indicate standard errors for per-condition-per-participant means across participants using the Cousineau-Morey method (Morey 2008). The motivational bias is defined as the tendency to make more Go actions to Win than Avoid cues (i.e., green lines are above red lines). Additionally, participants clearly learn whether to make Go actions or not (solid lines go up, dashes lines go down). Orange asterisks below indicate trial-by-trial significance of motivational bias, blue asterisks indicate performance accuracy above chance (i.e., correct GoLeft, GoRight or NoGo response) (light color: p < .05 uncorrected; dark color: p < 0.0013; Bonferroni corrected for number of trials). (B) Mean (±SEM) proportion Go responses per cue condition (points are individual participants’ means). Proportion Go responses is higher for Go than NoGo cues, indicative of task learning, and higher for Win than Avoid cues, reflecting the influence of motivational biases on behavior. (C) Mean (±SEM) reaction times for correct and incorrect Go responses, the latter split up in whether the other Go response or the NoGo response would have been correct (points are individual participants’ means). Participants respond faster on correct than on incorrect Go responses and faster to Win than Avoid cues, reflecting the influence of motivational biases on behavior. |

In our fMRI analyses, when testing for differences in BOLD signal between Win and Avoid cues, there were again no significant clusters in the striatum in a whole-brain corrected analysis. When restricting our analyses to an anatomical mask of the striatum, BOLD signal in both left (*z*_max_ = 4.35, *p* = .00485, MNI peak coordinates: xyz = [-8 4 4]) and right medial caudate nucleus (*z*_max_ = 3.98, *p* = .0121, xyz = [12 6 6]) was higher for Avoid than Win cues as reported in the main text (Fig. S01B panel F), while differences in left posterior putamen reported in the main text were not significant any more (Fig. S01B panel E). Furthermore, at a whole-brain level cluster correction, BOLD signal was again higher for Win compared to Avoid cues in vmPFC (*z*_max_ = 5.09, *p* = 2.24e-16, xyz = [-6 44 2]), dlPFC (*z*_max_ = 5.01, *p* = .000142, xyz = [16 48 48]), PCC (*z*_max_ = 4.34, *p* = .000297, xyz = [6 -44 32]), and left amygdala/ hippocampus (*z*_max_ = 4.36, *p* = .0162, xyz = [-20 -2 -22]). There were additional clusters in left vlPFC (z_max_ = 4.30, *p* = .00702, xyz = [28 36 -10]), left middle temporal gyrus (z_max_ = 3.76, *p* = .014, xyz = [-62 -18 -12]),) right middle temporal gyrus (z_max_ = 3.92, *p* = .014, xyz = [62 -18 -6]), left angular gyrus (*z*_max_ = 4.99, *p* = 5.07e-6, xyz = [-44 -56 20]), and right amygdala/ hippocampus (*z*_max_ = 4.52, *p* = .0223, xyz = [20 -6 -20]) not featured in the results in the main text (Fig S01B panel B). Conversely, in line with results reported in the main text, BOLD was higher for Avoid stimuli in dorsal ACC (*z*_max_ = 4.12, *p* = 1.15e-05, xyz = [2 36 46]), left insula (*z*_max_ = 4.47, *p* = .0014, xyz = [-28 22 0]), left superior frontal gyrus (*z*_max_ = 4.12, *p* = .00398, xyz = [-22 -4 56]), right insula (*z*_max_ = 4.07, *p* = .00148, xyz = [32 26 0]), left vlPFC (*z*_max_ = 4.47, *p* = .00697, xyz = [-32 62 8]), right superior frontal gyrus (*z*_max_ = 3.99, *p* = .00588, xyz = [22 -4 52]), and right precuneous (*z*_max_ = 4.60, *p* = .00156, xyz = [8 -64 54]), in line with results reported in the main text (see Fig. S02D). In addition, we observed clusters in left frontal pole (*z*_max_ = 4.67, *p* = .00702, xyz = [-32 62 8]) and left angular gyrus (*z*_max_ = 3.98, *p* = .0162, xyz = [-38 -56 48]).

When correlating RTs and BOLD signal, there was again a significantly negative correlation between RTs and vmPFC BOLD, *t*(29) = -3.89, p < 0.001, *d* = -0.71, and a significantly positive correlation between RTs and ACC BOLD, *t*(29) = 7.41, *p* < 0.001, *d* = 1.35 (see Fig. S01B panel H).

When testing for differences in BOLD signal between responses, we observed again significantly higher BOLD signal for *Go* than *NoGo* action in the entire striatum (bilateral caudate nucleus, putamen, and nucleus accumbens), thalamus, and bilateral cerebellum (*z*_max_ = 7.05, *p* = 0, xyz = [-12 -24 10]), ACC (*z*_max_ = 6.87, *p* = 9.04e-05, xyz = [0 8 42]), left motor cortex (z_max_ = 4.59, *p* = .00915, xyz = [-54 -22 -24], Fig. S02F), in line with results reported in the main text. Furthermore, there was an additional (separate) cluster in left frontal pole (*z*_max_ = 4.23, *p* = .0124, xyz = [-28 42 6]. Again, there were not clusters with higher BOLD signal for NoGo than Go responses (Fig S01B panel C).

When testing for differences in BOLD signal between bias-incongruent and bias-congruent actions, there were no clusters at a whole-brain cluster level significance correction, and—different from results report in the main text—also not when restricting the analysis to a mask comprising ACC and pre-SMA (Fig S01B panels D and G).

| 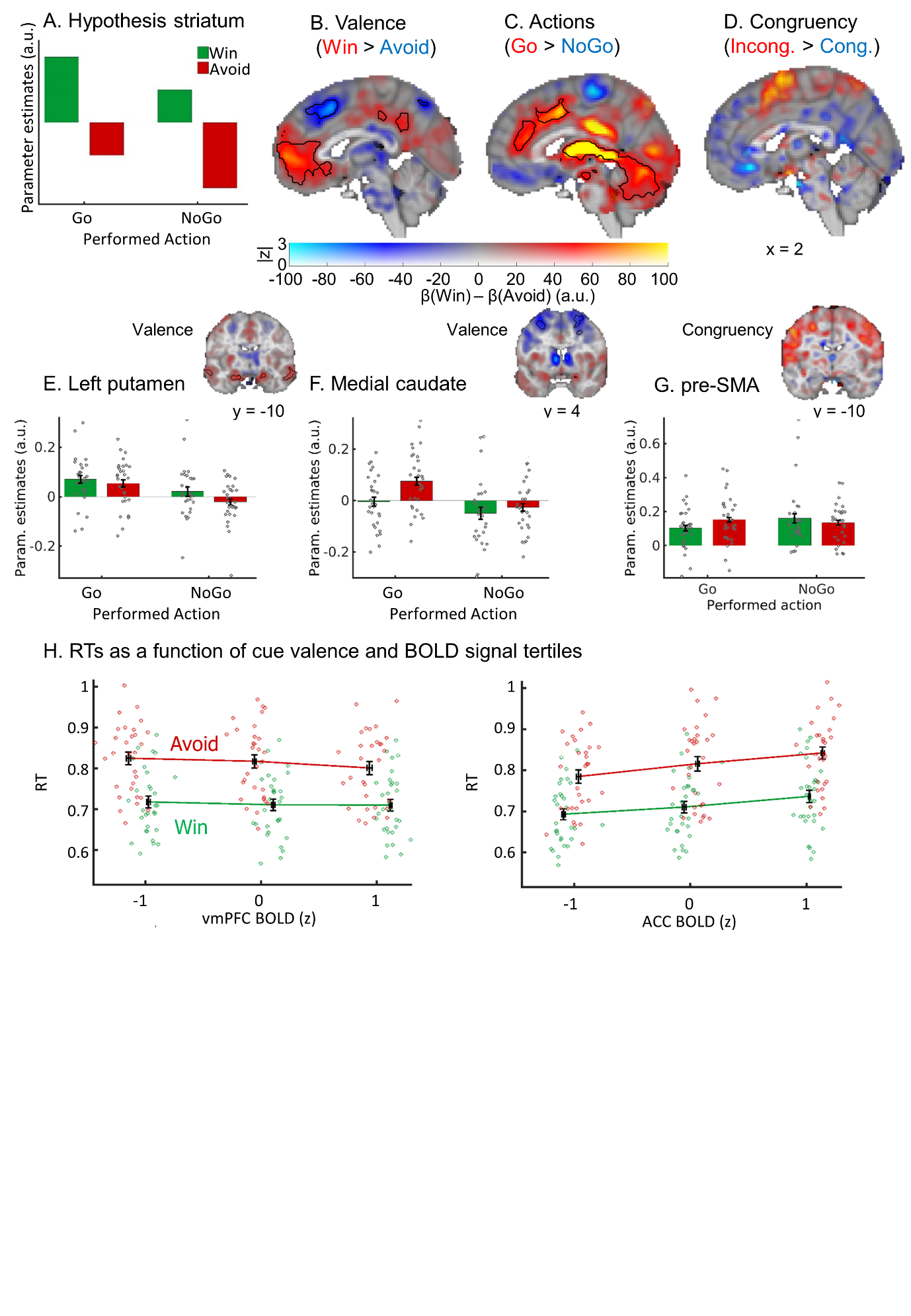 |
| --- |
| *Figure S01C.* BOLD signal as a function of cue valence, performed action, and congruency in the subgroup of 29 participants included in the fMRI-inspired EEG analyses. (A) We hypothesized striatal BOLD to encode cue valence (strong main effect of valence), with an attenuation of this valence signal when actions incongruent with bias-triggered actions were performed (weak main effect of action). (B) BOLD signal was significantly higher for *Win* compared to *Avoid* cues in ventromedial prefrontal cortex (vmPFC; whole brain corrected) and left putamen (small-volume corrected), but higher for *Avoid* compared to *Win* cues in ACC and medial caudate (small-volume corrected). (C) BOLD signal was significantly higher for *Go* compared to *NoGo* actions in the entire striatum as well as ACC, thalamus, and cerebellum (all whole-brain corrected). (D) Based on the plot, it appears that BOLD signal was higher for bias-incongruent actions than bias-congruent actions in pre-SMA, but contrary to the results reported in the main text, this was not significant. B-D. BOLD effects displayed using a dual-coding data visualization approach with color indicating the parameter estimates and opacity the associated *z*-statistics. Black contours indicate statistically significant clusters (p < .05, whole-brain corrected). (E-G) Mean beta weights per task condition (x-axis) per participant (individual grey dots) in significant clusters in left putamen, medial caudate and pre-SMA (significant in small-volume correction). (E) It appears that left posterior putamen encoded valence positively (higher BOLD for Win than Avoid cues), but contrary to results reported in the main text, this was not significant. (F) Medial caudate encoded valence negatively (higher BOLD for Avoid than Win cues). (G) Extracted BOLD signal from pre-SMA to illustrate (non-significant) congruency effects. (H) Reaction times (RTs) as a function of cue valence and BOLD signal tertiles (z-standardized) per participant (individual dots; x-location relative to all other participants). RTs were significantly predicted by BOLD signal in vmPFC (positively) as well as by BOLD signal in ACC and striatum (negatively). BOLD-RT correlations were independent of cue valence. |

In our EEG analyses, there was no significant difference between incongruent than congruent trials in the theta band (*p* = .236). In the alpha band, it was marginally significant (*p* = .052), most strongly around 200–325 ms after cue onset (Fig. S01C panels E-F). A permutation test on the broadband (1–15 Hz) TF power yielded a significant result (*p* = 0.046).

Furthermore, broadband power (1–15 Hz) was significantly higher on trials with Go actions than NoGo actions (cue-locked: *p* = .002, around 550–1300 ms after cue onset, see Fig. S01C panels A-B; response-locked: *p* = .002, around -150–425 ms relative to responses, see Fig. S01C panels C-D). Overall, all EEG results also held in this subsample.

Taken together, behavioral and EEG analyses yielded identical conclusions as results reported in the main text. In the fMRI analyses, differences between Win and Avoid cues in left posterior putamen and differences in pre-SMA between bias-incongruent and bias-congruent actions were not significant, while all other results were still significant and yielded identical conclusions as results reported in the main text.

| 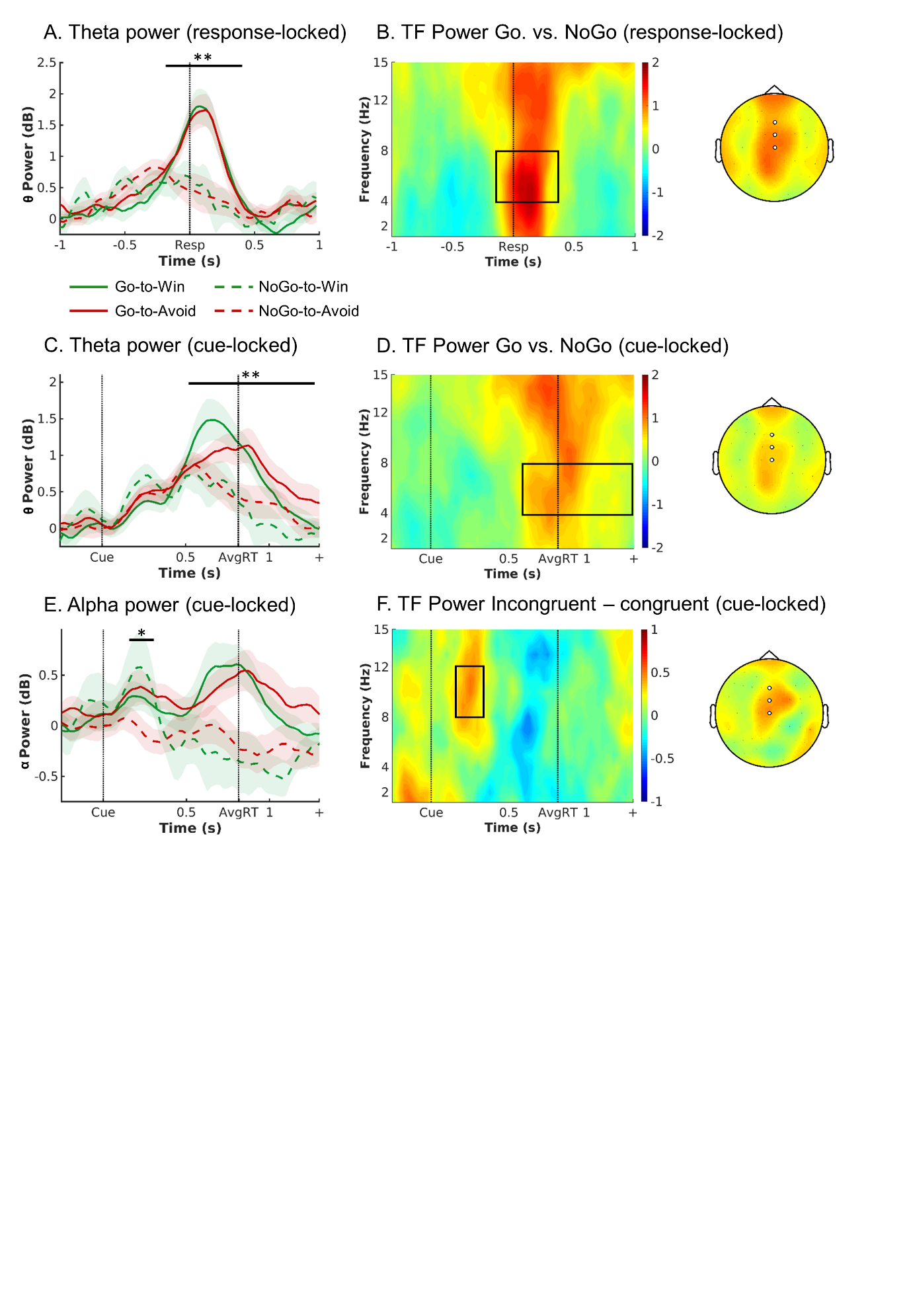 |
| --- |
| *Figure S01C.* EEG time-frequency power as a function of cue valence and action. (A) Response-locked within trial time course of average theta power (4–8 Hz) over midfrontal electrodes (Fz/ FCz/ Cz) per cue condition (correct-trials only). Theta increased in all conditions relative to pre-cue levels, but to a higher level for Go than NoGo trials. There were no differences in theta peak height or latency between Go2Win and Go2Avoid trials. (B) Left: Response-locked time-frequency power over midfrontal electrodes for Go minus NoGo trials. Go trials featured higher broadband TF power than NoGo trials. The broadband power increase for Go compared to NoGo trials is strongest in the theta range. Right: Topoplot for Go minus NoGo trials. The difference is strongest at Cz and FCz electrodes. (C-D) Cue-locked within trial time course and time-frequency power. Theta increased in all conditions relative to pre-cue levels, but to a higher level for Go than NoGo trials, with earlier peaks for Go2Win than Go2Avoid trials. (E) Trial time course of average alpha power (8–13 Hz) over midfrontal electrodes per cue condition (correct trials only; cue-locked). Alpha power transiently increases for both incongruent conditions in an early time window (around 175–325 ms). (F) Left: Time-frequency plot displaying that the transient power increase was focused on the alpha band (8–13 Hz), leaking into the upper theta band. Right: Topoplot of alpha power displaying that this incongruency effect was restricted to midfrontal electrodes (highlighted by white disks). * p < 0.05. ** p < 0.01. Shaded error bars indicate (±SEM). Box in TF plots indicates the time frequency window where t-values > 2. |

# S02: Anatomical masks (for small-volume corrected analyses) and conjunctions of anatomical and functional masks (for fMRI-informed EEG analyses)

| 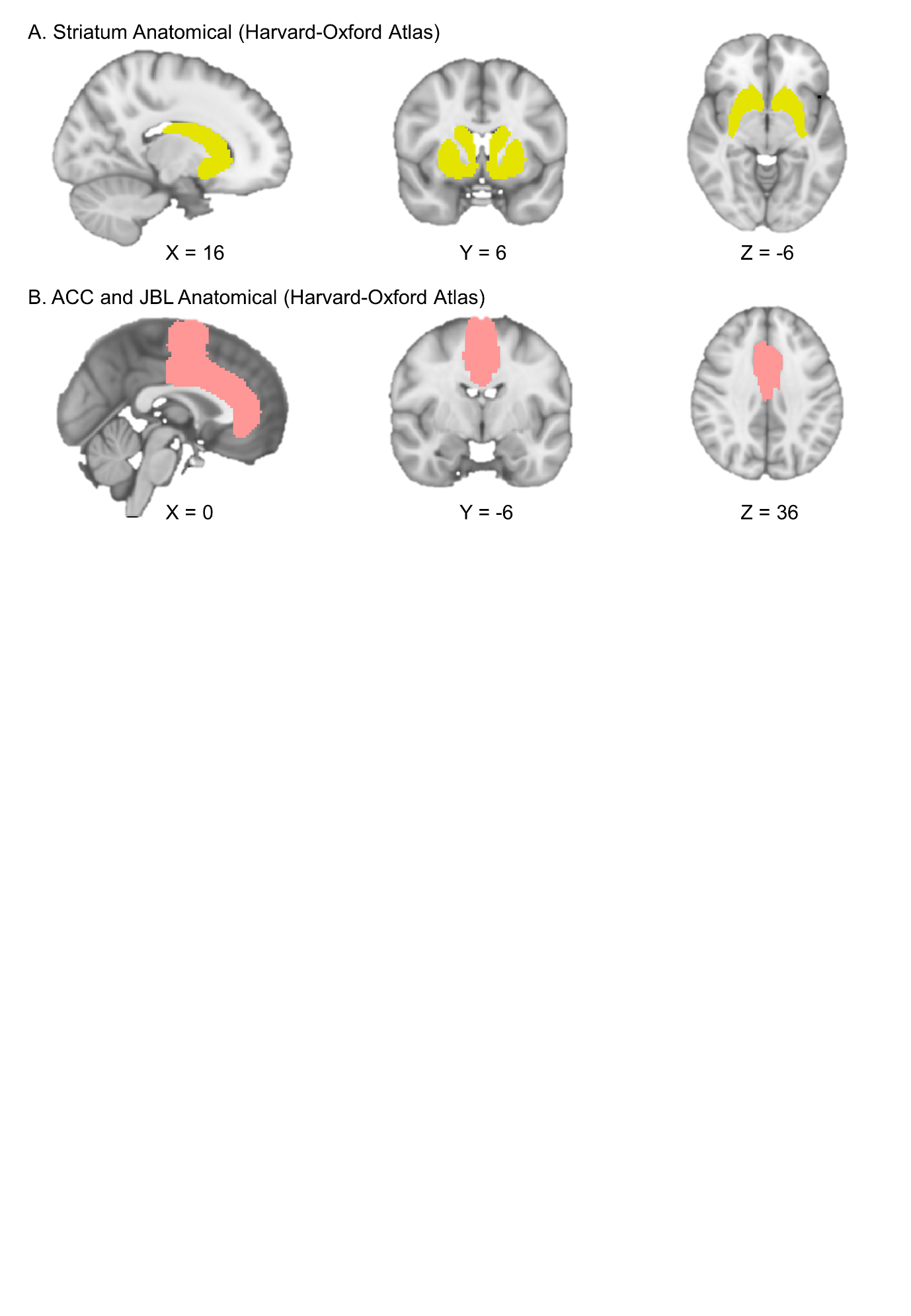 |
| --- |
| *Figure S02A.* Anatomical masks of (A) striatum (yellow, conjunction of bilateral nucleus accumbens, caudate, and putamen) and (B) midfrontal cortex (pink, cingulate cortex anterior and juxtapositional lobule cortex) used for small-volume corrected GLM analyses. All masks were extracted from the probabilistic Harvard-Oxford Atlas, thresholded at 10%. Note that images are in radiological orientation (i.e., left brain hemisphere presented on the right and vice versa). |

| 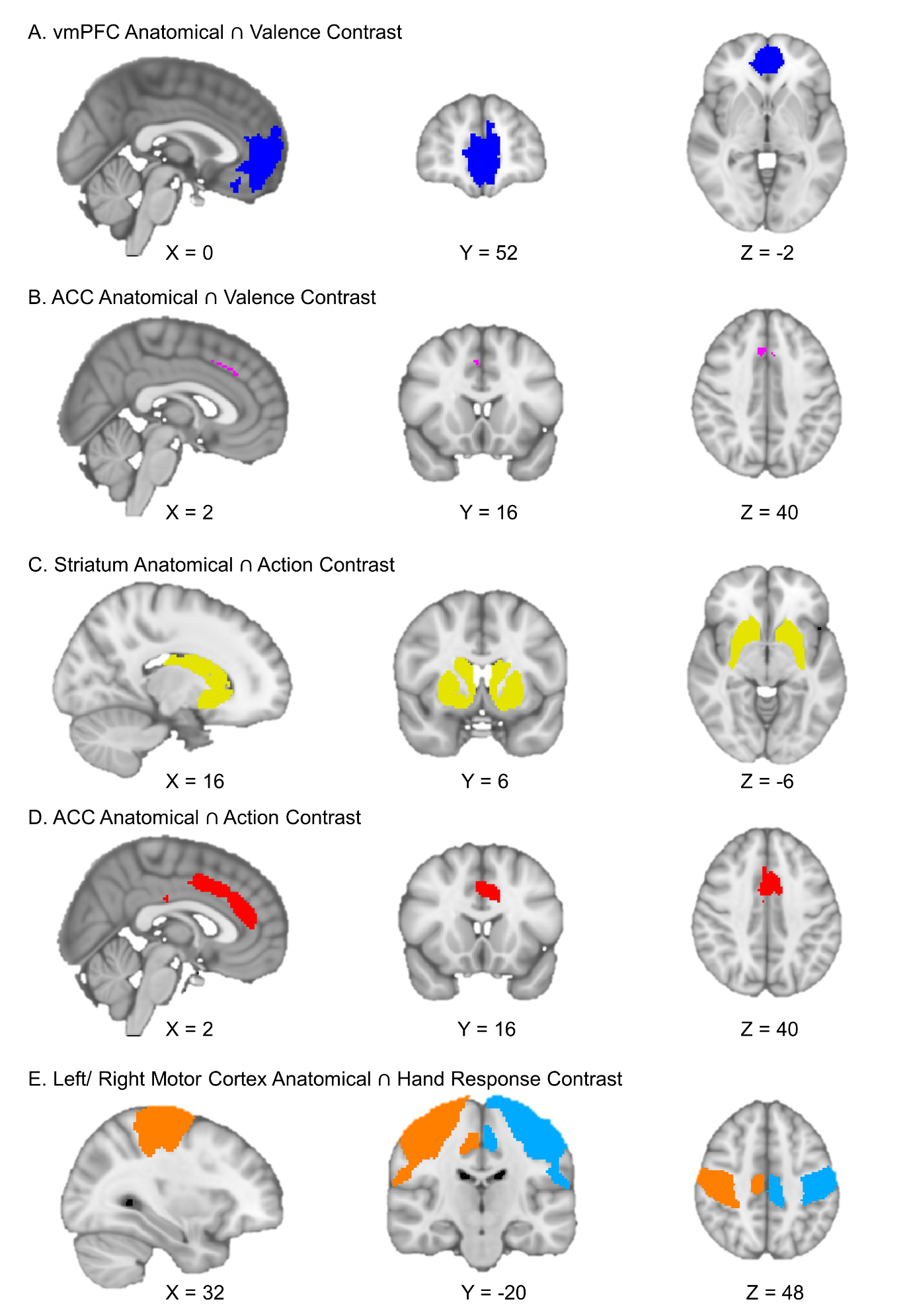 |
| --- |
| *Figure S02B.* Conjunctions of anatomical masks (based on the Harvard-Oxford Atlas) and functional contrasts from fMRI GLM analyses (Valence, Action, and Hand Response contrasts) used for fMRI-informed EEG analyses: (A) vmPFC valence contrast (dark blue, conjunction of frontal pole, frontal medial cortex, and paracingulate gyrus), (B) ACC valence contrast striatum (purple, cingulate cortex anterior), (C) striatum action contrast (yellow, conjunction of bilateral nucleus accumbens, caudate, and putamen), (D) ACC action contrast (red, cingulate cortex anterior), and left (light blue) and right (orange) motor cortices hand response contrast (conjunction of precentral gyrus and postcentral gyrus) used for fMRI-informed EEG analyses. All anatomical masks were extracted from the probabilistic Harvard-Oxford Atlas, thresholded at 10%. Note that images are in radiological orientation (i.e., left brain hemisphere presented on the right and vice versa). |

# S03: Regressors and contrasts in fMRI analyses

Regressors:

- Win2GoOnset: for every trial with Win cue and Go action, at cue onset, duration 1, value +1
- Win2NoGoOnset: for every trial with Win cue and NoGo action, at cue onset, duration 1, value +1
- Avoid2GoOnset: for every trial with Avoid cue and Go action, at cue onset, duration 1, value +1
- Avoid2NoGoOnset: for every trial with Avoid cue and NoGo action, at cue onset, duration 1, value +1
- Handedness: for every trial, at cue onset, value +1 for left hand response, 0 for NoGo response, -1 for right hand response
- Error: for every trial, at cue onset, value +1 for incorrect response, 0 for correct response
- OutcomeOnset: for every trial, at outcome onset, duration 1, value +1 for every trial
- OutcomeValence: for every trial, duration 1, value +1 for positive outcome (reward, no punishment), -1 for negative outcome (no reward, punishment)
- InvalidOutcome: for trials where uninstructed button was pressed, at outcome onset, duration 1, value 1

Nuisance Regressors:

- 6 realignment parameters (obtained from co-registration) per volume
- Mean white-matter signal per volume
- Mean out-of-brain signal per volume
- Separate regressor for each volume where relative displacement > 2mm

| Regressor | 1 | 2 | 3 | 4 | 5 | 6 | 7 | 8 | 9 |
| --- | --- | --- | --- | --- | --- | --- | --- | --- | --- |
| Contrast | Win2GoOnset | Win2NoGoOnset | Avoid2GoOnset | Avoid2NoGoOnset | Handedness | Error | OutcomeOnset | OutcomeValence | InvalidOutcome |
| 1) Valence | 1 | 1 | -1 | -1 |  |  |  |  |  |
| 2) Executed Action | 1 | -1 | 1 | -1 |  |  |  |  |  |
| 3) Conflict | -1 | 1 | 1 | -1 |  |  |  |  |  |
| 4) Hand |  |  |  |  | 1 |  |  |  |  |

# S04: Significant BOLD clusters in the valence, action, and congruency contrasts

| Contrast |  |  |  | Peak coordinates | | |
| --- | --- | --- | --- | --- | --- | --- |
| Brain region | *Z*-value | Cluster size (voxels) | Corrected *p* | x | y | z |
| **Win > Avoid cues** |  |  |  |  |  |  |
| Ventromedial prefrontal cortex, caudal anterior cingulate gyrus | 5.23 | 3533 | 1.06e-18 | 2 | 34 | 12 |
| Left angular gyrus, left supramarginal gyrus | 4.83 | 749 | 2.21e-06 | -42 | -54 | 18 |
| Right dorsolateral prefrontal cortex | 4.98 | 599 | 1.95e-05 | 16 | 46 | 48 |
| Right ventrolateral prefrontal cortex | 4.56 | 596 | 2.04e-05 | 30 | 34 | -12 |
| Right supramarginal gyrus, right middle temporal gyrus | 4.14 | 510 | 7.68e-05 | 66 | -42 | 10 |
| Posterior cingulate gyrus | 4.28 | 460 | .000172 | 8 | -32 | 36 |
| Left hippocampus, left parahippocampal gyrus, left amygdala | 4.72 | 366 | .00085 | -18 | -6 | -24 |
| Left middle temporal gyrus | 3.86 | 346 | .00121 | -60 | -18 | -12 |
| Left precentral gyrus | 4.12 | 303 | .00268 | -34 | -12 | 70 |
| Left ventrolateral prefrontal cortex | 3.97 | 251 | .00734 | -40 | 36 | -14 |
| Right hippocampus, right parahippocampal gyrus, right amygdala | 4.66 | 240 | .00916 | 20 | -6 | -20 |
| Left posterior middle temporal gyrus | 3.88 | 204 | .0194 | -62 | -46 | -8 |
| *ROI in striatum:* |  |  |  |  |  |  |
| Left putamen | 4.00 | 78 | .00979 | -28 | -10 | 6 |
| **Avoid > Win cues** |  |  |  |  |  |  |
| Anterior cingulate cortex, superior frontal gyrus | 4.38 | 690 | 5.13e-06 | 2 | 36 | 46 |
| Left angular gyrus, left superior parietal lobule, left supramarginal gyrus | 4.33 | 428 | .000292 | -38 | -56 | 48 |
| Left insula, left frontal operculum | 3.98 | 303 | .00268 | 34 | 24 | 0 |
| Right insula, right frontal operculum | 4.63 | 292 | .0033 | -28 | 24 | 0 |
| Left ventrolateral prefrontal cortex | 4.72 | 291 | .00336 | -32 | 62 | 8 |
| Right middle frontal gyrus | 4.11 | 213 | .016 | -20 | -2 | 52 |
| Left precuneous | 4.81 | 207 | .0182 | 8 | -66 | 54 |
| *ROI in striatum:* |  |  |  |  |  |  |
| Left medial caudate | 4.27 | 79 | .00979 | -6 | 4 | 2 |
| Right medial caudate | 3.9 | 56 | .0194 | 12 | 8 | 0 |
| **Go > NoGo actions** |  |  |  |  |  |  |
| Cerebellum, bilateral thalamus, bilateral putamen, bilateral caudate, bilateral Nucleus Accumbens, posterior cingulate cortex, anterior cingulate cortex, paracingulate gyrus, bilateral ventrolateral frontal cortex | 7.49 | 26731 | 0 | 26 | -48 | -28 |
| Bilateral precuneous | 5.29 | 595 | .000141 | -10 | -62 | 38 |
| Left postcentral gyrus, left central operculum | 4.69 | 354 | .00378 | -54 | -22 | 22 |
| **NoGo > Go actions** |  |  |  |  |  |  |
| No significant clusters |  |  |  |  |  |  |
| **Incongruent > Congruent actions** |  |  |  |  |  |  |
| No significant clusters |  |  |  |  |  |  |
| *ROI in ACC & pre-SMA:* |  |  |  |  |  |  |
| Pre-SMA | 3.68 | 132 | 0.00431 | 4 | 4 | 66 |
| **Congruent > incongruent actions** |  |  |  |  |  |  |
| No significant clusters |  |  |  |  |  |  |
| *ROI in ACC & pre-SMA:* |  |  |  |  |  |  |
| No significant clusters |  |  |  |  |  |  |

*Table S04*. Significant clusters in the valence, action and congruency contrasts in the fMRI GLM.

# S05: Changes in effects on fMRI BOLD signal over time

After identifying BOLD correlates of cue valence, performed action, and motivational conflict in the whole-brain and small-volume-corrected GLM analyses reported in the main text, we were interested in whether these effects change over the time course of the experiment. For this purpose, we extracted the first eigenvariate of the BOLD signal from the significant clusters above threshold (see Fig. 2 main text; for masks, see S02), fitted an HRF to each trial to obtain the trial-by-trial HRF amplitude (identical procedure to BOLD-RT correlations and fMRI-informed EEG analyses), and analyzed these amplitude as a function of the respective behavioral variable (cue valence, performed action, or motivational conflict), trial number, and their interaction, using mixed-effects linear regression.

Specifically, for vmPFC, ACC, left putamen and medial caudate signal, we fitted the following model (Wilkinson notation):

| *BOLD ~ cueValence * trialNumber + (cueValence * trialNumber\|participant)* |
| --- |

vmPFC signal was strongly modulated by cue valence, χ^2^(1) = 31.313, *p* < .001, with higher signal for Win than Avoid cues. In addition, the main effect of trial number was marginally significant, χ^2^(1) = 3.351, *p* = .067, with signal tending to increase over time. The interaction between valence and trial number was marginally significant as well, χ^2^(1) = 2.959, *p* = .085: The valence effect tended to decrease over time, driven by signal increasing for Avoid cues while staying at a constant high level for Win cues (Fig. S05A).

ACC signal was also strongly modulated by valence, χ^2^(1) = 15.213, *p* < .001, with higher BOLD signal for Avoid than Win cues. There also was a significant main effect of trial number, χ^2^(1) = 6.491, *p* = .011, with signal decreasing over time. The interaction between valence and trial number was marginally significant, χ^2^(1) = 2.935, *p* = .087: The valence effect tended to decrease over time, driven by signal decreasing for Avoid cues while staying at a constant low level for Win cues (Fig. S05B).

Signal in left putamen strongly encoded cue valence, χ^2^(1) = 16.949, *p* < .001, with higher signal for Win than Avoid cues. The main effect of trial number was not significant, χ^2^(1) = 1.265, *p* = .261, and neither was the interaction between valence and trial number, χ^2^(1) = 1.544, *p* = .214 (Fig. S05C).

Signal in medial caudate strongly encoded cue valence, χ^2^(1) = 17.330, *p* < .001, with higher signal for Avoid than Win cues. The effect of trial number was just significant, χ^2^(1) = 3.874, *p* = .049, with signal decreasing over time. The interaction between valence and trial number was marginally significant, χ^2^(1) = 3.769, *p* = .052: The valence effect tended to decrease over time, driven by signal decreasing for Avoid cues while staying at a constant low level for Win cues (Fig. S05D).

For striatal and ACC signal (different mask than for the valence signal reported above), we fitted the following model (Wilkinson notation):

| *BOLD ~ performedAction * trialNumber + (performedAction * trialNumber\| participant)* |
| --- |

For striatal signal, the main effect of action was not significant, *t*(31.14) = 0.031, *p* = .975, while the effect of trial number was strongly significant, *t*(217.09) = -2.773, *p* = .006, with signal decreasing over time. The interaction was marginally significant, *t*(54.61) = 1.736, *p* = .088, driven by signal increasing for Go actions, but decreasing for NoGo actions, such that the action effect in striatum (higher signal for Go than NoGo actions) only emerged over time (because models using likelihood ratio tests failed to converge, *p*-values in this model are instead based on *t*-tests using Satterthwaite’s method as implemented in the R package *lmerTest;* Fig. S05E).

For ACC signal, the main effect of action was not significant, χ^2^(1) = 0.270, *p* = .603, while the main effect of trial number was significant, χ^2^(1) = 5.342, *p* = .021, reflecting overall decreasing signal over time. The interaction between action and trial number was not significant, χ^2^(1) = 0.038, *p* = .845. This inconsistency with our results in the whole-brain GLM analyses might reflect differential weighting of outliers and block-wise signal in FSL’s FEAT vs. lme4’s mixed effects models (Fig. S05F).

For pre-SMA signal, we fitted the following model (Wilkinson notation):

*BOLD ~ conflict * trialNumber + (conflict * trialNumber|participant)*

For pre-SMA signal, there was a significant main effect of conflict, χ^2^(1) = 5.064, *p* = .024, with higher BOLD for bias-incongruent than -congruent action, and a significant negative effect of trial number, χ^2^(1) = 10.530, *p* = .001, with signal decreasing over time. The interaction between conflict and trial number was not significant, χ^2^(1) = 0.142, *p* = .706 (Fig. S05G).

| 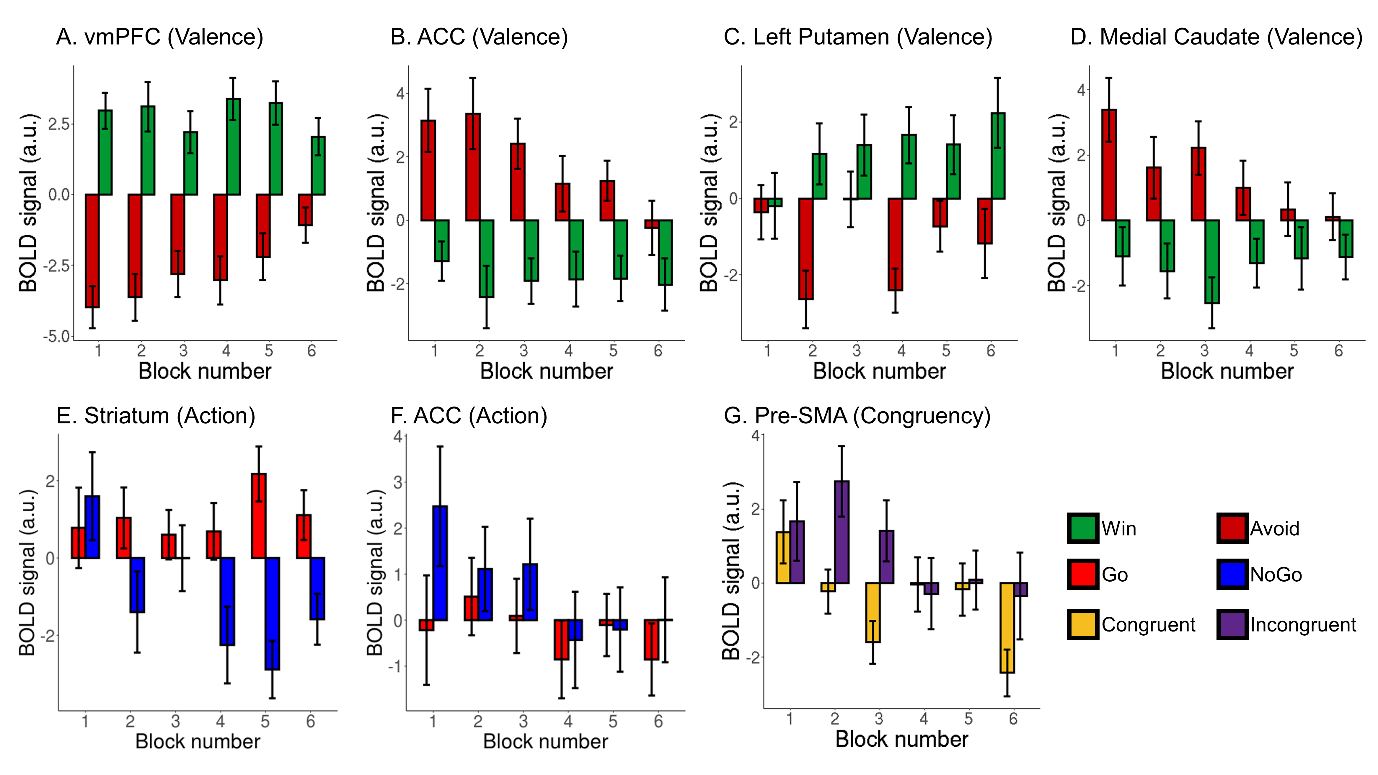 |
| --- |
| *Figure S05*. BOLD signal in significant clusters identified with whole-brain and small-volume-corrected GLM analyses (see main text, Figure 2) as a function of behavioral variables (cue valence, executed action, motivational conflict) and block number. Bar represent means, whiskers present standard errors (per condition over participants, computed via the Cousineau-Morey method). (A) vmPFC encoded cue valence (Win > Avoid), but this effect tended to decrease over time. (B) ACC encoded cue valence (Avoid > Win), but this effect tended to decrease over time. (C) Left putamen encoded cue valence (Win > Avoid). (D) Medial caudate encoded cue valence (Avoid > Win), but this effect tended to decrease over time. (E) Striatum encoded the performed action (Go > NoGo), but this effect only emerged over time. (F) In contrast to whole-brain GLM analyses, ACC did not significantly encode the performed action. (G) Pre-SMA encoded motivational conflict (congruency, incongruent > congruent). |

# S06: fMRI results for correct trials only

EEG and fMRI research have different analytical procedures of dealing with differences between correct and incorrect trials: While fMRI research typically uses multiple linear regression (GLMs), which allows to model error trials by a designated regressor, EEG research typically tests for differences between (categorical) conditions with a (mass-univariate) *t*-test approach. Because we used both approaches in the main text, here, for consistency, we also report fMRI results for regressors defined for correct trials only. Note that this analysis uses less trials than the one featured in the main text and thus has lower statistical power.

We fitted a GLM with eight task regressors, namely the four conditions resulting from crossing cue valence (Win/Avoid) and performed action (Go/NoGo irrespective of Left vs. Right Go) separately for correct and incorrect trials. We again added four regressors of no interest, namely response side (Go left = +1, Go right = -1, NoGo = 0), outcome onset (intercept of 1 for every outcome), outcome valence (reward = +1, punishment = -1, neutral = 0), and invalid trials (invalid buttons pressed and thus not feedback given). Note that compared to the GLM reported in the main text, we did not add an error regressor. This GLM failed to converge for one participant, leaving 33 participants in the group-level analysis.

When comparing BOLD signal between trials with Win cues and with Avoid cues, in the whole-brain corrected analysis, we again observed higher BOLD for Win cues in vmPFC (*z*_max_ = 5.20, *p* = 1.3e-9, xyz = [0 40 2]), as well as left superior lateral occipital cortex (*z*_max_ = 3.59, *p* = .00325, xyz = [-56 -64 30]), and left medial temporal gyrus (*z*_max_ = 3.63, *p* = .00474, xyz = [-70 -14 -14]; Fig. S06A). Conversely, BOLD signal was higher for Avoid cues in left supramarginal gyrus (*z*_max_ = 3.91, *p* = .00235, xyz = [-36 -48 34]) and left ventrolateral prefrontal cortex (*z*_max_ = 2.34, *p* = .00453, xyz = [-26 58 4]) (Fig. S06B). Note that higher activity in ACC for Avoid is clearly visible in Fig. S06B (blue blob), but not statistically significant. Furthermore, analyses using small-volume correction on an anatomical mask of the striatum yielded no clusters of differential BOLD activity, also not in the regions reported in the main text, i.e., in left putamen (Fig. S06E) nor medial caudate (Fig. S06F). Overall, whole-brain results on correct trials only were similar to the results across both correct and incorrect trials reported in the main text, but weaker, suggesting that restricting analyses to correct trials only resulted in a considerable loss in statistical power.

When comparing trials with Go vs. NoGo actions, we observed higher BOLD signal for Go than NoGo actions in clusters in bilateral cerebellum, thalamus, striatum, and ACC (*z*_max_ = 7.01, *p* = 0, xyz = [-30 -50 -30]), right ventrolateral prefrontal cortex (*z*_max_ = 4.38, *p* = 1.79e-07, xyz = [34 48 6]), precuneous (*z*_max_ = 5.21, *p* = 1.97e-05, xyz = [-8 -64 38]), left operculum (*z*_max_ = 4.72, *p* = .000216, xyz = [-52 -22 18]), right supramarginal gyrus (*z*_max_ = 4.99, *p* = .000351, xyz = [-40 -50 36]), left precentral gyrus (*z*_max_ = 4.15, *p* = .00186, xyz = [-44 -18 62]), and right precentral gyrus (*z*_max_ = 3.98, *p* = .00758, xyz = [46 -18 66]; Fig. S06C). This finding is in line with results across both correct and incorrect trials reported in the main text. Conversely, BOLD signal was higher for NoGo than Go trials in left inferior frontal gyrus (*z*_max_ = 4.43, *p* = .0128, xyz = [-58 26 20]), a finding not observed across both correct and incorrect trials reported in the main text.

Finally, when comparing both incongruent and congruent trials, there were again no significant clusters in a whole-brain corrected analysis (Fig. S06D), and also not in an analysis using small-volume correction on midfrontal cortex (Fig. S06G). Again, this null result might be due to a considerate loss in power compared to the results across both correct and incorrect trials reported in the main text.

| 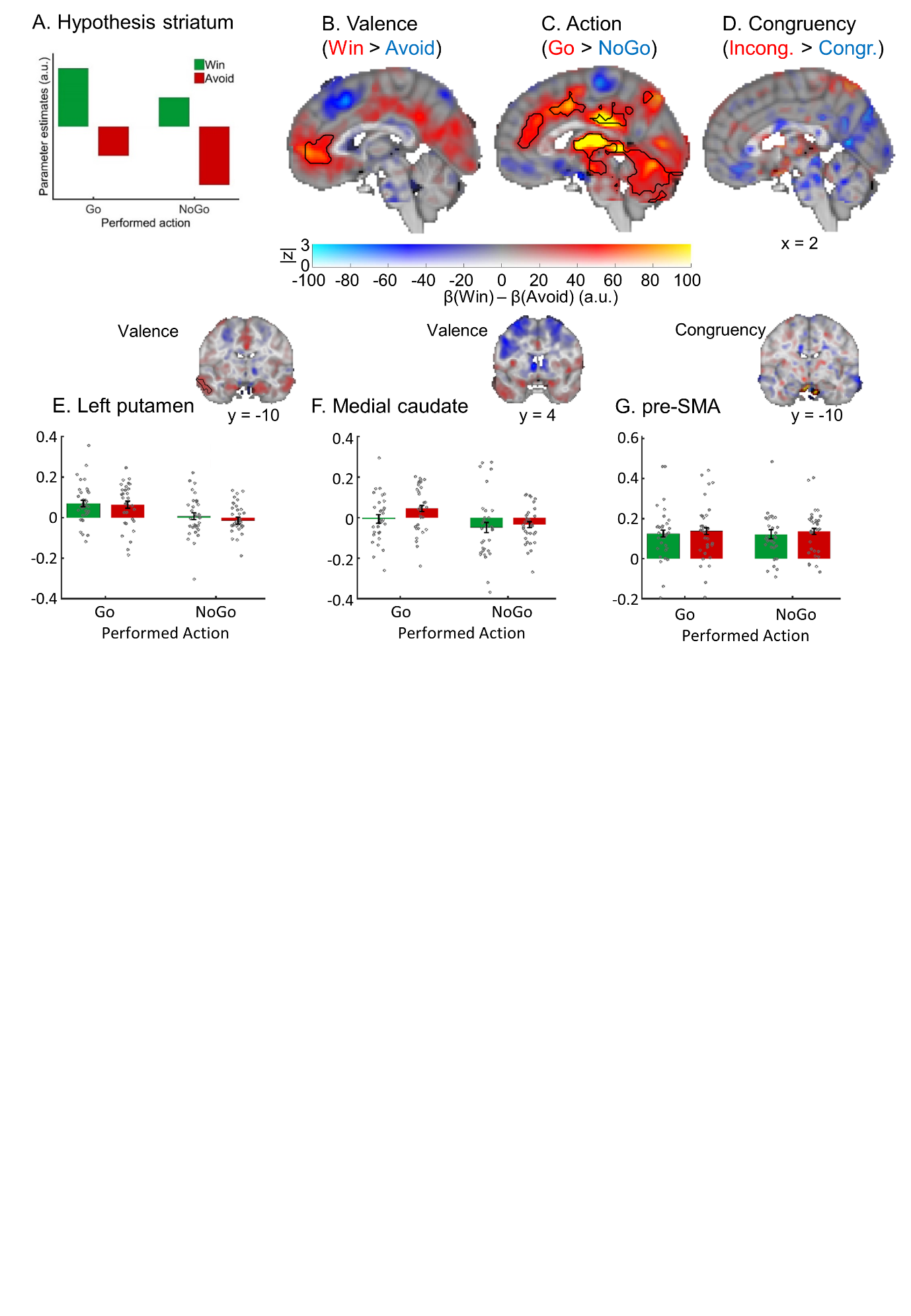 |
| --- |
| *Figure S06.* BOLD signal as a function of cue valence, performed action, and congruency for correct trials only. (A) We hypothesized striatal BOLD to encode cue valence (main effect of valence), with an attenuation of this valence signal when actions incongruent to the bias-triggered actions were performed (main effect of action). (B) BOLD signal was significantly higher for *Win* compared to *Avoid* cues in ventromedial prefrontal cortex (vmPFC; whole brain corrected), but in contrast to analyses across both correct and incorrect trials reported in the main text, BOLD was not significantly higher for *Avoid* compared to *Win* cues in ACC. (C) BOLD signal was significantly higher for *Go* compared with *NoGo* actions in the entire striatum as well as ACC, thalamus, and cerebellum (all whole-brain corrected). (D) BOLD signal was not significantly different between bias-incongruent actions (Go actions to Avoid cues and NoGo actions to Win cues) and bias-congruent actions (Go actions to Win cues and NoGo actions to Avoid cues), also not in the cluster in pre-SMA reported in the main text (small-volume corrected). B-D. BOLD effects displayed using a dual-coding data visualization approach with color indicating the parameter estimates and opacity the associated *z*-statistics. Contours indicate statistically significant clusters (p < .05), either small-volume corrected (striatal and SMA contours explicitly linked to a bar plot) or whole-brain corrected (all other contours). (E) Numerically, left posterior putamen seemed to encode valence positively (higher BOLD for Win than Avoid cues), but in contrast to analyses across both correct and incorrect trials reported in the main text, this was not significant. (F) Numerically, medial caudate seemed to encode valence negatively (higher BOLD for Avoid than Win cues), but in contrast to analyses across both correct and incorrect trials reported in the main text, this was not significant. (G) Extracted BOLD signal from pre-SMA to illustrate (the lack of) congruency effects. |

# S07: ERPs as function of action and valence

Given that the observed phasic alpha increase occurred soon after stimulus onset and much earlier than the theta effect in our previous study (Swart et al., 2018)—although more similar to the timing reported by Cavanagh and colleagues (2013)—we investigated whether conditions differed in evoked rather and induced activity.

First, we again selected correct trials only, computed average ERPs for each condition per participant, and then tested for significant differences between the ERPs for incongruent and congruent trials using permutation tests on the average signal over midfrontal channels (Fz/ FCz/ Cz) in the time period of 0–700 ms post-cue (where evoked potentials occurred in the condition-averaged plot). We found no significant clusters in which the ERPs differed (no clusters above threshold; see Fig. S07A panels A and D). Visual inspection yielded an inconsistent picture such that, if anything, N1, N2 and P3 components tended to be slightly stronger on incongruent trials, while P2 components tended to be stronger on congruent trials. Numerically, when comparing all four conditions, the P2 seemed to be highest and the N2 lowest on Go2Avoid trials, which the opposite was the case for NoGo2Win trials (see Fig. S07B). Such opposite findings cannot explain why both conditions showed an increase in alpha power (see Fig. 3E in the main text), suggesting that the observed alpha power findings are not reducible to evoked activity.

Next, in line with the analyses in time-frequency space, we analyzed ERPs as a function of executed action and cue valence, contrasting trials with Go vs. NoGo actions and trials with Win vs. Avoid actions using permutation tests over the average signal of midfrontal electrodes (Fz/ FCz/ Cz). We found that ERPs differed significantly for Go vs. NoGo responses (*p* = .008) around 200–350 ms after cue onset, reflecting higher P2 (and lower N2) components for Go compared to NoGo responses (Fig. S07A panel B). The peak of the topography of this effect was over left and central frontal electrodes (Fig. S07A panel E).

When contrasting ERPs for Win vs. Avoid cues, we only obtained a marginally significant *p*-value of .068, which was driven by higher signal 420–470 ms after cue onset (Fig. S07A panels C and F). This difference occurred over midfrontal electrodes at the moment that the evoked signal rose towards the P3, but did not reflect differences in any of the component peaks.

| **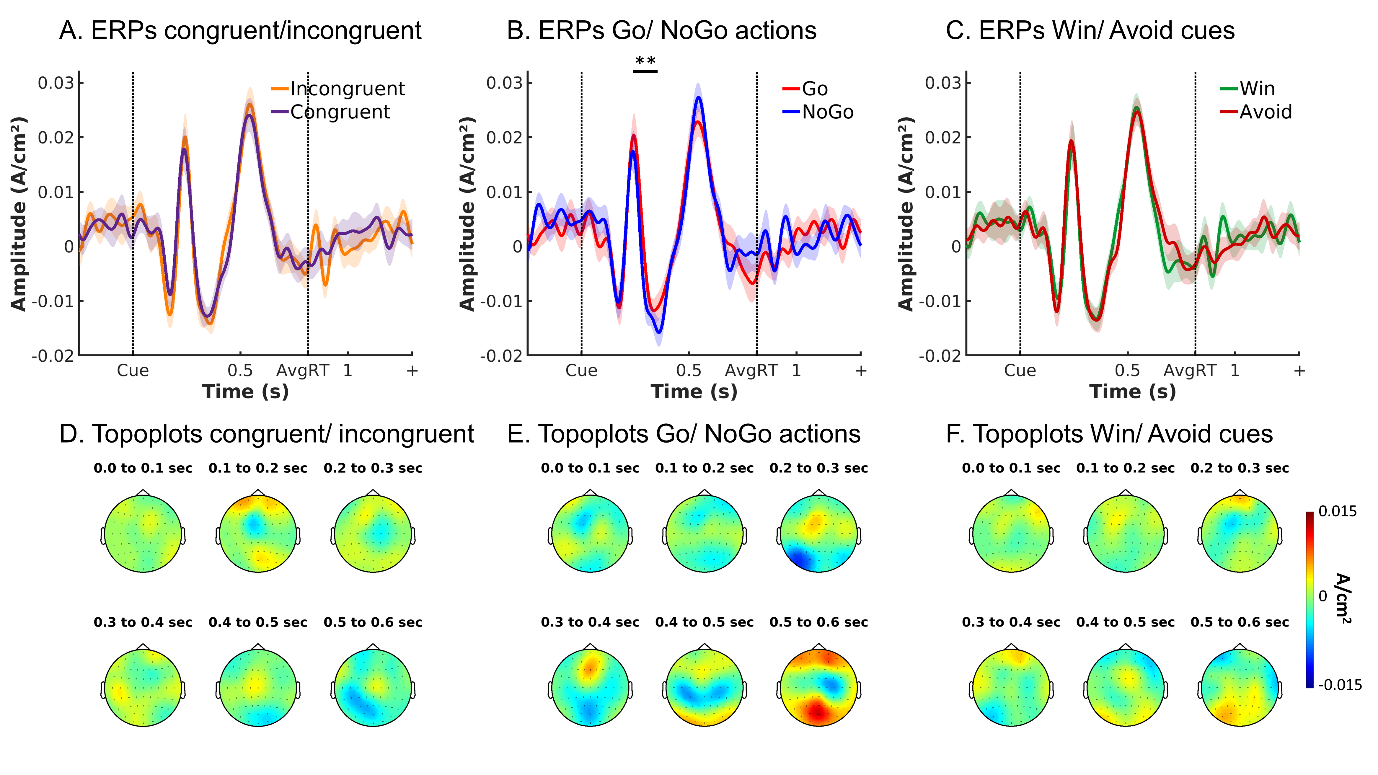** |
| --- |
| *Figure S07A*. ERPs (±SEM) as a function of congruency, action, and cue valence over midfrontal electrodes (Fz/FCz/Cz; correct trials only). (A) There was no difference in midfrontal between congruent and incongruent trials, showing that the transient alpha effect observed on incongruent trials (main text Fig. 3E-F) was not reducible to evoked activity. (B) The frontal P2 component was stronger for Go compared to NoGo actions (and N2 respectively weaker). ** *p* < 0.01. (C) There was no difference between ERPs on Win and Avoid cues—apart from a small difference when the signal rises towards the P3 peak. (D-F) Topoplots displaying differences in ERPs between (D) congruency, (E) action, and (F) valence conditions in steps of 100 ms from 0 to 600 ms. |

| **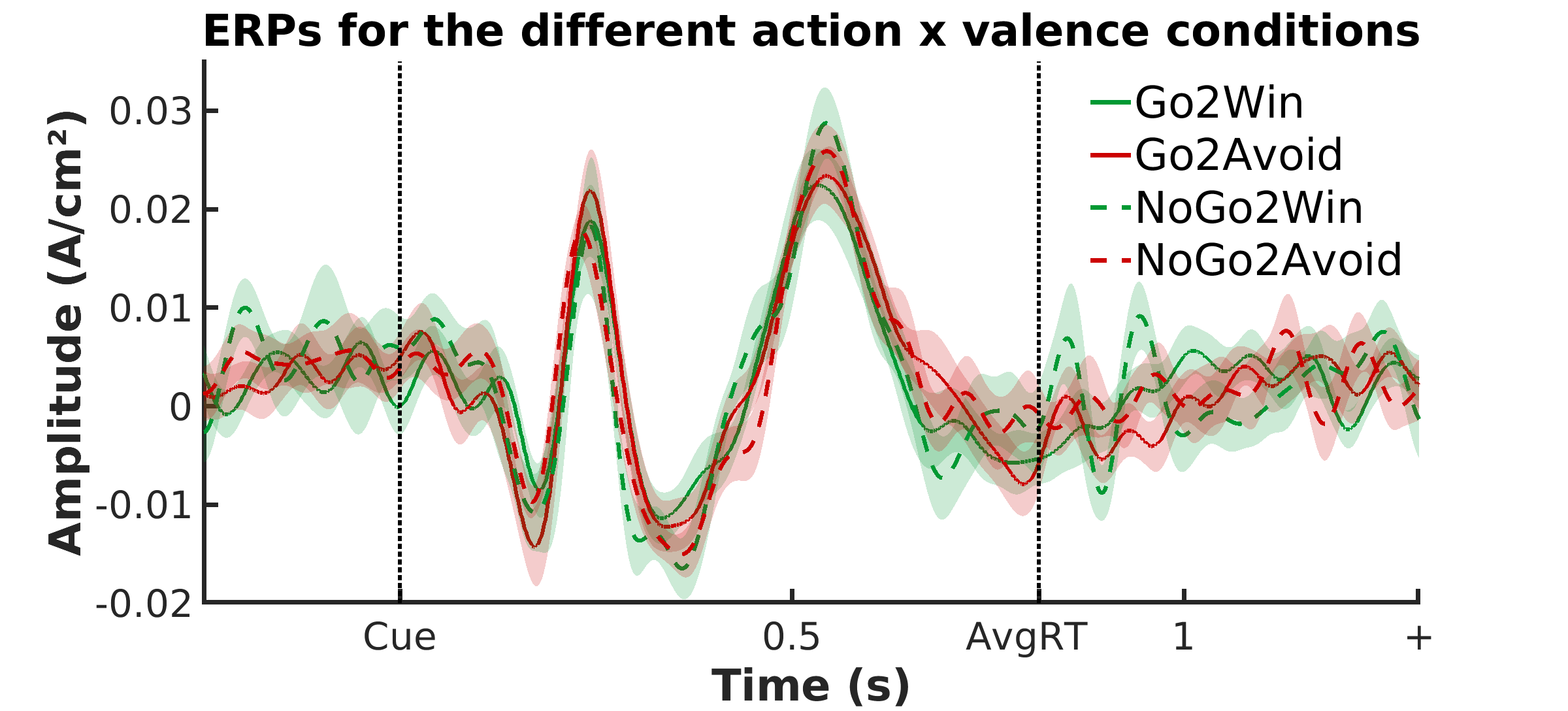** |
| --- |
| *Figure S07B*. ERPs (±SEM) as a function of cue valence and action (correct trials only) over midfrontal electrodes (Fz/FCz/Cz). There is no indication that trials with bias-incongruent (Go2Avoid and NoGo2Win) compared to bias-congruent (Go2Win and NoGo2Avoid) actions lead to systematically higher/ lower component amplitudes. |

# S08: Conflict-related alpha power after ERPs are subtracted

To test whether the observed earlier phasic alpha increase for incongruent compared to congruent conditions was attributable to evoked rather than induced activity, we removed evoked components from our data (correct trials only) by computing the average ERP for each condition per participant and subtracting it from the trial-by-trial data before performing time-frequency decomposition (Cohen & Donner, 2013). A permutation test on the alpha band yielded the same early phasic alpha increase for incongruent compared to congruent actions (*p* = .024; see Fig. S08) as reported in the main text (see Fig. 3E-F), suggesting that early alpha increase reflected induced rather than evoked activity.

| 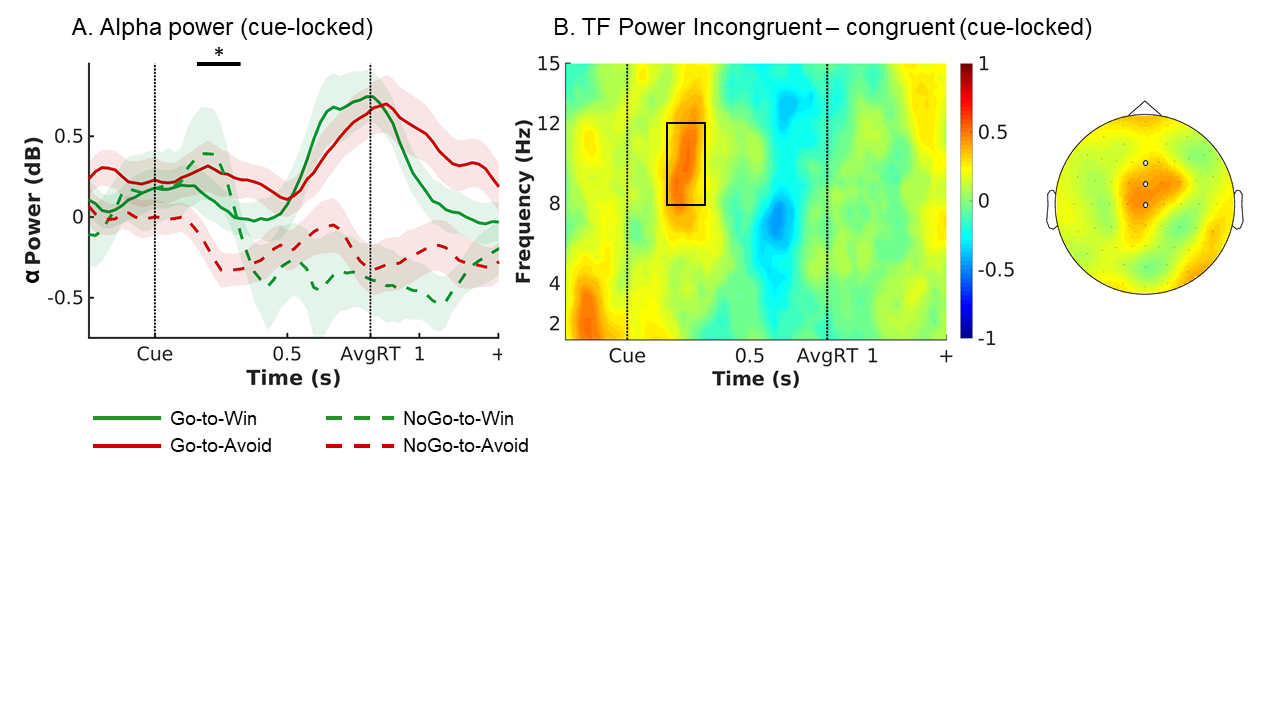 |
| --- |
| *Figure S08*. EEG alpha power with stimulus-locked ERPs subtracted. (A) Trial time course of average (±SEM) alpha power (8–13 Hz) over midfrontal electrodes (Fz/FCz/Cz) per cue condition (correct-trials only; stimulus-locked). Alpha power transiently increases for both incongruent conditions in an early time window of around 175–325 ms. The time window where the tested data shows *t*-values > 2 is indicated by the box. * *p* < 0.05. (B) Left: Time-frequency plot displaying that the transient power increase was focused on the alpha band, leaking into upper theta. Right: Topoplot of alpha power displaying that this incongruency effect was restricted to midfrontal electrodes (highlighted by white disks). |

# S09: Alpha signal as a function of cue valence, required action, and correctness

Given that we did not expect motivational conflict to be encoded in an early phasic signal in the alpha band, we conducted follow-up analyses. If this alpha signal reflected conflict detection that was causally involved in suppressing motivational biases, it should occur only when incongruent trials where met with the correct response, but be attenuated or even absent when those trials where met with an incorrect response, i.e., when participants failed to detect and/or overcome biases (Swart et al., 2018). Furthermore, the signal should occur only on incongruent trials, but not congruent trials, reflecting conflict detection mechanisms that are selectively recruited on incongruent trials rather than (possibly attentional) mechanisms improving accuracy more globally.

For this purpose, instead of global permutation tests across time and frequencies, we extracted average oscillatory power in a focal window of 175–325 ms after cue onset in the range of 8–13 Hz, averaged over midfrontal electrodes (Fz/ FCz/ Cz), for each participant, and performed repeated-measures ANOVAs with the independent variables valence (Win/ Avoid), required action (Go/ NoGo), and accuracy (correct/ incorrect; see also Swart et al., 2018).

The RM-ANOVA yielded a significant main effect of valence, *F*(1, 35) = 6.930, *p* = .013, η^2^ = 0.007, a significant two-way interaction between valence and action, *F*(1, 35) = 8.368, *p* = .006, η^2^ = 0.008, but also a significant three-way interaction between valence, action, and accuracy, *F*(1, 35) = 5.103, *p* = .03, η^2^ = 0.005 (see Fig. S09). The main effect of accuracy was not significant, *F*(1, 35) = 2.02, *p* = .164, η^2^ = 0.003, suggesting that the observed alpha effect did not reflect an (attentional) process that was overall conducive to higher accuracy. For correct trials, we found the expected two-way interaction between valence and action, *F*(1, 35) = 9.582, *p* = .004, η^2^ = 0.023, in absence of significant main effects, reflecting that alpha power was indeed higher on correct incongruent trials than correct congruent trials (simple effect: *t*(35) = 3.096, *p* = .004, *d* = 0.397). This reproduces the result of the permutation test from the main text. In contrast, for incorrect trials, we found only a significant main effect of valence, *F*(1, 35) = 8.637, *p* = .006, η^2^ = 0.011, reflecting overall higher alpha for Win than Avoid cues (simple effect: *t*(35) = 2.939, *p* = .006, *d* = 0.490), but no significant interaction between valence and action, *F*(1, 35) = 0.239, *p* = .628, η^2^ < 0.001. Incorrect incongruent trials did not lead to significantly higher alpha power than incorrect congruent trials, *t*(35) = 0.489, *p* = .628, *d* = 0.081.

These additional findings are in line with increased alpha power reflecting a conflict detection mechanism that is selectively recruited on incongruent trials on which biases are successfully overcome.

| **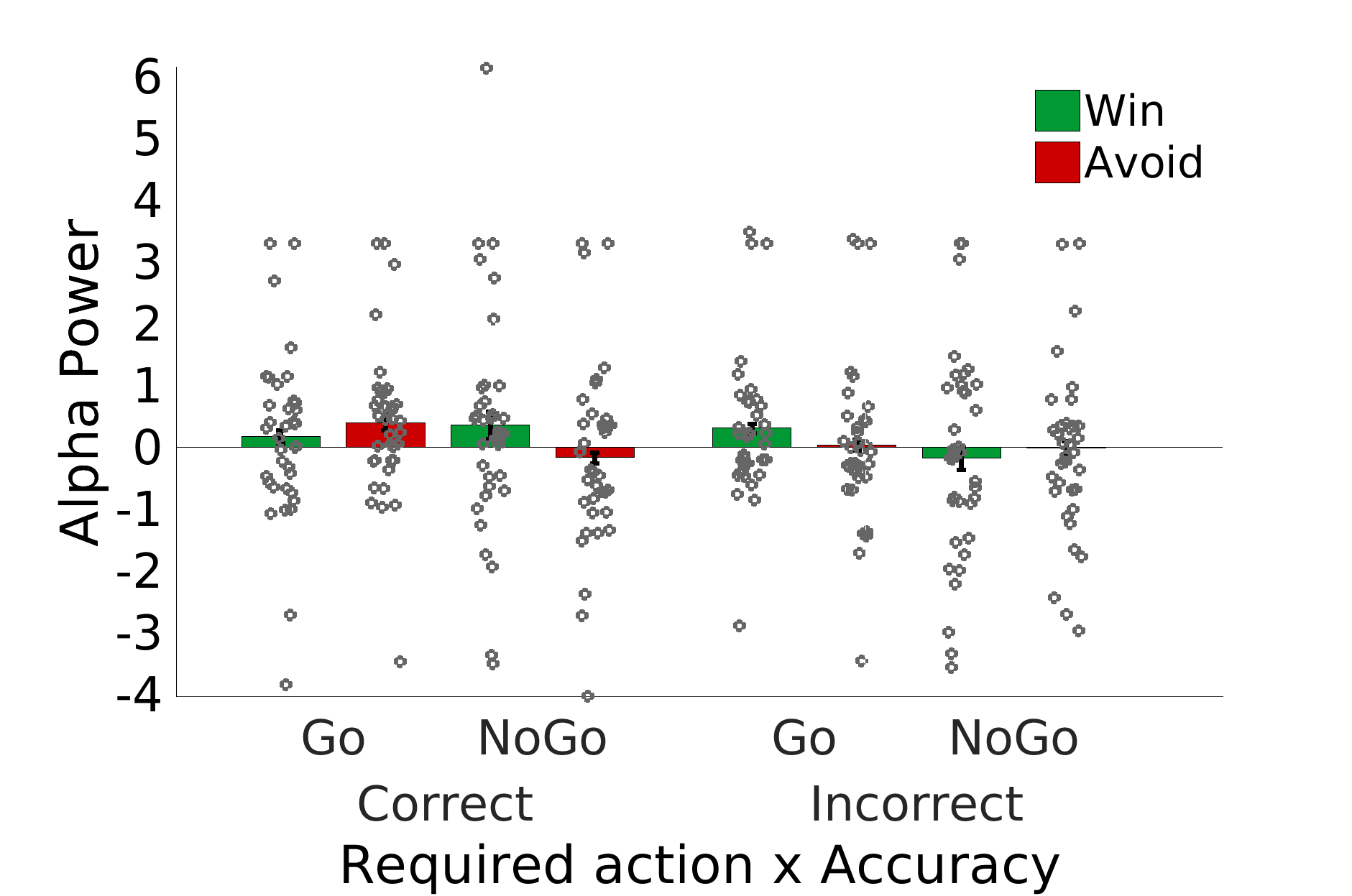** |
| --- |
| *Figure S09*. Alpha power (±SEM) as a function of cue valence, performed action, and accuracy over midfrontal electrodes (Fz/FCz/Cz). Alpha power selectively increased for correct bias-incongruent actions (correct Go2Avoid and NoGo2Win). Points are individual participant data points. |

# S10: EEG TF power as a function of action and valence across correct and incorrect trials

While EEG results reported in the main text only include correct trials in order to avoid contamination by error-related activity, fMRI results include all trials while explicitly modeling error trials with a designated regressor. To match this fMRI analysis approach, we report EEG analyses including both correct and incorrect trials, as well.

Results were highly similar to those of the correct trials only reported in the main text: Broadband power (1–15 Hz) was again significantly higher on trials with Go actions than NoGo actions (cue-locked*:* *p* = .006; response-locked: *p* = .004): This difference between Go and NoGo actions occurred as a broadband-signal from 1–15 Hz, but peaked in the theta band (Fig. S10B and D). The topographies exhibited a bimodal distribution with peaks both at frontopolar (FPz) and central (FCz, Cz, CPz) electrodes (Fig. S10B and D). As visual inspection of Fig. S10C shows, theta power increased in all conditions until 500 ms post cue onset and then bifurcated depending on the action: For NoGo actions, power decreased, while for Go actions, power kept rising and peaked at the time of the response. This resulted in higher broadband power for Go versus NoGo actions for about 575–1300 ms after cue onset (see Fig. S10C; around -150–475 ms when response-locked, see Fig. S10A). When looking at the cue-locked signal, the signal peaked earlier and higher for Go actions to Win than to Avoid cues on correct trials, but not on incorrect; hence, when testing for differences in broadband power between Win cues and Avoid cues, broadband power was not significantly different between Win and Avoid cues. This difference in latency and peak height of the ramping signal was not present in the response-locked signal, and the respective test of Win vs. Avoid cues not significant either.

| 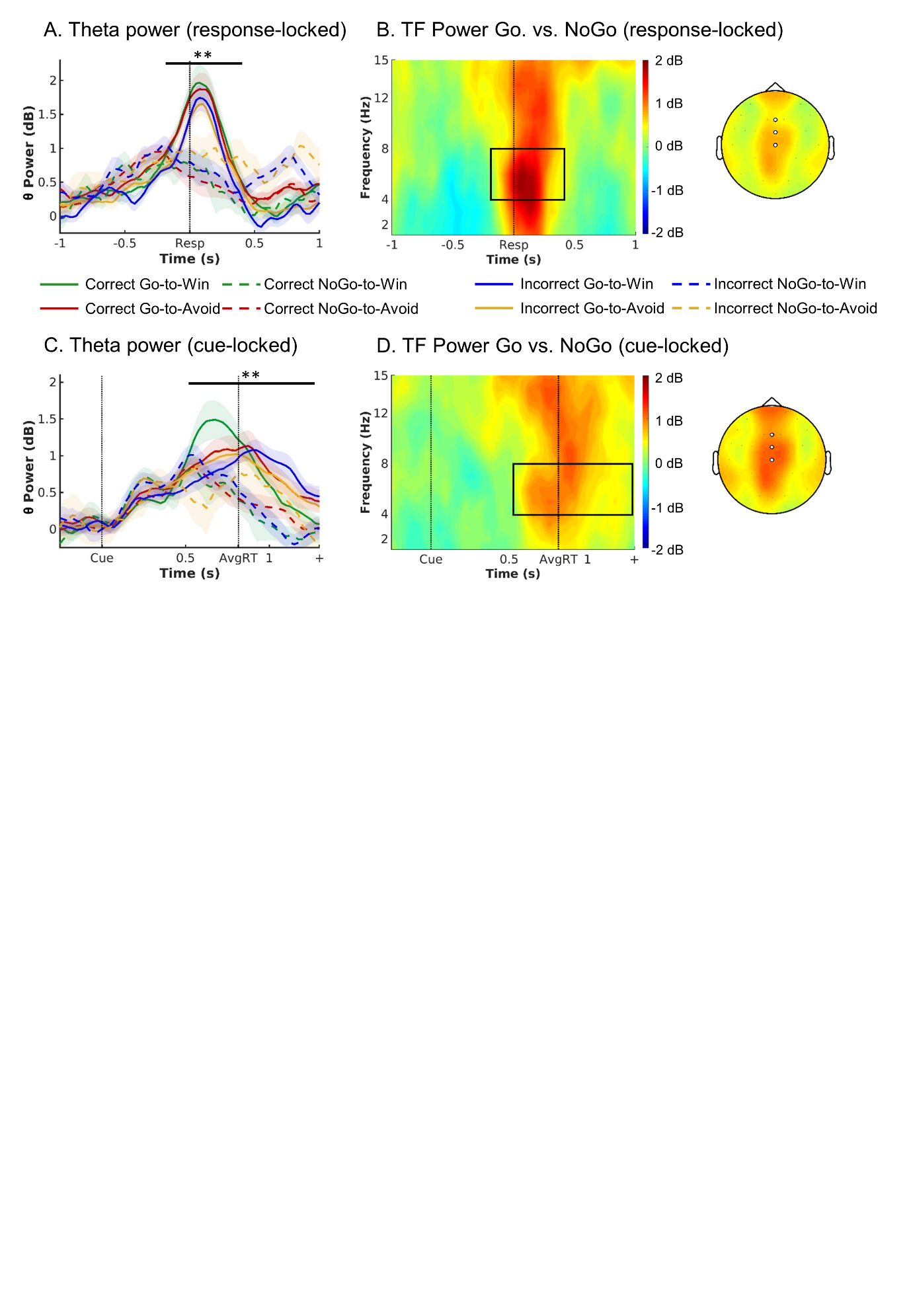 |
| --- |
| *Figure S10.* EEG time-frequency power as a function of cue valence and action for both correct and incorrect trials. (A) Response-locked within trial time course of average theta power (4–8 Hz) over midfrontal electrodes (Fz/ FCz/ Cz) per cue condition (correct-trials only). Theta increased in all conditions relative to pre-cue levels, but to a higher level for Go than NoGo trials. There were no differences in theta peak height or latency between Go2Win and Go2Avoid trials. (B) Left: Response-locked time-frequency power over midfrontal electrodes for Go minus NoGo trials. Go trials featured higher broadband TF power than NoGo trials. The broadband power increase for Go compared to NoGo trials is strongest in the theta range. Right: Topoplot for Go minus NoGo trials. The difference is strongest at FZ and FCz electrodes. (C-D) Cue-locked within trial time course and time-frequency power. Theta increased in all conditions relative to pre-cue levels, but to a higher level for Go than NoGo trials, with earlier peaks for Go2Win than Go2Avoid trials. * *p* < 0.05. ** *p* < 0.01. Shaded error bars indicate (±SEM). Box in TF plots indicates the time frequency window where *t*-values > 2. |

# S11: Plots and tests of the evidence accumulation hypothesis

Previous research has suggested theta oscillations to reflect midfrontal mechanisms that lead to an elevation of the response threshold in basal-ganglia action selection mechanisms (Cavanagh et al., 2011; Cavanagh & Frank, 2014; Cohen, 2014; Frank et al., 2015). However, an alternative interpretation of past findings could be that theta reflects the subcortical action selection process itself (Bland & Oddie, 2001; Caplan et al., 2003; DeCoteau et al., 2007a, 2007b; Womelsdorf, Vinck, Leung, & Everling, 2010). This reasoning would explain why in motor tasks, over the entire trial time course, theta oscillations strongly increase in any task condition, even in absence of conflict (Cohen & Cavanagh, 2011; Swart et al., 2018). In conflict situations warranting elevated response thresholds, this process continues beyond normal levels and evolves for an extended time period, leading to the typical “conflict-related theta” reported in the literature (Murphy, Robertson, Harty, & O’Connell, 2015).

In fact, several characteristics of the theta signal we observed resembled an accumulating evidence process as evidenced by additional tests of systematic different in peak height and latency of the signal (following O’Connell et al., 2012). In our case, features of the theta signal would be consistent with evidence selectively accumulated for making a Go action:

First, such a process should rise early (when Go is still a considered option) in all trials, but deactivate when the final response is NoGo, while it should keep rising when the final response is Go. This prediction is in line with our observations (see Fig. 3C main text).

Second, we found the theta signal to scale with reaction times, such that the signal peaked earlier on trials with earlier response times. This link would be expected when a signal causes the a response, such that the latency of the signal peak determines reaction times (O’Connell et al., 2012). To test this hypothesis in our data, we split up each participant’s trials with Go actions (correct trials only) into three equally sized bins of fast, medium, and slow reaction times (tertials), separately for Win and Avoid trials (to account for inherent differences in reaction times between Win and Avoid trials). We then computed the average stimulus-locked signal in the theta range for each bin and determined the time point between 0.3 (fastest responses) and 1.3 s (slowest possible responses) at which the signal (first) reached its peak. We then used one-tailed paired-samples *t*-tests to test whether the signal peaked earlier in bins with faster reaction times, separately for Win and Avoid trials. Overall, the signal peaked earlier for Win trials (M = 0.697, SD = 0.175) than Avoid trials (M = 0.787, SD = 0.264), *t*(35) = 2.335, *p* = 0.013, *d* = 0.39. This difference was selective for the theta band (Fig. S11B panels A-C). For Win trials, indeed, the signal peaked significantly earlier for faster than for medium reaction times, *t*(35) = 1.745, *p* = 0.045, *d* = 0.291, and significantly earlier for medium than late reaction times, *t*(35) = 2.577, *p* = 0.007, *d* = 0.430 (see Fig. S11A panel A). For Avoid trials, the signal only peaked marginally significantly earlier for faster than for medium reaction times, *t*(35) = 1.662, *p* = 0.053, *d* = 0.277, but significantly earlier for medium than late reaction times, *t*(35) = 5.220, *p* < 0.001, *d* = 0.870. Conclusions were identical when using non-parametric permutation tests instead of *t*-tests. In sum, the peak latency of the stimulus-locked theta signal scaled with reaction times, as expected for a signal triggering actions.

Third, when response-locked, differences in peak latency and height between cue valence conditions and reaction time bins disappeared, in line the assumption of a fixed threshold that evidence must reach in order to trigger action release (O’Connell et al., 2012). To investigate systematic differences in peak latency, we computed the average response-locked signal in the theta range for each bin for each participant and determined the time point between 0.5 s before and 0.5 s after the response at which the signal (first) reached its peak. We again compared bins within each valence condition using two-tailed *t*-tests. For Win trials, there were no significant differences in peak latency between fast and medium reaction times, *t*(35) = -0.784, *p* = 0.438, *d* = -0.131, medium and slow reaction times, *t*(35) = 0.896, *p* = 0.376, *d* = 0.149, or fast and slow reaction times, *t*(35) = 0.135, *p* = 0.894, *d* = 0.023 (see Fig. S11A panel B). Similarly for Avoid trials, there were no significant differences in peak latency between fast and medium reaction times, *t*(35) = 0.014, *p* = 0.988, d = 0.002, medium and slow reaction times, *t*(35) = 0.347, *p* = 0.730, *d* = 0.058, or fast and slow reaction times, *t*(35) = 0.245, *p* = 0.808, *d* = 0.041. Conclusions were identical when using non-parametric permutation tests instead of *t*-tests.

To investigate significant differences in peak height, we extracted the height of the theta signal at the peak latency within each bin for each participant. We again compared bins within each valence condition using two-tailed *t*-tests. For Win trials, were no significant differences in peak height between fast and medium reaction times, *t*(35) = -1.138, *p* = 0.264, *d* = -0.190, medium and slow reaction times, *t*(35) = 1.003, *p* = 0.322, *d* = 0.167, or fast and slow reaction times, *t*(35) = 0.206, *p* = 0.838, *d* = 0.034 (see Fig. S11A panel B). Similarly for Avoid trials, there were no significant differences in peak height between fast and medium reaction times, *t*(35) = -0.017, *p* = 0.986, *d* = -0.003, medium and slow reaction times, *t*(35) = 0.195, *p* = 0.846, *d* = 0.033, or fast and slow reaction times, *t*(35) = 0.195, *p* = 0. 846, *d* = 0.033. Conclusions were identical when using non-parametric permutation tests instead of *t*-tests. Taken together, there were no significant differences in peak latency and height between different reaction times, in line with a fixed response threshold independent of response time or cue valence.

Fourth, previous research on EEG correlates of evidence accumulation in perceptual decision making has found that incorrect responses were elicited at systematically lower thresholds than correct responses (O’Connell et al., 2012), suggesting that trial-by-trial variation in the response threshold can cause erroneous action releases. To test this hypothesis in our data, we computed the response-locked theta signal separately for correct and incorrect Go actions on Win and Avoid trials, and then computed the peak height for each participant. We used one-tailed *t*-tests to test whether peak height was lower for incorrect than correct trials. This was indeed the case both on Win trials, *t*(35) = 2.558, *p* = 0.008, *d* = 0.426, and Avoid trials, *t*(35) = 2.729, *p* = 0.005, *d* = 0.455 (Fig. S11A panel C). As an alternative, we performed a cluster-based permutation test contrasting correct and incorrect responses. Both were indeed significantly different, p = 0.047, most dominantly from 125 ms before until 25 ms after around responses. In conclusion, we found evidence in line with the hypothesis that false-positive action releases occur at a systematically lower response threshold than true-positive action releases.

Fifth, our interpretation of theta as reflecting evidence accumulation is in line with previous research that has found perceptual and value-based evidence to be reflected in the theta band (Hunt et al., 2012; van Vugt, Simen, Nystrom, Holmes, & Cohen, 2012). Also, a recent study observed both perceptual and value-based evidence encoded in the gamma band in topographies very similar to the one we found, with peaks in both frontopolar and centroparietal electrodes (Polanía, Krajbich, Grueschow, & Ruff, 2014). It is possible that feedforward activity encoded in the gamma band is nested in lower-frequency theta cycles reflecting top-down integration (Canolty et al., 2006; Landau, Schreyer, van Pelt, & Fries, 2015; Maris, van Vugt, & Kahana, 2011). In conclusion, our results are in line with theta power reflecting an evidence accumulation process for deciding whether to perform an active Go response.

| **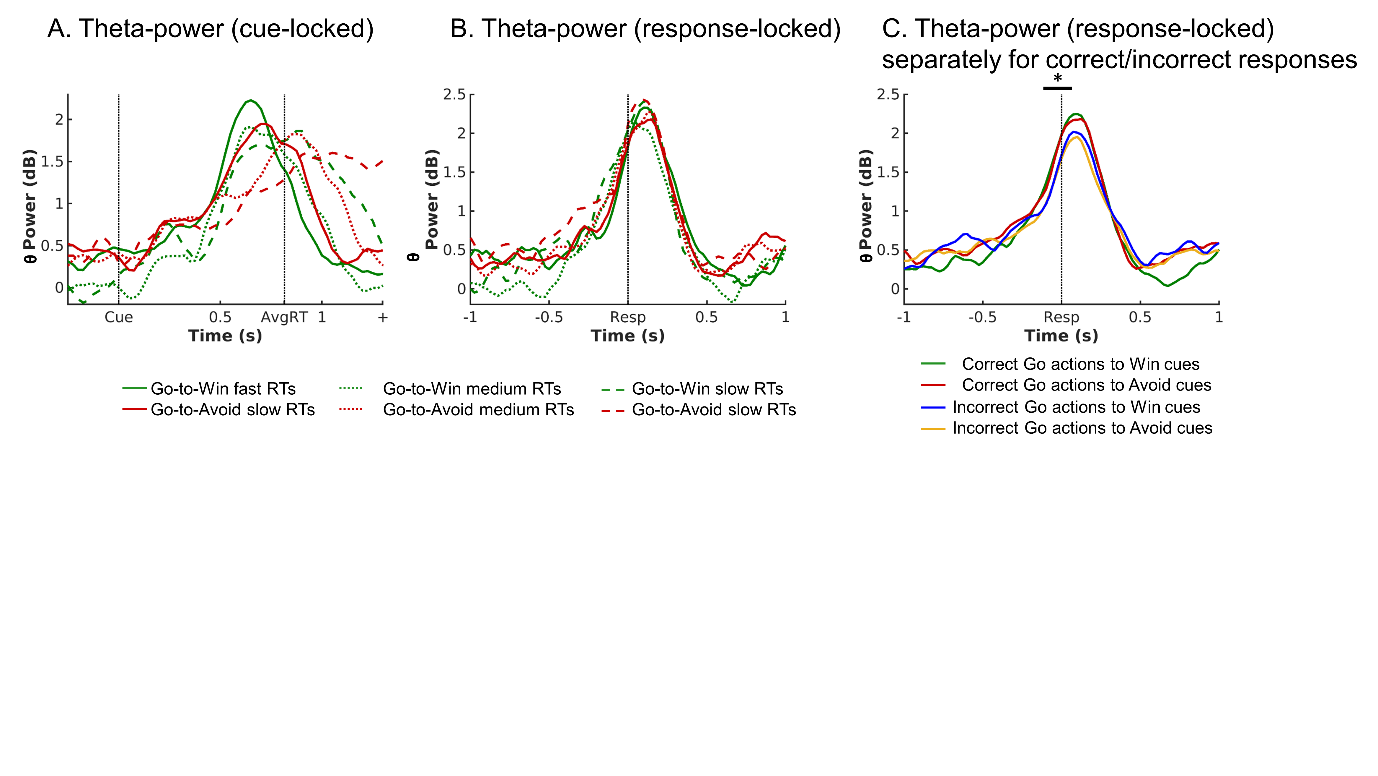** |
| --- |
| *Figure S11A*. Plots displaying features of the theta signal akin to an evidence accumulation process for active Go responses. (A) The stimulus-locked theta signal split into fast, medium, and slow reaction time bins separately for Win and Avoid trials. The signal peaks systematically earlier for earlier reaction times. (B) The same signal response-locked. Differences in peak height and latency between reaction time bins are absent. (C) Correct and incorrect Go actions separately for Win and Avoid trials. The theta peaks at significantly lower levels for incorrect compared to correct actions. |

| **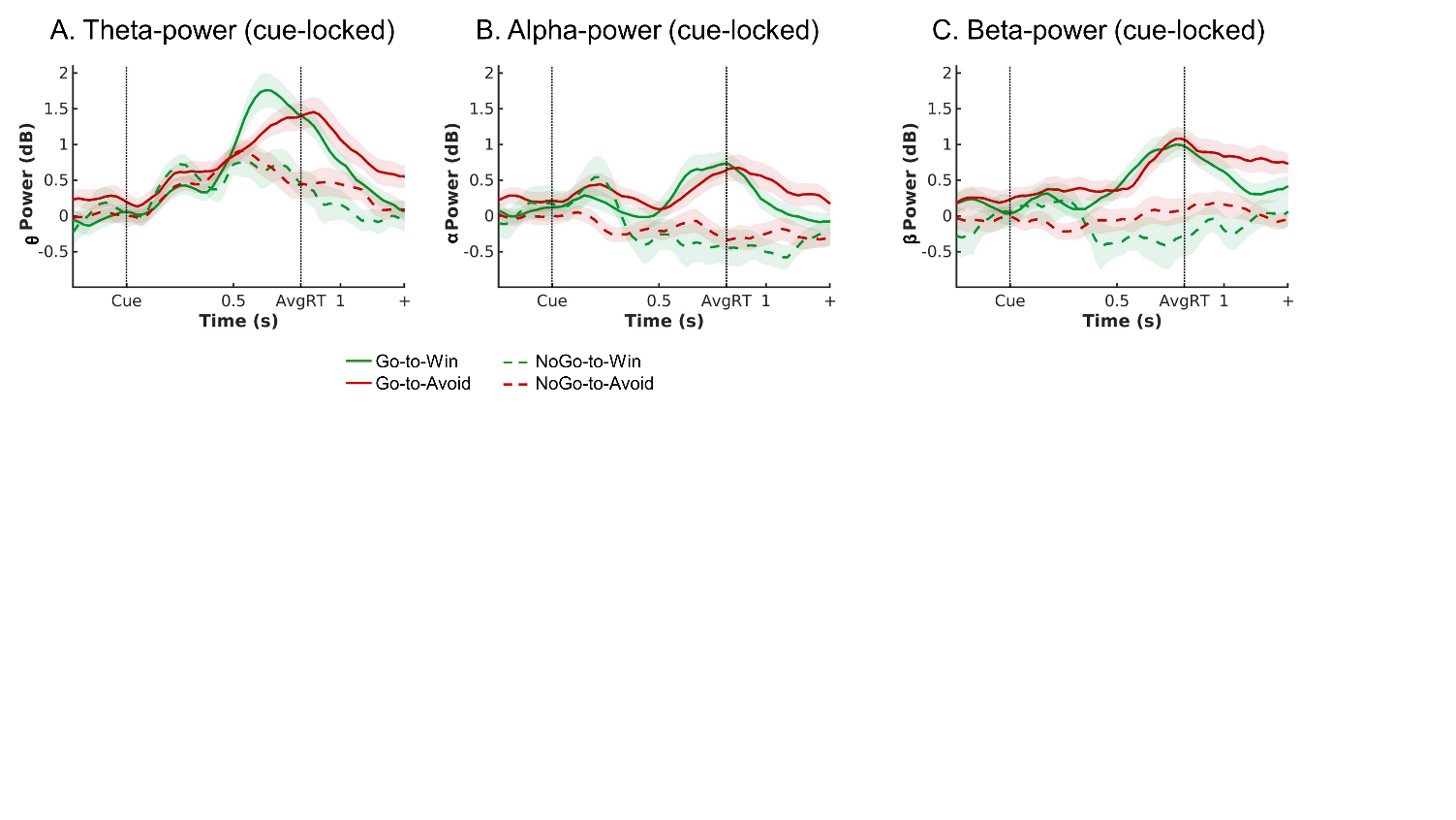** |
| --- |
| *Figure S11B*. Plots displaying theta (A), alpha (B), and beta (C) power over midfrontal channels (Fz/ FCz/ Cz) split per cue-valence and performed action (correct trials only to avoid contamination error processing). Signal rise is strongest in the theta band. Also, the positively accelerating shape typically observed in evidence accumulation signals (Donner, Siegel, Fries, & Engel, 2009; O’Connell et al., 2012) is only observed in the theta band. Finally, differences in peak latency between Win and Avoid cues (in line with differences in reaction times between conditions) only arise in the theta band. |

# S12: Correlation of EEG power with head motion

Recently, Fellner and colleagues (2016) reported that lower-frequency oscillations in simultaneous EEG-fMRI studies were affected by head motion: Realignment parameters strongly correlating with time-frequency power. These authors recorded simultaneous EEG and fMRI during encoding and retrieval phases of a memory task. They found difference in theta oscillations for remembered vs. forgotten items that had the opposite sign compared to when the same task was performed with EEG outside the MR scanner. Also, they found overall lower-frequency oscillations, especially in the theta range, to be strongly correlated with a summary measure of the six realignment parameters, casting doubt on the neural origin of theta effects measured in simultaneous EEG-fMRI recordings.

We leveraged our approach of fMRI-inspired EEG analysis approach (see main text) by computing the same summary statistic as Fellner and colleagues (2016) based on the six realignment parameters for each volume, upsampling this signal to a TR of 0.140 s, and then downsampling the signal again into epochs of 2 s length relative to trial onset, yielding an indicator of overall head motion during each trial.

First, similar to Fellner and colleagues (2016), we compared the head motion between trials with Go actions and trials with NoGo trials by computing the average head motion summary statistic for such trials for each participant and then performing a two-tailed paired-samples *t*-test. There was no significant difference in head motion between trials with Go and trials with NoGo actions, *t*(35) = 1.614, *p* = 0.116, *d* = 0.269. Similarly, there was no significant difference in head motion between Win and Avoid trials, *t*(35) = -0.467, *p* = 0.643, *d* = -0.078, nor between congruent and incongruent trials, *t*(35) = -0.304, *p* = 0.763, *d* = -0.051. These results suggest that head motion did not differ between experimental conditions.

Next, we used the head motion summary statistic as a trial-by-trial predictor of time-frequency power in multiple regression, similarly to the BOLD signal extracted from neural regions. When head motion was used as a sole regressor, we indeed observed a significant positive correlation with theta/ delta power (*p* = 0.039). However, this correlation was not spread out in time and frequency space as in Fellner and colleagues (2016), but instead focused on theta/ delta power around 0.9–1.3 s. after cue onset—i.e., after the average response time (see Fig. S12A). When entering BOLD signal from the selected regions as additional regressors, this pattern remained similar but became non-significant (*p* = .089).

Given that participants were instructed to perform Go actions only while the respective cue was visible (0–1.3 s after cue onset), we restricted our analyses of task-related neural signals to this period. However, head motion-related signals might occur even after this period. When performing fMRI-inspired EEG analyses on a window of 0–2 s after cue onset, we indeed observed a strong correlation (*p* = 0.005) of head motion with broadband time-frequency power after cue offset (after 1.3 s, see Fig. S12B; especially so when including the five participants that were otherwise excluded from the fMRI-informed EEG analyses; see Fig. S12C). This finding suggests that head-motion (and associated artifacts) might predominantly occur during the inter-stimulus interval when participants have performed any cue-related action and wait for the outcome. Note that the trials in Fellner and colleagues (2016) were much longer (3 s) than in our paradigm. Hence, head motion might be a particular problem in EEG-fMRI studies with paradigms featuring long trial durations, unlike the cue presentation phase in our paradigm.

Correlations of time-frequency power with BOLD and task factors remained significant even when head motion was included in the regression: First, we still observed a significant correlation of striatal BOLD with late theta/ delta power 0.5–1.0 s after cue onset (*p* = .045), suggesting that striatal BOLD predicts theta power independently of any head motion-related signals (see Fig. S12D). Second, the correlations of left (*p* = .008) and right (*p* = .027) motor cortex (see Supplementary Material S15) and vmPFC (*p* = .035) BOLD with time-frequency power remained significant. Third, inspecting the beta-map of the additional regressor Go vs. NoGo responses (coded as 1 and 0, regressor demeaned), which we included in all these analyses by default, revealed correlations with broadband signal around the time of responses (*p* = .026), a pattern very similar to the EEG-only analyses (see Fig. S12E; compare to Fig. 3D in the main text). This finding suggests that the broadband signal associated with Go vs. NoGo responses in EEG-only analyses is not reducible to head motion artifacts either.

Finally, when using the trial-by-trial theta power as a regressor in an fMRI GLM (see Supplementary Material S17), we observed theta correlates in action-related regions such as ACC, motor cortices, opercula, putamen, and cerebellum, unlike Fellner and colleagues (2016) who observed correlates mostly in regions of the default-mode network. We thus conclude that the theta effects we observed constitute task-related neural signals rather than the head-motion related artifacts described by Fellner and colleagues (2016).

| 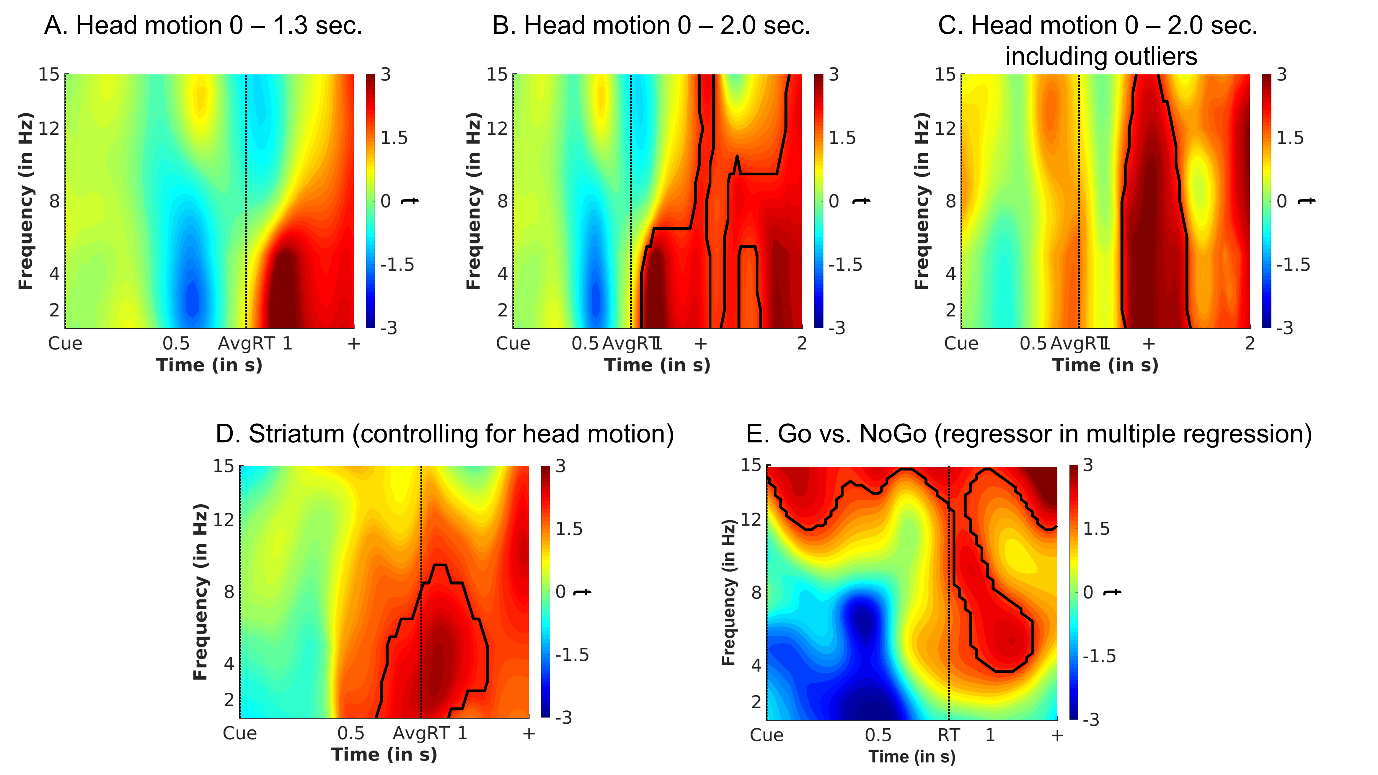 |
| --- |
| *Figure S12.* Time-frequency correlates of head motion and task effects when controlled for head motion. (A) Head motion was quantified via relative displacement as a summary statistic of the volume-by-volume realignment parameters, which was first upsampled, then downsampled into a single value per trial, and used to predict average time-frequency power over midfrontal electrodes. Head motion correlated (though not significantly) with theta/ delta power around 1 sec. after stimulus onset. (B) The same analyses on a time window of 0–2.0 revealed that head motion predominantly correlated with broadband time-frequency power after cue offset. (C) Same plot as (B), but with the four participants included that are typically excluded due to out-of-range regression weights and strong head motion. (D) The correlation of striatal BOLD with theta/ delta power around the time of responses remained unaltered when entering head motion as an additional regressor into the model. (E) Using Go vs. NoGo responses (coded as 1 and 0, demeaned) as an additional regressor yielded again an increase in broadband time-frequency power for Go compared to NoGo responses (see main text Figure 3D), even when entering head motion as an additional regressor into the model. Areas surrounded by a black edge indicate clusters of \|*t*\| > 2 with *p* < .05 (cluster-corrected). |

# S13: Increase in midfrontal time-frequency power relative to baseline for each cue valence x performed action pairing

| **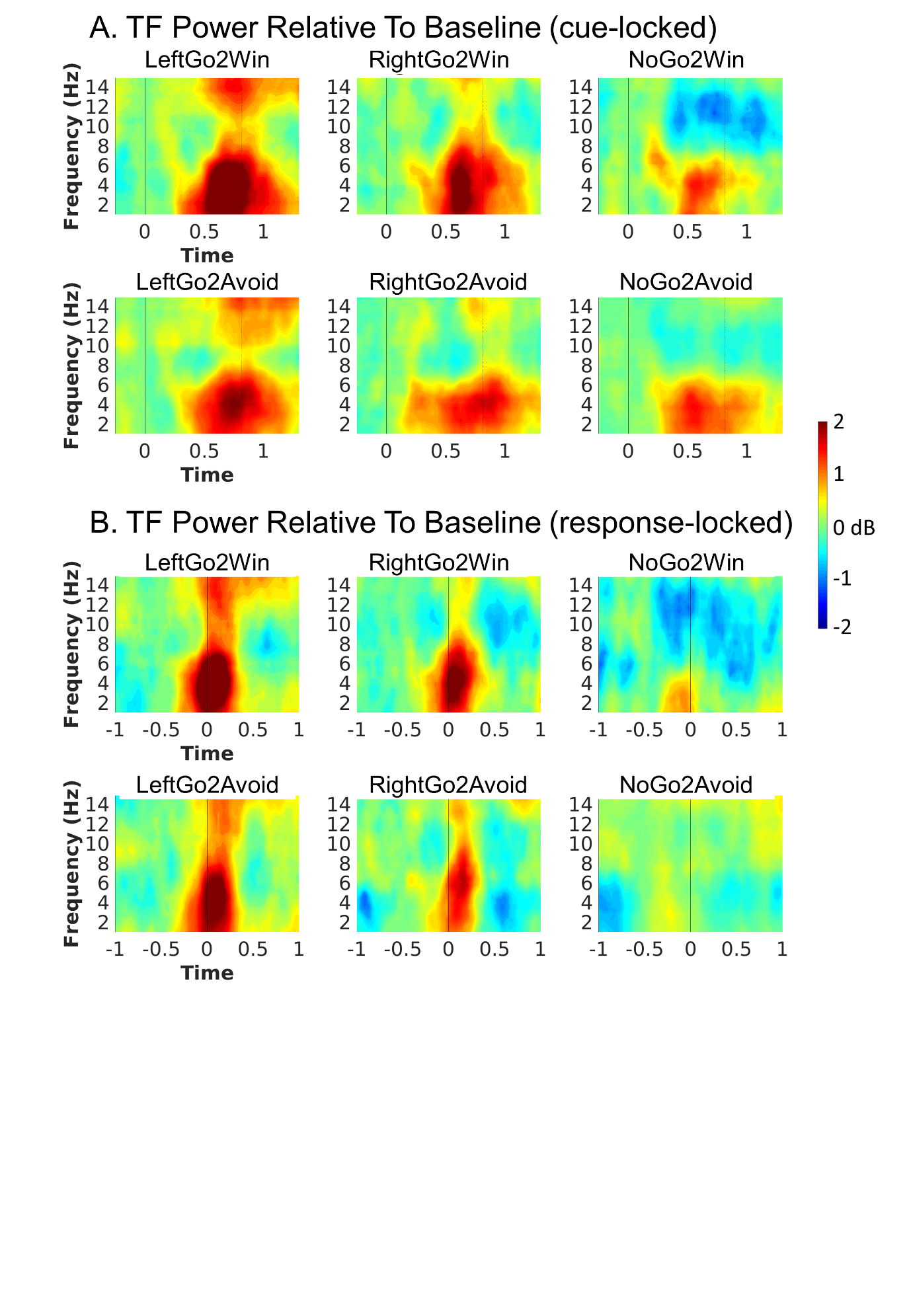** |
| --- |
| *Figure S13*. Average time-frequency power over midfrontal electrodes (Fz/FCz/Cz) relative to baseline (mean signal -250–50 ms before cue onset), split up per cue valence (rows) and performed action (columns), both stimulus-locked (A) and response-locked (B). For stimulus-locked plots, vertical solid lines indicate cue onset, dashed vertical lines average response time. For response-locked plots, vertical solid lines indicate response time (i.e., 0 s). Time-frequency power in the delta/ theta range increases in every valence-action pairing, also for NoGo actions, which rules out that this signal is a mere motion artifact. |

# S14: Theta and beta power for left vs. right hand responses

The strong association of broadband power with motor activity opened the possibility that this signal was potentially an artifact of EEG acquisition (e.g. head movement in the scanner) rather than a neural signal (Fellner et al., 2016). If the signal constituted an artifact, one would expect it to be symmetrical for both hands. One the other hand, if it was a neural signal reflecting the level of evidence accumulated before initiating a response, one might expect the signal to be sensitive to differences in evidence thresholds between hands. Given that all our participants were right-handed, one might expect that responses of the left (non-dominant) hand were less easily initiated and required a higher level of evidence to be selected than responses of the right (dominant) hand. A broadband permutation test (stimulus-locked: *p* = .020; response-locked: *p* = .006) indicated that power in the theta band (around -250–25 ms relative to responses, see Fig. S14) and in the beta-band (around -150–675 ms relative to responses (see Fig. S14B and D) was in fact higher for left-hand than right-hand responses.

This modulation of the theta signal by handedness corroborates the interpretation that the observed theta synchronization is of neural origin. It might reflect a bias towards right-hand responses, such that left-hand responses require a higher level of evidence to be initiated than right-hand responses. Such a right-hand bias might also explain why synchronization in the beta band was higher for left than right hand responses: Given that beta synchronization is typically found for motor inhibition (Wessel et al., 2016; Wessel, Waller, & Greenlee, 2019), higher beta on trials with left-hand responses might reflect that the right hand needed to be actively suppressed on these trials (see also Supplementary Material S15). In fact, we also observed that left-hand responses (*M* = 0.772) were overall slower than right hand responses (*M* = 0.745), χ^2^(1) = 6.709, *p* = .010, which further corroborates the interpretation that left-hand responses might have been harder to perform than right-hand responses.

| 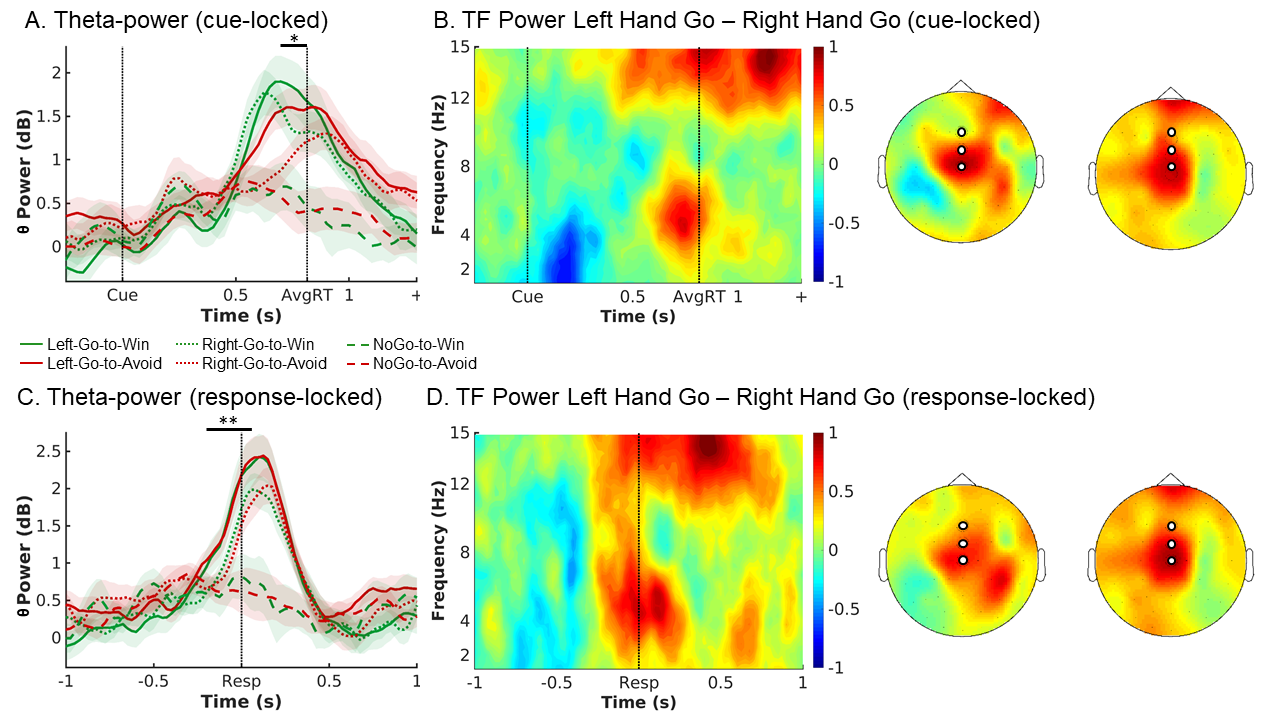 |
| --- |
| *Figure S14*. Theta and beta power split up for left vs. right hand responses. (A) Trial time course of average (±SEM) theta power (4–8 Hz) over midfrontal electrodes (Fz/FCz/Cz) per split up for left- and right-hand responses (correct-trials only; stimulus-locked). Theta increased to a higher extend for left-hand than right-hand responses. * *p* < 0.05. (B) Left: Time-frequency power over midfrontal electrodes for left-hand minus right-hand responses trials. Left-hand responses were associated with high theta and beta power compared to right-hand responses. Right: Topoplot for left-hand minus right-hand responses in the theta (left) and beta (right) range. (C-D) Same data when response-locked. Differences in theta peak latency between Go2Win and Go2Avoid trials disappear. ** *p* < 0.01. |
|  |

# S15: Supplementary fMRI-inspired EEG results in time-frequency space

In addition to EEG correlates of BOLD signal in the striatum, ACC and vmPFC (see main text), we also observed correlates for BOLD in left and right motor cortex. Both left motor cortex (two separate clusters, both ­*p* = .030 and *p* = .030, cluster-corrected) and right motor cortex (*p* = .001 cluster-corrected) exhibited overlapping, but oppositely signed correlates in the alpha/beta band, with left motor cortex correlating negatively with midfrontal beta power (around 12–15 Hz, 0.6–1.3 s, Fig. S15B), while right motor cortex correlated positively with alpha/beta power (around 10–15 Hz, 0.6–1.3 s, Fig. S15C). These findings mirror the observation of higher theta and beta power for left hand (i.e., right motor cortex) compared to right hand (i.e., left motor cortex) responses (see Supplementary Material S09), again suggesting that executing a left-hand response might have required an active suppression (associated with increased beta power) of the right hand. These results corroborate that theta power does not reflect motor preparation/ execution signals from the motor cortices, but signals from distinct regions. Furthermore, these associations replicate numerous intracranial and source-localization studies (Salmelin, Forss, Knuutila, & Hari, 1995; Salmelin, Hämäläinen, Kajola, & Hari, 1995; Sanes & Donoghue, 1993; Stolk et al., 2019) and previous EEG-fMRI studies (Jurkiewicz, Gaetz, Bostan, & Cheyne, 2006; Ritter, Moosmann, & Villringer, 2009) reporting beta oscillations in motor cortices. The presence of this well-established BOLD-EEG association corroborates the robustness of the data and analysis.

We performed a range of follow-up analyses to check for the robust of our results. We reached similar results and identical conclusions when a) performing regressions with each region as the sole predictor, b) including a summary measure of the realignment parameters as a proxy for head motion into the regression (Fellner et al., 2016), and c) when fitting HRFs for all trials of a certain block within a single GLM instead of separately for each trial.

We lastly aimed to test whether distinct striatal subregions with opposite valence coding, i.e., left putamen (Win > Avoid) and bilateral medial caudate (Avoid > Win), showed distinct time-frequency correlates. When using BOLD from those subregions instead of overall striatal BOLD as regressors, left putamen BOLD did not exhibit a significant association with time-frequency power (*p* = .218), while medial caudate BOLD did significantly correlate with delta/theta power around 825–1,2500 ms post-stimulus (*p* = .011). The cluster of significant correlations observed for medial caudate was highly similar to the cluster observed as a correlate of the entire striatum. Descriptively, both ROIs showed clusters of positive correlations with theta/ delta power around the time of responses, and slightly earlier so for the left putamen than for medial caudate. This finding would be in line with the idea of left putamen more strongly driving Go responses on Win trials, which showed shorter RTs, and medial caudate rather driving Go responses on Avoid trials, which showed longer RTs. However, as clusters associated with those regions were small and permutation tests not significant, this descriptive finding should be interpreted with caution.

| 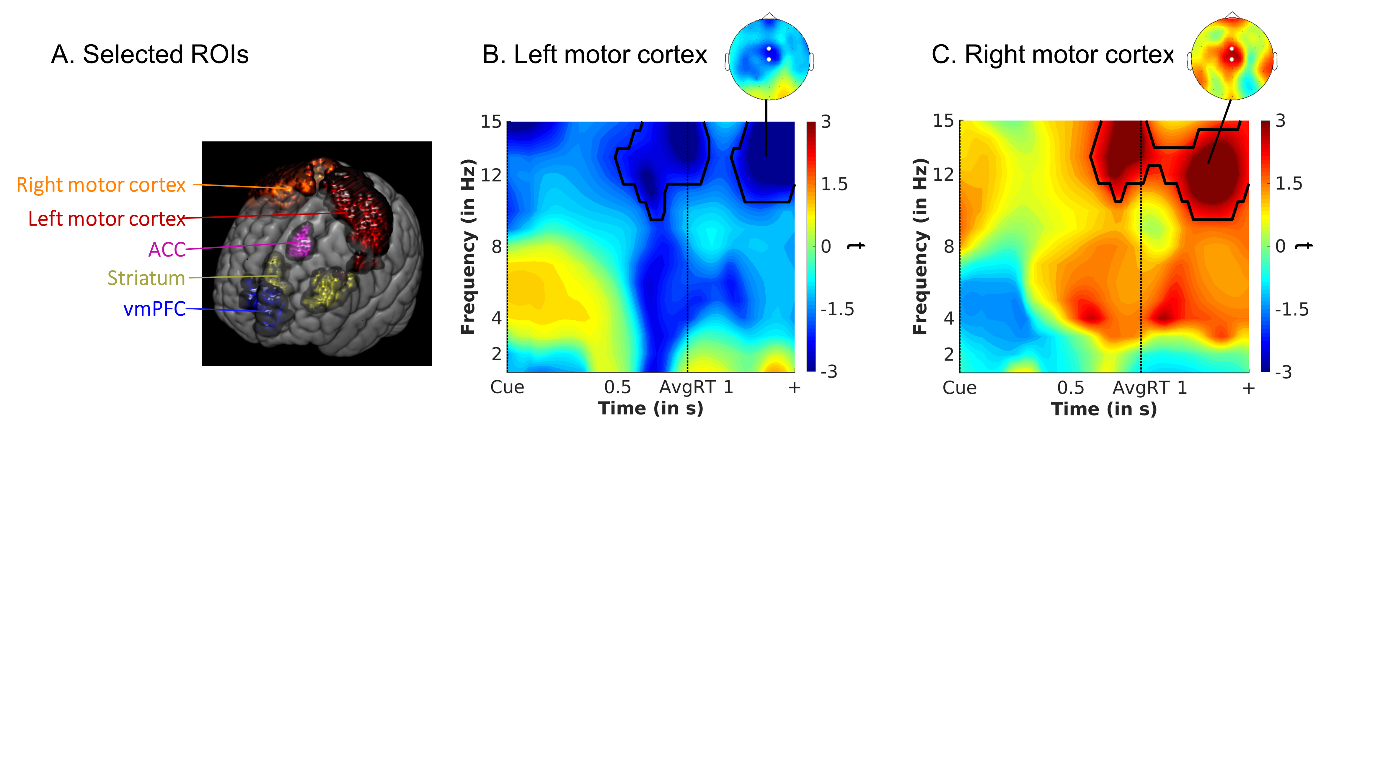 |
| --- |
| *Figure S15.* (A) Selected ROIs from which trial-by-trial HRF amplitudes were used as predictors in a multiple regression of midfrontal EEG time-frequency power. (B-C) Unique temporal correlation of BOLD signal in (B) left and (C) right motor cortex to average EEG time-frequency power over midfrontal electrodes (FCz/Cz). Group-level *t*-maps display the modulation of the EEG time-frequency power by trial-by-trial BOLD signal in the selected ROIs. Midfrontal beta power correlates negatively with BOLD in left motor cortex (more active for right hand responses), but positively with BOLD in right motor cortex (more active for left hand responses), putatively indexing response conflict and inhibitory processes when left hand responses were executed. Areas surrounded by a black edge indicate clusters of \|*t*\| > 2 with *p* < .05 (cluster-corrected). Topoplots indicate the topography of the respective cluster. |
|  |
|  |

# S16: Supplementary fMRI-inspired EEG results in time space (ERPs)

Given that the time-frequency correlate of trial-by-trial vmPFC BOLD occurred very early after cue onset and was extended in frequency space, we hypothesized that vmPFC BOLD might be correlated with evoked rather than induced activity, which, when analyzed in time-frequency space, smeared across frequencies. We used the same approach for fMRI-informed EEG analyses as reported in the main text, but with the voltage signal (time-domain) instead of time-frequency power as dependent variable. We again used BOLD signal from striatum, ACC, left and right motor cortex, and vmPFC as simultaneous predictors in one single multiple regression.

When restricting analyses to midfrontal electrodes (FCz/ Cz), we found no significant modulation of EEG voltage by vmPFC BOLD (*p* = .260; see Fig. S16A and C). However, when considering a broader frontal ROI (F1/F3/FCz/FC1/FC3/ Cz/C1/C3), vmPFC appeared to attenuate the amplitude of the P2 component over left frontal electrodes (two clusters above threshold: *p* = .021 around 213–269 ms; *p* = .003 around 349 – 410 ms; see Fig. S16B and D). The topography of EEG voltage modulation did not exactly match with the topography of the time-frequency power modulation, but was rather restricted to left frontal electrodes (see Fig. S16C and E). Interestingly, these electrodes also showed the peak modulation of the P2 by Go compared to NoGo actions (see Supplementary Material S07). In conclusion, we found inconclusive evidence regarding whether broadband power decreases associated with vmPFC BOLD were reducible to evoked activity (modulation of the P2) or not.

| **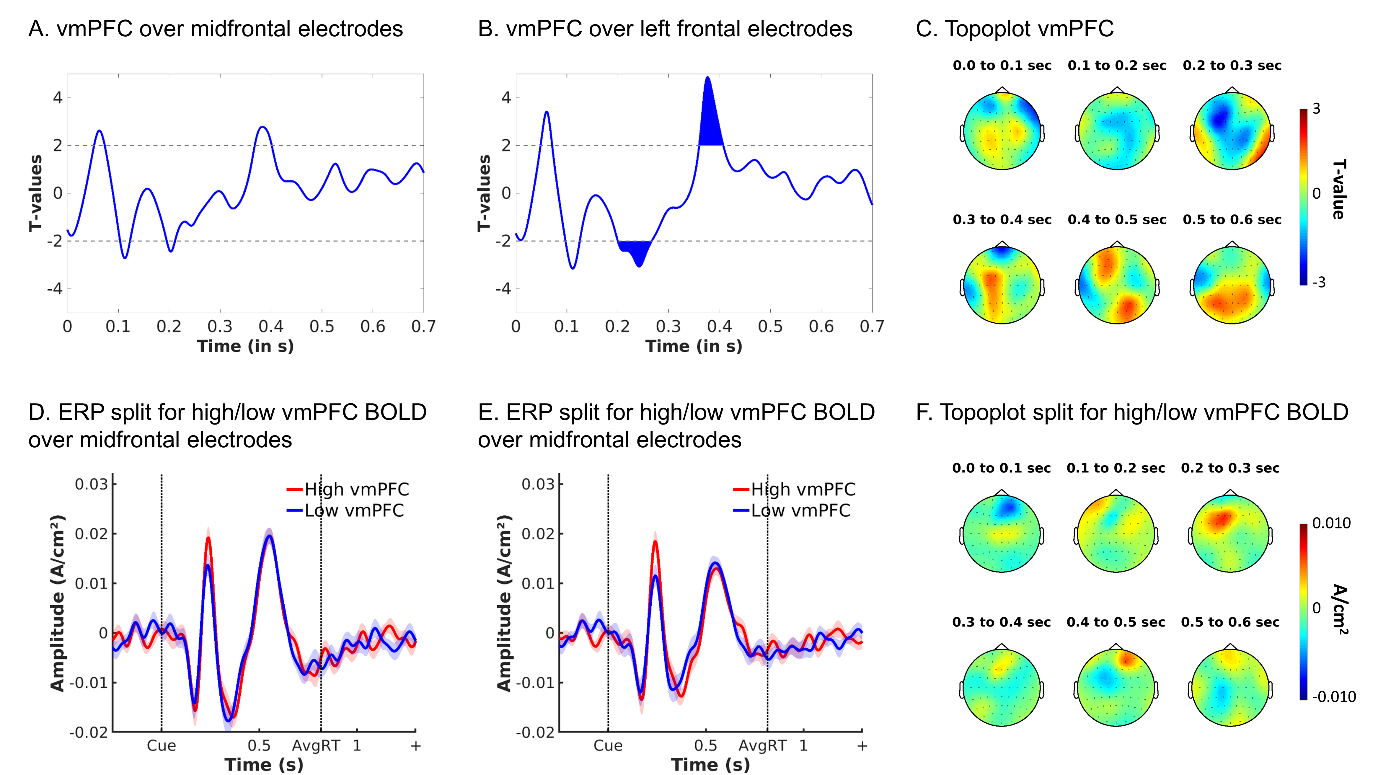** |
| --- |
| *Figure S16*. Modulation of EEG voltage by vmPFC BOLD signal. (A) Average EEG voltage over midfrontal electrodes (FCz/ Cz) was not significantly modulated by vmPFC BOLD, while (B) EEG voltage over left frontal electrodes (F1/ F3/ FCz/ FC1/ FC3/ FC5/ Cz/ C1/ C3) was. Filled areas indicate clusters of \|*t*\| > 2 with *p* < .05 (cluster-corrected). (C) Topoplots displaying *t*-values over the entire scalp in steps of 100 ms from 0 to 800 ms. The strongest modulation of frontal EEG voltage by vmPFC BOLD occurred over left frontal electrodes. This pattern does not fully match the topography of broadband power decreased associated with vmPFC BOLD (see Fig. 4C main text). (D) For plotting purposes, we sorted trials according to the trial-by-trial HRF amplitude in the vmPFC ROI and plotted the 33% of trials with highest vmPFC BOLD signal vs. the 33% trials with lowest vmPFC BOLD signal. This contrast indicates no strong difference a midfrontal electrodes (FCz/ Cz), but (E) does indicate an attenuation of the P2 component through high vmPFC BOLD over left frontal electrodes (F1/ F3/ FCz/ FC1/ FC3/ FC5/ Cz/ C1/ C3). (F) Topoplots displaying voltage for high vmPFC BOLD minus low vmPFC BOLD trials over the entire scalp in steps of 100 ms from 0 to 800 ms. The strongest modulation of frontal EEG voltage by vmPFC BOLD occurred over left frontal electrodes. |

# S17: EEG-informed fMRI analyses

For the EEG-inspired fMRI analyses, we added trial-by-trial summary measures of conflict-related alpha power and action-related theta power to our GLM. These measures were created by using the 3-D (time-frequency-channel) *t*-map obtained when contrasting incongruent vs. congruent actions (Mask 1; stimulus-locked) and Go vs. NoGo actions (Mask 2; response-locked) over midfrontal channels (Fz/ FCz/ Cz) as a linear filter. We extracted those maps and retained all voxels with *t* > 2. We did not enforce strict frequency band cutoffs, but rather extracted the entire cluster of *t*-values above threshold. Restricting the action contrast *t*-map to the theta range or using the stimulus-locked rather than the response-locked map led to highly similar results and identical conclusions. These masks were applied to the trial-by-trial time-frequency data to create weighted summary measures of the average power in the identified clusters in each trial. Both resultant time series correlated only weakly (mean correlation across participants: *r* = .105). They were entered as parametric modulators on top of the task regressors as described above, with each regressor entering a separate parametric contrast.

The EEG alpha regressor correlated significantly negatively with BOLD in two clusters in left middle frontal gyrus/ lateral frontal pole (z_max_ = 3.99, p = 0.000506, xyz = [-38 34 32]) and in right supramarginal gyrus (z_max_ = 4.45, p = 0.00619, xyz = [54 -42 38]). As Fig. S17A indicates, sub-threshold, the same areas in the respective other hemisphere also correlated negatively with trial-by-trial alpha, as did extensive areas in medial parietal/ occipital cortex. Overall, midfrontal alpha appeared to correlate negatively with extended areas that were part of the fronto-parietal and dorsal attention resting-state networks. Notably, no region correlated positively with midfrontal alpha.

The EEG theta regressor correlated significantly positively with BOLD in pre-SMA/ ACC/ left precentral and postcentral gyrus (z_max_ = 4.49, p = 1.08e-15, xyz = [14 -10 64]), right precentral and postcentral gyrus (z_max_ = 4.34, p = 1.14e-08, xyz = [22 -34 74]), left (z_max_ = 4.56, p = 0.000213, xyz = [-54 -24 22]) and right (z_max_ = 4.28, p = 7.75e-07, xyz = [62 -24 30]) supramarginal gyrus/ operculum, left (z_max_ = 4.82, p = 0.000279, xyz = [-48 4 6]) and right (z_max_ = 4.07, p = 5.3e-05, xyz = [26 2 10]) striatum and operculum, and bilateral cerebellum (z_max_ = 4.67, p = 3.58e-07, xyz = [10 -54 -14]) (see Fig. S17B). These regions also tended to be more active for Go than NoGo responses, corroborating the notion of theta reflecting evidence for active responses.

Notably, correlations with trial-by-trial theta power were not restricted to the striatum, but also occurred for other motor regions such as the ACC and motor cortices. These differences to the results of the fMRI-inspired EEG analyses might be attributable to methodological differences between both approaches: First, if theta power reflects global trial-by-trial brain activity associated with motion, this signal property will lead to correlations with BOLD in several motor regions in EEG-inspired fMRI analyses. In contrast, in fMRI-inspired analyses, such variance will be shared among regressors and thus be attributed to neither of them. Second, for EEG-inspired fMRI analyses, we created trial-by-trial indicators of theta power within a broad time window, which potentially mixes distinct events in theta that reflect activity in different brain regions. Thus, this approach might lead to correlations with BOLD in several brain regions that actually perform different computations at different time points. Following this reasoning, fMRI-inspired analyses have the advantage of a) identifying which regions uniquely predict time-frequency power beyond variance shared among regions, and b) unmixing different regions predicting time-frequency power at different time points.

| 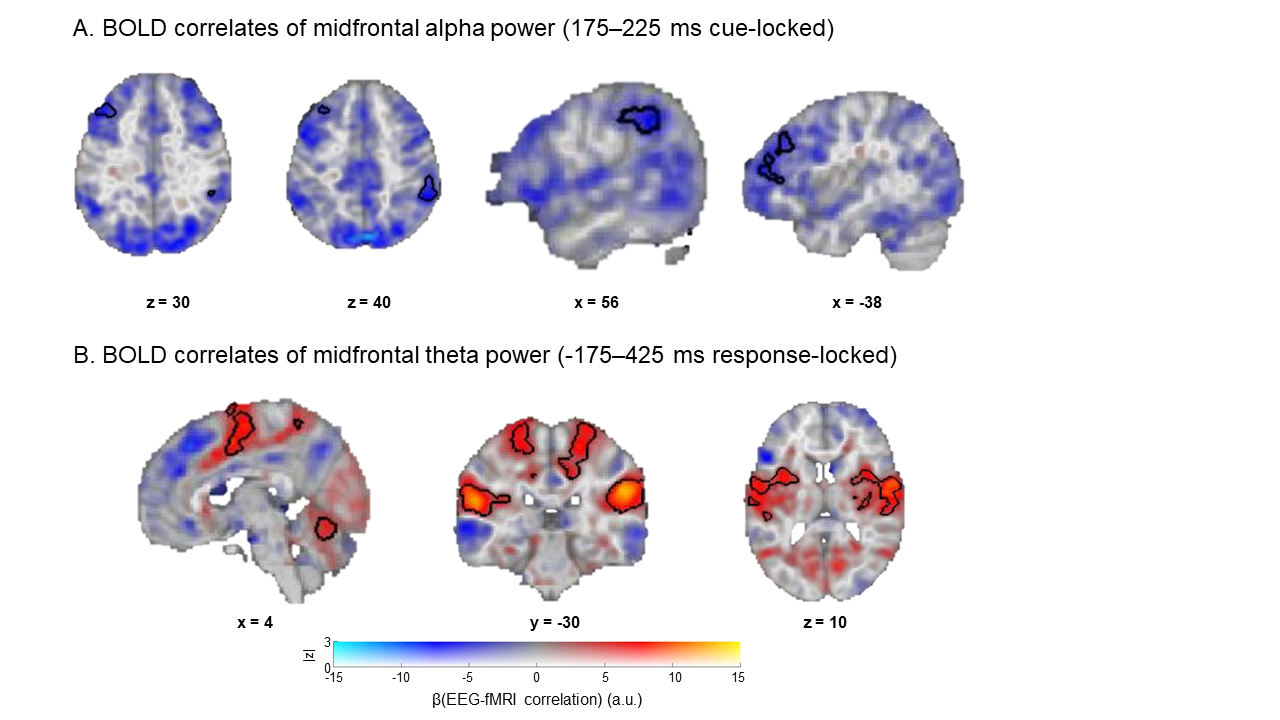 |
| --- |
| *Figure S17*. Trial-by-trial time-frequency power as a predictor of BOLD in an fMRI GLM. (A) Trial-by-trial midfrontal alpha power correlated significantly negatively with BOLD in two clusters in left middle frontal gyrus and in right supramarginal gyrus. Sub-threshold, it correlated negatively with extended regions in fronto-parietal and dorsal attention network. (B) Trial-by-trial midfrontal theta power correlated significantly positively with BOLD in pre-SMA, ACC, bilateral precentral and postcentral gyrus, superior parietal lobule, precuneous, bilateral operculum, bilateral putamen, and bilateral cerebellum. |

# References

Bland, B. H., & Oddie, S. D. (2001). Theta band oscillation and synchrony in the hippocampal formation and associated structures: The case for its role in sensorimotor integration. *Behavioural Brain Research*, *127*(1–2), 119–136. doi: 10.1016/S0166-4328(01)00358-8

Canolty, R. T., Edwards, E., Dalal, S. S., Soltani, M., Nagarajan, S. S., Kirsch, H. E., … Knight, R. T. (2006). High gamma power is phase-locked to theta oscillations in human neocortex. *Science*, *313*(5793), 1626–1628. doi: 10.1126/science.1128115

Caplan, J. B., Madsen, J. R., Schulze-Bonhage, A., Aschenbrenner-Scheibe, R., Newman, E. L., & Kahana, M. J. (2003). Human θ oscillations related to sensorimotor integration and spatial learning. *The Journal of Neuroscience*, *23*(11), 4726–4736. doi: 10.1523/JNEUROSCI.23-11-04726.2003

Cavanagh, J. F., Eisenberg, I., Guitart-Masip, M., Huys, Q. J. M., & Frank, M. J. (2013). Frontal theta overrides Pavlovian learning biases. *Journal of Neuroscience*, *33*(19), 8541–8548. doi: 10.1523/JNEUROSCI.5754-12.2013

Cavanagh, J. F., & Frank, M. J. (2014). Frontal theta as a mechanism for cognitive control. *Trends in Cognitive Sciences*, *18*(8), 414–421. doi: 10.1016/j.tics.2014.04.012

Cavanagh, J. F., Wiecki, T. V, Cohen, M. X., Figueroa, C. M., Samanta, J., Sherman, S. J., & Frank, M. J. (2011). Subthalamic nucleus stimulation reverses mediofrontal influence over decision threshold. *Nature Neuroscience*, *14*(11), 1462–1467. doi: 10.1038/nn.2925

Cohen, M. X. (2014). A neural microcircuit for cognitive conflict detection and signaling. *Trends in Neurosciences*, *37*(9), 480–490. doi: 10.1016/j.tins.2014.06.004

Cohen, M. X., & Cavanagh, J. F. (2011). Single-trial regression elucidates the role of prefrontal theta oscillations in response conflict. *Frontiers in Psychology*, *2*(FEB), 1–12. doi: 10.3389/fpsyg.2011.00030

Cohen, M. X., & Donner, T. H. (2013). Midfrontal conflict-related theta-band power reflects neural oscillations that predict behavior. *Journal of Neurophysiology*, *110*(12), 2752–2763. doi: 10.1152/jn.00479.2013

DeCoteau, W. E., Thorn, C., Gibson, D. J., Courtemanche, R., Mitra, P., Kubota, Y., & Graybiel, A. M. (2007a). Learning-related coordination of striatal and hippocampal theta rhythms during acquisition of a procedural maze task. *Proceedings of the National Academy of Sciences of the United States of America*, *104*(13), 5644–5649. doi: 10.1073/pnas.0700818104

DeCoteau, W. E., Thorn, C., Gibson, D. J., Courtemanche, R., Mitra, P., Kubota, Y., & Graybiel, A. M. (2007b). Oscillations of local field potentials in the rat dorsal striatum during spontaneous and instructed behaviors. *Journal of Neurophysiology*, *97*(5), 3800–3805. doi: 10.1152/jn.00108.2007

Donner, T. H., Siegel, M., Fries, P., & Engel, A. K. (2009). Buildup of choice-predictive activity in human motor cortex during perceptual decision making. *Current Biology*, *19*(18), 1581–1585. doi: 10.1016/j.cub.2009.07.066

Fellner, M.-C., Volberg, G., Mullinger, K. J., Goldhacker, M., Wimber, M., Greenlee, M. W., & Hanslmayr, S. (2016). Spurious correlations in simultaneous EEG-fMRI driven by in-scanner movement. *NeuroImage*, *133*, 354–366. doi: 10.1016/j.neuroimage.2016.03.031

Frank, M. J., Gagne, C., Nyhus, E., Masters, S., Wiecki, T. V., Cavanagh, J. F., & Badre, D. (2015). fMRI and EEG predictors of dynamic decision parameters during human reinforcement learning. *Journal of Neuroscience*, *35*(2), 485–494. doi: 10.1523/JNEUROSCI.2036-14.2015

Hunt, L. T., Kolling, N., Soltani, A., Woolrich, M. W., Rushworth, M. F. S., & Behrens, T. E. J. (2012). Mechanisms underlying cortical activity during value-guided choice. *Nature Neuroscience*, *15*(3), 470–476. doi: 10.1038/nn.3017

Jurkiewicz, M. T., Gaetz, W. C., Bostan, A. C., & Cheyne, D. (2006). Post-movement beta rebound is generated in motor cortex: Evidence from neuromagnetic recordings. *NeuroImage*, *32*(3), 1281–1289. doi: 10.1016/j.neuroimage.2006.06.005

Landau, A. N., Schreyer, H. M., van Pelt, S., & Fries, P. (2015). Distributed attention is implemented through theta-rhythmic gamma modulation. *Current Biology*, *25*(17), 2332–2337. doi: 10.1016/j.cub.2015.07.048

Maris, E., van Vugt, M., & Kahana, M. (2011). Spatially distributed patterns of oscillatory coupling between high-frequency amplitudes and low-frequency phases in human iEEG. *NeuroImage*, *54*(2), 836–850. doi: 10.1016/j.neuroimage.2010.09.029

Murphy, P. R., Robertson, I. H., Harty, S., & O’Connell, R. G. (2015). Neural evidence accumulation persists after choice to inform metacognitive judgments. *ELife*, *4*, 1–23. doi: 10.7554/eLife.11946

O’Connell, R. G., Dockree, P. M., & Kelly, S. P. (2012). A supramodal accumulation-to-bound signal that determines perceptual decisions in humans. *Nature Neuroscience*, *15*(12), 1729–1735. doi: 10.1038/nn.3248

Polanía, R., Krajbich, I., Grueschow, M., & Ruff, C. C. (2014). Neural oscillations and synchronization differentially support evidence accumulation in perceptual and value-based decision making. *Neuron*, *82*(3), 709–720. doi: 10.1016/j.neuron.2014.03.014

Ritter, P., Moosmann, M., & Villringer, A. (2009). Rolandic alpha and beta EEG rhythms’ strengths are inversely related to fMRI-BOLD signal in primary somatosensory and motor cortex. *Human Brain Mapping*, *30*(4), 1168–1187. doi: 10.1002/hbm.20585

Salmelin, R., Forss, N., Knuutila, J., & Hari, R. (1995). Bilateral activation of the human somatomotor cortex by distal hand movements. *Electroencephalography and Clinical Neurophysiology*, *95*(6), 444–452. doi: 10.1016/0013-4694(95)00193-X

Salmelin, R., Hämäläinen, M., Kajola, M., & Hari, R. (1995). Functional segregation of movement-related rhythmic activity in the human brain. *NeuroImage*. doi: 10.1006/nimg.1995.1031

Sanes, J. N., & Donoghue, J. P. (1993). Oscillations in local field potentials of the primate motor cortex during voluntary movement. *Proceedings of the National Academy of Sciences*, *90*(10), 4470–4474. doi: 10.1073/pnas.90.10.4470

Stolk, A., Brinkman, L., Vansteensel, M. J., Aarnoutse, E., Leijten, F. S. S., Dijkerman, C. H., … Toni, I. (2019). Electrocorticographic dissociation of alpha and beta rhythmic activity in the human sensorimotor system. *ELife*, *8*, 636241. doi: 10.1101/636241

Swart, J. C., Frank, M. J., Määttä, J. I., Jensen, O., Cools, R., & den Ouden, H. E. M. (2018). Frontal network dynamics reflect neurocomputational mechanisms for reducing maladaptive biases in motivated action. *PLOS Biology*, *16*(10), e2005979. doi: 10.1371/journal.pbio.2005979

van Vugt, M. K., Simen, P., Nystrom, L. E., Holmes, P., & Cohen, J. D. (2012). EEG oscillations reveal neural correlates of evidence accumulation. *Frontiers in Neuroscience*, *6*(JULY), 1–13. doi: 10.3389/fnins.2012.00106

Wessel, J. R., Ghahremani, A., Udupa, K., Saha, U., Kalia, S. K., Hodaie, M., … Chen, R. (2016). Stop-related subthalamic beta activity indexes global motor suppression in Parkinson’s disease. *Movement Disorders*, *31*(12), 1846–1853. doi: 10.1002/mds.26732

Wessel, J. R., Waller, D. A., & Greenlee, J. D. (2019). Non-selective inhibition of inappropriate motor-tendencies during response-conflict by a fronto-subthalamic mechanism. *ELife*, *8*, 1–26. doi: 10.7554/eLife.42959

Womelsdorf, T., Vinck, M., Leung, L. S., & Everling, S. (2010). Selective theta-synchronization of choice-relevant information subserves goal-directed behavior. *Frontiers in Human Neuroscience*, *4*(November), 1–13. doi: 10.3389/fnhum.2010.00210
